# Radical Scavenging Potential of Ginkgolides and Bilobalide: Insight from Molecular Modeling

**DOI:** 10.3390/antiox12020525

**Published:** 2023-02-19

**Authors:** Davide Zeppilli, Giovanni Ribaudo, Nicola Pompermaier, Andrea Madabeni, Marco Bortoli, Laura Orian

**Affiliations:** 1 Dipartimento di Scienze Chimiche, Università degli Studi di Padova, Via Marzolo 1, 35129 Padova, Italy; 2 Dipartimento di Medicina Molecolare e Traslazionale, Università degli Studi di Brescia, Viale Europa 11, 25123 Brescia, Italy; 3 Department of Chemistry and Hylleraas Centre for Quantum Molecular Sciences, University of Oslo, 0315 Oslo, Norway

**Keywords:** antioxidants, bilobalide, ginkgolides, hydrogen-atom transfer, single-electron transfer, scavengers, terpenoids

## Abstract

The reactive oxygen species (ROS) scavenging capacities of ginkgolides and bilobalide, which are the peculiar constituents of the extract of *Ginkgo biloba*, are investigated in silico (level of theory: (SMD)-M06-2X/6-311+G(d,p)//M06-2X/6-31G(d)). Unlike other popular antioxidant natural substances, the carbon backbones of these compounds are entirely aliphatic and exclusively single C–C bonds are present. The selectivity for alkoxyl radicals via hydrogen-atom transfer (HAT) is assessed; importantly, the scavenging of peroxyl radicals is also possible from a peculiar site, here labeled C10 both for ginkgolides and bilobalide. The energetics are described in detail, and the analysis discloses that the studied compounds are powerful scavengers, with thermodynamic and kinetic properties similar to those of Trolox and melatonin, and that, in addition, they display selectivity for peroxyl radicals. These are all chemical-reactivity features contributing to the therapeutic action of the extract of *G. biloba*.

## 1. Introduction

Radical species, due to the intrinsic instability of their electronic configurations, are naturally endowed with a high tendency to be reduced by reacting with other species, including biomolecules [1]. Thus, they represent a threat to cellular (macro)molecular targets, such as proteins, lipids and nucleic acids [2]. In the biological environment, the formation of radicals cannot be fully halted since they play several roles in cellular metabolism as reaction byproducts or redox signaling molecules. Thus, it is of outmost importance for the antioxidant defense of cells to keep up with the production of ROS when they suddenly increase due to stressors and external factors [3].

Within the cell, a fine equilibrium between pro-oxidant and antioxidant species exists. When this equilibrium is pathologically and uncontrollably altered in favor of the former species, a condition known as “oxidative stress” occurs [4], where oxidative damage jeopardizes the structures and functions of phospholipids, nucleic acids and proteins. These events have been linked to the onset and progression of several diseases, such as neurodegenerative disorders [5], cardiovascular diseases [6] and some types of cancer [7], as well as mood disorders [8] and schizophrenia [9].

**Scheme 1:**
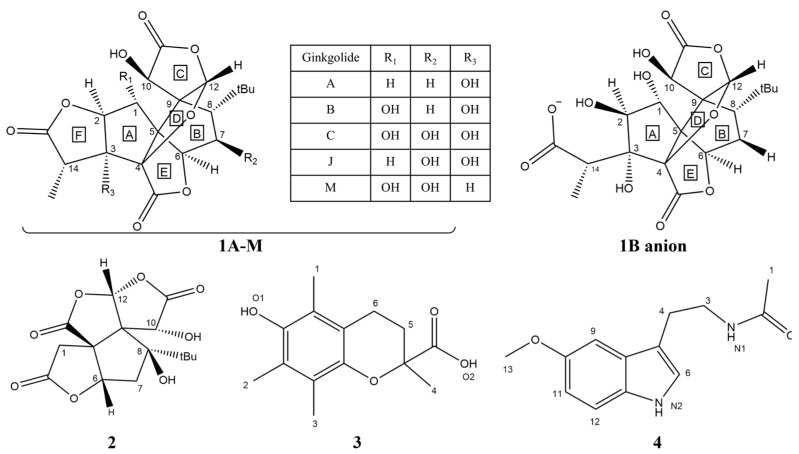
Chemical structures of ginkgolides A (**1A**), B (**1B**), C (**1C**), J (**1J**) and M (**1M**), the most stable monoanionic form of the **1B anion**; bilobalide (**2**); Trolox (**3**); and melatonin (**4**) [17].

Antioxidants mitigate oxidative stress conditions. Endogenous antioxidants include enzymes, such as superoxide dismutase (SOD), which catalyzes the dismutation of O_2_^•−^ into O_2_ and H_2_O_2_ [10] (the latter being further inactivated by catalase (CAT) into O_2_ and H_2_O [11]), and glutathione peroxidase (GPx), which catalyzes the reduction of H_2_O_2_ and hydroperoxides to water/alcohols [12,13], but also low-molecular-weight compounds, such as glutathione [14] and melatonin (**4**, Scheme 1) [15]. Conversely, exogenous antioxidants are substances which can be ingested through the diet (e.g., carotenoids, flavonoids, vitamins C and E, and polyphenols) [16] and which typically act as scavengers of harmful radicals.

In this work, the focus is on the scavenging potential of natural components of *Ginkgo biloba*. In this connection, ginkgolides (**1A–M**, Scheme 1) are a family of C20 terpenic trilactones present in the leaves and root bark of *G. biloba* [18]. They possess more than one hydroxyl group, but, unlike polyphenols, their structures are completely saturated, and this results in completely different reactivity profiles. Although they were isolated in the 1960s, they attracted the attention of medicinal chemists some years later, when *G. biloba* leaf extracts were commercialized as a treatment for dementia and intermittent claudication due to their positive effect on central and peripheral blood circulation [19]. Currently, ginkgolides attract great interest due to the plethora of their biological activities, including anti-inflammatory, anticancer, antiatherosclerosis, antithrombosis and hepatoprotective effects [20,21]. Concerning their role in the central nervous system (CNS), ginkgolides influence the action of glutamate and dopamine, inflammatory processes and several stages of oxidative stress and exert anxiolytic-like activities and neuroprotective effects [22,23]. However, the toxicological profiles of these natural compounds still need to be fully elucidated [20].

Bilobalide (**2**, Scheme 1) is another widely studied bioactive component of *G. biloba*. The pharmacological features and the toxicity profile of this sesquiterpene trilactone have been recently reviewed by Lu et al. [24], who overviewed the several molecular targets of this natural molecule. Bilobalide bears neuroprotective properties and has been studied for its potential use against Parkinson’s and Alzheimer’s diseases [24]. These effects appear to be mediated by the antioxidant activity of bilobalide, which has been demonstrated to combat oxidative stress in animal models through interference with the Nrf2 pathway and via the enhancement of SOD and CAT enzymes [25,26]. Moreover, in a previous study, Lu et al. showed that bilobalide reduces oxidative damage in human melanocytes. Interestingly, the authors found that the compound promotes the expression of antioxidant enzymes CAT and GPx [27]. Furthermore, Chandrasekaran and co-workers [28] reported that bilobalide protects against neuronal death in brain ischemia via a complex mechanism which includes inhibition of free-radical generation as well as ROS scavenging.

*G. biloba* extracts have been reported to possess radical scavenging activity towards superoxide, hydroperoxyl and hydroxyl radicals; however, it is not clear which components of the extracts are responsible for this activity [17], as mixtures of different ginkgolides, bilobalide and flavonoids, which also have antioxidant effects, are present [29,30]. While the antioxidant scavenging activity of glycosylated flavonoids has been well established in silico as well as experimentally [31], for ginkgolides and bilobalide, which are the peculiar constituents of *G. biloba* extract, no theoretical rationalization rooted in the chemical features of these compounds has been attempted so far.

Ginkgolides and bilobalide are hydrophobic compounds sparingly soluble in water [19]. Their chemical features account for their affinity to cell membranes, where they can express their antioxidant effects by protecting unsaturated fatty acids from peroxyl radicals [32]. Ginkgolide B is arguably the most widely studied derivative of this class, and protonation states for this molecule have been extensively investigated experimentally as well as in vivo [33,34]. The three acid-dissociation constants for ginkgolide B are pK_a1_ = 7.14, pK_a2_ = 8.60 and pK_a3_ = 11.89, suggesting that the molecule exists in both neutral and monoanionic forms in physiological conditions. More specifically, Suehiro et al. [35] reported that, after administration, neutral ginkgolide B is rapidly distributed to various organs, including the liver, intestine and stomach, and that the ionized form is retained in plasma, while thereafter the ratios between the species slowly shift towards equilibrium. However, the authors reported low blood–brain barrier (BBB) permeability for this compound [35]. Through potentiometric analysis and NMR investigations, Zekri et al. have shown that, in physiological conditions, the species present in the most significant concentrations are the neutral form and the monoanionic form that originates by the hydrolysis of the F ring (**1B anion**, Scheme 1) [33].

Bilobalide is structurally related to the ginkgolides, as it is a trilactone which has similar chemical features [36]. Concerning its pharmacokinetic properties, a slightly longer half-life was observed for bilobalide with respect to ginkgolides A and B in rats [37].

The complex phenomenon of antioxidant action is rooted in elementary chemical reactions and an understanding of them is essential to gain insight into the ROS scavenging potential of classes of substances which can be employed as supplements or as drugs. Protocols in silico have been applied with success to a variety of classes of compounds, ranging from food constituents [38] to drugs [39,40,41,42,43,44].

**Scheme 2:**
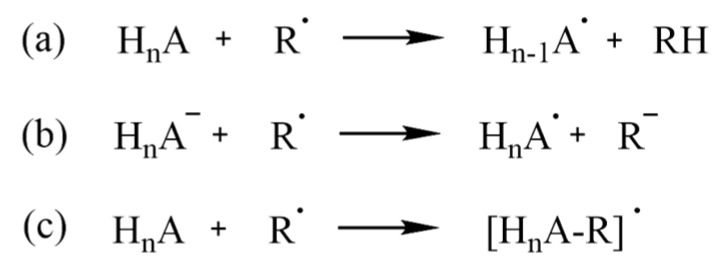
(**a**) HAT, (**b**) SET and (**c**) RAF mechanisms for a generic antioxidant, H_n_A.

Radical scavenging can occur via multiple mechanisms, depending on different conditions; competition among different mechanisms is also possible. The well-known HAT (hydrogen-atom transfer, Scheme 2a) entails the transfer of a hydrogen atom to the radical, which is thus quenched. If multiple reactive sites are present in the molecule, an equal number of different radical products (H_n-1_A^•^) can be formed. SET (single-electron transfer, Scheme 2b) is better suited for negatively charged scavengers, which, however, can still react via HAT; RAF (radical adduct formation, Scheme 2c) usually involves an aromatic ring, or, in general, unsaturated bonds to which ROS are added, and can also result, like HAT, in multiple products, depending on the structure of the scavenger [45].

In this work, we investigated the radical scavenging activities of the five ginkgolides labelled A, B, C, J and M (**1A–M**, Scheme 1), the most stable monoanionic form of ginkgolide B (**1B anion**, Scheme 1), as well as that of bilobalide (**2**, Scheme 1), describing the thermodynamics and kinetics of the elementary reactions between these compounds and the five ROS (see Results and Discussion); the effect of polar and apolar environments are also discussed. The scavenging potential of these molecules was evaluated according to a state-of-the-art computational protocol [39,40,46]. This protocol, which relies on quantum-mechanical DFT calculations, evaluates the feasibility of a selection of radical scavenging mechanisms for a particular substrate, employing calculations of both reaction and activation energies. These can be compared to reference data obtained for extensively studied antioxidants, such as Trolox (**3**), for the elaboration of a relative quantification scale.

## 2. Materials and Methods

Full geometry optimizations of reactants, products and transition states were performed in the gas phase using the M06-2X functional [47] combined with the 6-31G(d) basis set, as implemented in Gaussian16 [48]. Spin contamination was checked for the doublet ground-state species and was found to be negligible. To verify the nature of the stationary points and to obtain thermodynamic corrections at 298 K and 1 atm, frequency calculations were carried out at M06-2X/6-31G(d). This procedure ascertained that a single imaginary frequency with the correct vibrational mode was present in the transition-state structures and that only real frequencies were present in the minimum-energy structures. Afterwards, to achieve a more accurate estimate, single-point energy calculations were computed for the optimized structures at the M06-2X/6-311+G(d,p) level of theory in gas phase, and, subsequently, in water and benzene, using the continuum solvation model SMD [49] (level of theory: (SMD)-M06-2X/6-311+G(d,p)//M06-2X/6-31G(d)). Water and benzene were chosen to mimic polar and non-polar environments, respectively. This combination of functional and basis sets was previously validated and employed successfully in other studies [39,40,41,45,50]. To obtain gas-phase Gibbs free energies, thermochemical corrections ($\Delta G_{gas,\;\;RRHO}^{o}$) should be added to electronic energies in the gas phase ($E_{gas}$), according to Equation (1):

(1) $$G_{gas}^{o}\left(X\right)=E_{gas}\left(X\right)+\Delta G_{gas,\;\;RRHO}^{o}\left(X\right)\;$$

Since the structures were not optimized in solution, we approximated the thermochemical correction to the electronic energy in the solution to be equal to that of the gas phase, i.e.:

(2) $$G_{soln}^{o}\left(X\right)=E_{soln}\left(X\right)+\Delta G_{gas,RRHO}^{o}\left(X\right)-RTln\left(V^{-1}\right)\;$$

The last term of the right hand side of Equation (2) takes into account the standard state conversion from a concentration of 1 atm to a concentration of 1 M, since most of the experimental free energies in solution refer to a 1 M standard state [51].

The activation energies were calculated for the most and the least reactive sites of the neutral ginkgolide B and for the most reactive site of the monoanionic ginkgolide B (identified on the basis of $\Delta G_{HAT}^{o}$); these were relative to the free reactants in the gas phase (Equation (3)) as well as in the solvent:

(3) $$\Delta G_{HAT}^{‡}=G_{i}^{o}\left(TS\right)-G_{i}^{o}\left(R\right)\;$$

where *i* refers to the environment (gas phase, water or benzene).

This choice was made since HAT reactions were found to follow the Hammond postulate for different classes of molecules [39,40,52]; thus, the most favored reactions were expected to have the lowest activation energies. Electron spin densities, defined as the spatial differences between the densities of α spin electrons and β spin electrons, were calculated with Mulliken population analysis for selected sites of **1B** and **1B anion**. Spin density surfaces were drawn for selected structures with Avogadro [53], with an isodensity value of 0.02.

## 3. Results and Discussion

*G. biloba*, its extracts and its molecular components have been widely investigated for their antioxidant properties, and several contributions focused on this subject can be found in the literature.

Droy-Lefaix reported the effects of *G. biloba* extract EGb 761 in animal models, highlighting that it can contribute to the quenching of the superoxide anion and hydroxyl and peroxyl radicals, thus improving mitochondrial respiratory-chain function. Accordingly, the author suggested that flavonoids and terpenes may represent the antioxidant constituents. Moreover, the extract showed protective effects against lipoperoxidation and subsequent damage, paving the way for neuroprotective functions [54].

The protective antioxidant effects of EGb 761 in the context of neurodegenerative disease were also explored by Liu et al., who confirmed that the extract inhibited lipid peroxidation of microsomes and protected cells against H_2_O_2_-induced oxidative damage [55].

More recently, Singh et al. reviewed the neuroprotective and antioxidant effects of *G. biloba* extract against Alzheimer’s disease, and the reader is invited to refer to this contribution for a more detailed overview of this topic [56]. The main mechanisms responsible for antioxidant and neuroprotective activity involve the direct reduction of ROS but also the enhancement of antioxidant enzymes [56].

Accordingly, Di Meo et al. showed that EGb 761, acting as an intracellular antioxidant, protects against oxidative-stress-induced apoptotic cell death, supporting its potential role as a nutraceutical for the prevention and treatment of neurodegenerative diseases [57].

Multiple scavenging pathways can be viable for the same molecule, though it is convenient to identify the prevalent ones [41,50]. Based on previous studies of different archetypal structures [45], in the cases of the neutral ginkgolides **1A–M** and bilobalide **2**, it is reasonable to assume that HAT is prevalent over SET, but competition between the two mechanisms is considered for the monoanionic-form **1B anion**. Conversely, RAF is not expected to be relevant to the compounds studied here because no unsaturated moieties are present in their structures; hence, this mechanism was not evaluated.

In this work, we investigated the radical scavenging activities of **1A–M**, the **1B anion** and **2** and described the thermodynamics and kinetics of HAT elementary reactions at all the available sites. Gibbs free reaction and activation energies were accurately computed using a computational protocol based on DFT.

The different sites where HAT can occur are labeled with the number of C (or C–O) atoms bonded to the H atom, which is transferred to the ROS (the numeration refers to Scheme 1). According to this choice, HAT from C10 refers to the cleavage of a C_10_–H bond; HAT from C10OH, instead, refers to the cleavage of a O–H bond of C_10_O–H.

The ROS here considered are ^•^OH, ^•^OOH, ^•^OCH_3_, ^•^OOCH_3_ and ^•^OOCH=CH_2_. Hydroxyl (^•^OH) and methoxy (^•^OCH_3_) radicals display high reactivities, with ^•^OH being the most reactive due to the single oxygen atom bearing an unpaired electron and having a shorter half-life (i.e., 10^−9^ s [58]), while the peroxyl radicals ^•^OOH and ^•^OOCH_3_ are less reactive and are characterized by longer half-lives (i.e., a few seconds [59]), which allows them to diffuse and to damage molecular structures that are not in close proximity. Additionally, ^•^OOCH=CH_2_ mimics an unsaturated fatty acid peroxyl radical, which is the product of the oxidation of phospholipids.

### 3.1. HAT Energetics for Ginkgolides

**Figure 1:**
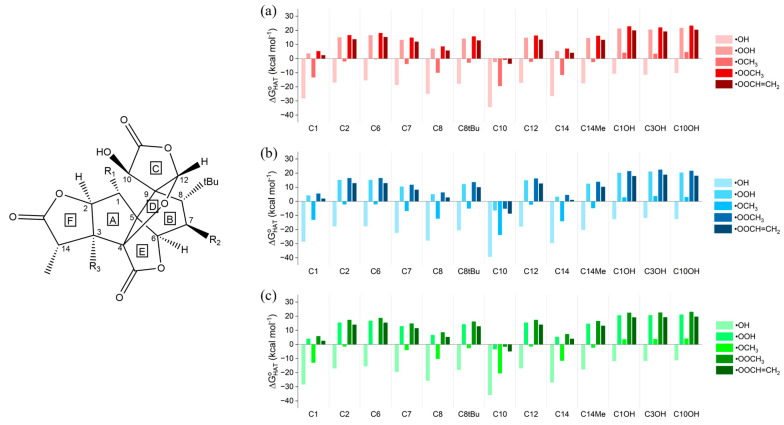
Gibbs free reaction energies ($\Delta G_{HAT}^{o}$) computed in (**a**) gas phase, (**b**) water and (**c**) benzene for the scavenging of ^•^OH, ^•^OOH, ^•^OCH_3_, ^•^OOCH_3_ and ^•^OOCH=CH_2_ via HAT from all the available sites of **1B**. Level of theory: (SMD)-M06-2X/6-311+G(d,p)//M06-2X/6-31G(d).

Thermodynamic data for the neutral ginkgolides, which revealed the feasibility of the reactions, were computed for all five neutral ginkgolides. Only those of **1B** are discussed; the others, the analysis of which led to very similar conclusions, are included in the Supplementary Materials and are briefly commented on at the end of this paragraph. Gibbs free reaction energies ($\Delta G_{HAT}^{o}$) of HATs involving **1B** and the five selected ROS in gas phase, in water and in benzene are shown in Figure 1.

Positions 4, 5 and 9 (Scheme 1) are not eligible for HAT. Moreover, all H atoms found in methyl groups, such as at position 14 or in the *tert*-butyl group at position 8, are equivalent for HAT.

**Figure 2:**
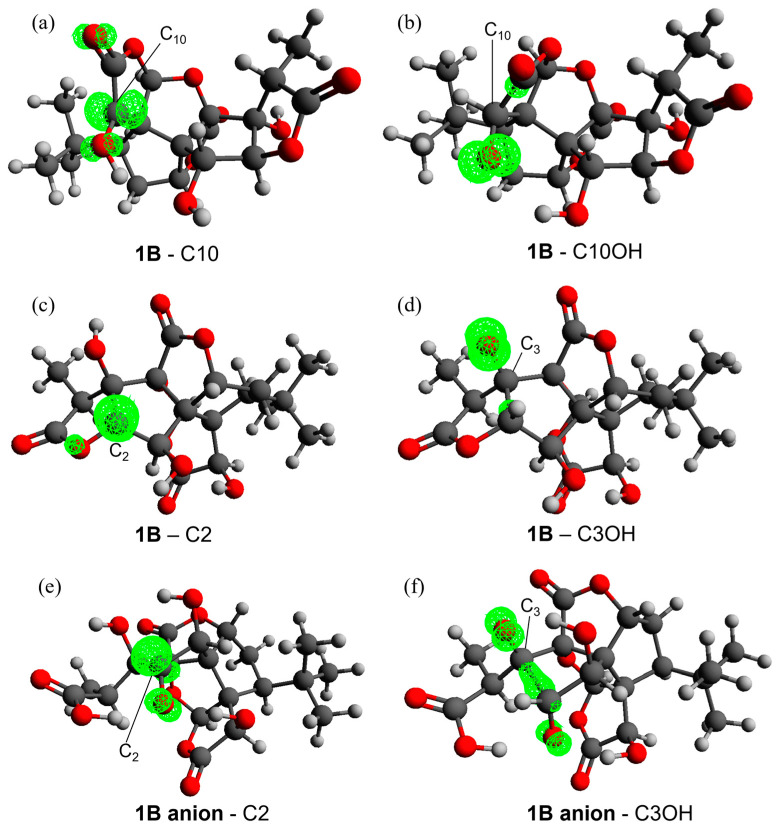
Spin densities on the radical products of **1B** when HAT occurs from C10 (**a**), C10OH (**b**), C2 (**c**) and C3OH (**d**) and on the **1B anion** radical when HAT occurs from C2 (**e**) and C3OH (**f**) with an isosurface value of 0.02. Level of theory: M06-2X/6-311+G(d,p)//M06-2X/6-31G(d).

The gas-phase data (Figure 1a) show that all HATs to ^•^OH are moderately or even strongly exergonic ($\Delta G_{HAT}^{o}$ values between −10.27 and −34.38 kcal mol^−1^); ^•^OCH_3_, as expected, is the second most reactive ROS, associated with negative—although smaller in absolute terms when compared to those computed for ^•^OH—$\Delta G_{HAT}^{o}$ values for all sites, with the exception of the three OH positions. ^•^OOH is noticeably less reactive than the two alkoxyl radicals, having an exergonic HAT only from site C10. The same behavior can be observed for ^•^OOCH_3_ and ^•^OOCH=CH_2_, both still being able to undergo a favorable HAT only from site C10. Thus, HAT from C10 is exergonic for all ROS, indicating that the radical product of this reaction is more stable than the free ROS. C1 and C14 are the second and third most reactive sites, respectively, although their reactivities are not sufficient to allow for exergonic HATs to the three peroxyl radicals. Hydroxyl groups (C1OH, C3OH and C10OH) react spontaneously only with ^•^OH, while the quenching of all the other radicals is associated with a positive $\Delta G_{HAT}^{o}$ value (^•^OCH_3_ still shows a less unfavorable reactivity compared to peroxyl radicals but remains endergonic). The stability of the HAT radical products can be rationalized by inspecting the electronic spin density surfaces shown in Figure 2a,b: a larger delocalization is present in the product of HAT from C10 than in C10OH. C10, which is in the α position with respect to a lactone carbonyl group, also bears a hydroxyl group, which can delocalize the spin density efficiently. It is also noteworthy that hydroxyl groups show a spontaneous tendency for HAT only with the most reactive ROS, which contrasts with the common behavior of polyphenols, such as anthocyanidins [34,46]. This major difference is easily explained by comparing the two very different chemical scaffolds: ginkgolides are completely aliphatic, whereas polyphenols are aromatic. The presence of a highly delocalized π system in the latter class of compounds boosts the reactivity of hydroxyl groups towards HAT.

The scavenging potentials of ginkgolides A, C, J and M were also investigated, following the same protocol (Tables S2–S5, Figures S1–S4). These data do not show any remarkable differences in trends or absolute values; thus, here and in the following, only ginkgolide B data are discussed. The different substitutions in **1A–M** at the three sites C1, C3 and C7 have little to no effect on overall reactivity. The OH sites are still the least reactive, and HAT from C10 remains the only reaction with a negative $\Delta G_{HAT}^{o}$ value for the scavenging of peroxyl radicals (−7.11 kcal mol^−1^ < $\Delta G_{HAT,water}^{o}$ < −6.12 kcal mol^−1^ for ^•^OOH). The only substantial difference is that the presence of a hydroxyl group increases the delocalization of the spin density on the carbon in α at R_1_, R_2_, R_3_=OH. This may lead to lower, yet still not negative, $\Delta G_{HAT}^{o}$ values with the peroxyl radicals, as can be seen for C1 in ginkgolides B and C. Moreover, these substitutions show no major effects on the reactivity of C10, the most reactive site, as is to be expected given the absence of large, conjugated regions.

The presence of a solvent does not modify the trends stated above for ginkgolide B. Stabilization effects attributed to solvation are slight to nonexistent in the case of a HAT reaction in benzene (Figure 1c), while water is appreciably more efficient in stabilizing the ginkgolide radicals (Figure 1b), with a maximum stabilization energy ($\Delta G_{HAT,\;water}^{o}-\Delta G_{HAT}^{o}$) reaching −5.07 kcal mol^−1^. Analogous considerations are valid for the other ginkgolides (see the Supplementary Materials).

### 3.2. HAT Energetics for the Anionic Form of Ginkgolide B

**Figure 3:**
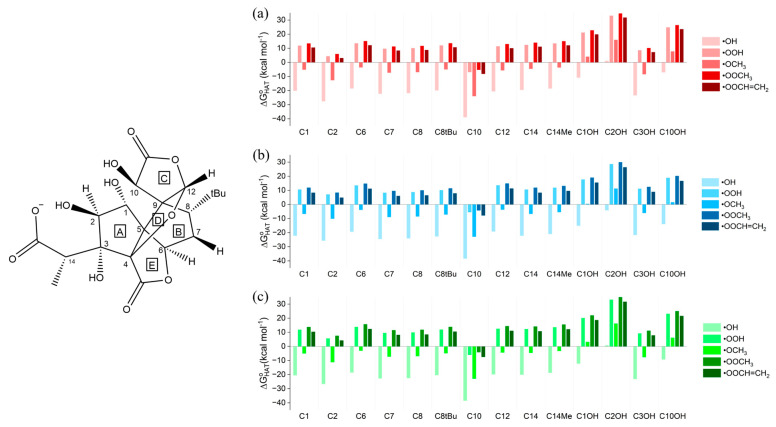
Gibbs free reaction energies ($\Delta G_{HAT}^{o}$) computed in (**a**) gas phase, (**b**) water and (**c**) benzene for the scavenging of ^•^OH, ^•^OOH, ^•^OCH_3_, ^•^OOCH_3_ and ^•^OOCH=CH_2_ via HAT from all the available sites of the **1B anion**. Level of theory: (SMD)-M06-2X/6-311+G(d,p)//M06-2X/6-31G(d).

Ginkgolide B is the most studied ginkgolide and it is also commercial. It has interesting and well-established acid behavior [33], and in physiological conditions the neutral form coexists with a monoanionic form due to the hydrolysis of the F ring (**1B anion**, Scheme 1). In this form, ginkgolide B may still act as a radical scavenger via the HAT mechanism, but also via SET (Scheme 2b), since it is negatively charged. Thus, we analyzed first the HAT mechanism for all the possible sites (the ones of the neutral form plus C2OH) and then the SET mechanism, which is a single reaction for each ROS. Gibbs free reaction energies ($\Delta G_{HAT}^{o}$) computed in gas phase, in water and in benzene are shown in Figure 3. Due to the similar reactivities of the five ginkgolides in their neutral forms, only the monoanionic form of ginkgolide B was analyzed.

The gas-phase data (Figure 3a, Table S6) show a thermodynamic trend analogous to that reported for the neutral system; HAT to ^•^OH is the most favored process, followed by HAT to ^•^OCH_3_. In the former case, all reactions are exergonic, but at the new C2OH site the reaction is isergonic. Conversely, a positive $\Delta G_{HAT}^{o}$ value is associated with the second ROS only in the case of all the OH sites, with the exception of C3OH. The three peroxyl radicals are significantly less reactive, and they still react spontaneously only at the C10 site, which remains the energetically most favored site for HAT. Conversely, the C2OH site is associated with the most unfavored HAT, suggesting that the radical ginkgolide product must be very unstable. The solvation effect is still very modest in the case of benzene, while in water the maximum stabilization energy ($\Delta G_{HAT,\;water}^{o}-\Delta G_{HAT}^{o}$) is −6.88 kcal mol^−1^.

$\Delta G_{HAT}^{o}$ values for **1B** and the **1B anion** are quite similar, with few differences between them. The HATs from the most reactive site, i.e., C10, are more exergonic in the monoanionic form, with a difference of −4.54 kcal mol^−1^, regardless of the ROS. C2 and C3OH sites are associated with a significantly more favored reaction in the monoanionic form, while the reactions from C1 and C14 become less favored moving from the neutral to the anionic form. These considerations are valid for all ROS; the $\Delta G_{HAT}^{o}$ values for the scavenging of **·**OH via HAT are reported in Table 1. These differences are maintained in benzene, while in water they decrease in absolute values. In particular, $\Delta \Delta G_{HAT}^{o}$ from C10 becomes negligible, while the reactivity differences of the other sites of the two forms of ginkgolide B mentioned above remain significant (Table S7).

The better stabilization of the radical HAT product from the C2 and C3OH sites in the **1B anion** with respect to **1B** can be explained also in this case by inspecting the electron spin density surfaces (Figure 2c–f). In both cases, a spontaneous proton transfer occurs from the OH of C2 to the carboxylate group, generating a carboxylic acid. This is characterized by a higher delocalization, which involves one or two C–O groups. In the case of C3OH, which becomes as reactive as any C site, the spin density is further delocalized on a C–C bond. Conversely, the destabilization of the radicals formed at sites C1 and C14 is caused by a lower delocalization.

For the **1B anion**, the SET mechanism was also considered for the scavenging activity of the five ROS. Gibbs free reaction energies ($\Delta G_{SET}^{o}$) computed in gas phase, in water and in benzene are reported in Table 2. These data show very endergonic reactivities via SET for each ROS in gas phase. The solvation in water and benzene reduces the endergonicity, particularly in the case of water, for the scavenging of ^•^OH. This is the only reaction that can be considered isergonic. Nevertheless, the thermodynamics of SET are more unfavorable compared to those of HAT from most of the available sites. Thus, the HAT mechanism can be considered the prevalent one.

The exergonicities of the HAT reactions in the cases of **1B** and the **1B anion** in scavenging the peroxyl radicals, although from a single site (C10), are remarkable results. Not even for Trolox, a well-known antioxidant (**3**, Scheme 1) [60], have such large negative Gibbs free energies for the scavenging of a peroxyl radical from a C site been reported [39]. Table S8 shows the data for Trolox computed at a consistent level of theory, i.e., (SMD)-M06-2X/6-311+G(d,p)//M06-2X/6-31G(d), by several of the present authors [39]. The most reactive site of Trolox is the OH group on the phenyl ring, which is characterized by the largest negative $\Delta G_{HAT}^{o}$ in any condition for the scavenging of any radical, e.g., for ^•^OOH $\Delta G_{HAT,water}^{o}$ = −9.3 kcal mol^−1^. However, the most reactive C site of Trolox (C6) is associated with a neatly positive $\Delta G_{HAT}^{o}$ value in the case of ^•^OOCH_3_. Conversely, the analogous HATs from C10 in **1B** and the **1B anion** are associated with negative $\Delta G_{HAT}^{o}$ values.

It is also possible to compare our results to the scavenging activity of melatonin via HAT, which is another compound with significant antioxidant activity [61,62]. For the two lowest energy conformers of melatonin, the $\Delta G_{HAT}^{o}$ values for the most reactive sites were computed at the same level of theory used in this work ((SMD)-M06-2X/6-311+G(d,p)//M06-2X/6-31G(d)) by several of the present authors [39]. Table S9 shows these values compared to the thermodynamic data on HAT from C10 on **1B** and the **1B anion**. Melatonin is a good scavenger for ^•^OH and ^•^OCH_3_; nevertheless, the $\Delta G_{HAT}^{o}$ values are always smaller (in absolute terms) than those computed for HAT from C10 on **1B** and the **1B anion**, e.g., for ^•^OCH_3_ $\Delta G_{HAT,water}^{o}$ = −18.7 kcal mol^−1^ for **4** against $\Delta G_{HAT,water}^{o}$ = −23.9 kcal mol^−1^ for **1B** from C10. Furthermore, melatonin is not an efficient scavenger of peroxyl radicals, while the selectivity of **1B** for these ROS via HAT from C10 is evident. Analogous conclusions are also valid for the other ginkgolides analyzed in this work (see the Supplementary Materials).

To rationalize the experimental results of Maitra et al. [17] which showed the scavenging activity of peroxyl radicals by *G. Biloba* extract, a comparison must also be made with the $\Delta G_{HAT}^{o}$ values of flavonoids, which usually account for roughly 20–30% of *G. Biloba* extracts [63,64]. On the other hand, terpenoids, including ginkgolides and bilobalide, generally represent roughly 5–10% of the components [64]. Castañeda-Arriaga et al. showed that, depending on their chemical scaffolds, flavonoids can either be bad (chrysin) or good (quercetin) scavengers of peroxyl radicals [65]. Specifically, when quercetin in its monoanionic form was considered, a favorable $\Delta G_{HAT}^{o}$ of −10 kcal mol^−1^ was computed for water, in which one of the phenolic OH functions of the molecule donates the hydrogen to ^•^OOH. The level of theory employed by Casta*ñ*eda-Arriaga et al. [65] is slightly different from the one employed in our work; thus, a rigorous comparison will not be attempted. However, these results show that both ginkgolides, from the C10 site, and at least some flavonoids, from the phenolic functions, can scavenge peroxyl radicals in *G. Biloba* extracts, supporting potential contributions from both classes of compounds to its experimentally tested radical scavenging activity.

### 3.3. HAT Kinetics of Ginkgolides

**Figure 4:**
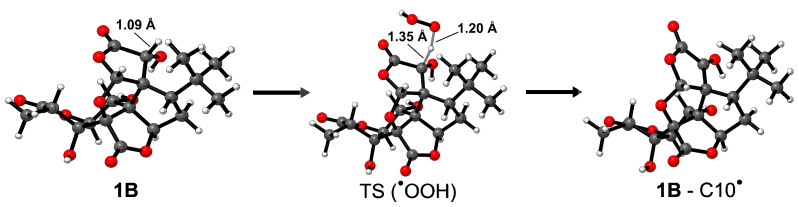
Fully optimized reactant, transition state and product (left to right) geometries of **1B** when HAT occurs from C10 for the scavenging of ^•^OOH. Relevant atomic distances are also reported in Å. Level of theory: M06-2X/6-31G(d).

To complete the investigation of the capacities of **1B** and the **1B anion** as radical scavengers, we computed the activation energies ($\Delta G_{HAT}^{‡}$) for HATs from the most reactive site, i.e., C10, in gas phase, in water and in benzene. For **1B**, $\Delta G_{HAT}^{‡}$ was also computed for one of the least favored HATs, i.e., the C10OH site. Table 3 shows the activation energies. The kinetic studies were limited to ginkgolide B, since the other ginkgolides were not expected to yield different results. Figure 4 shows the optimized structure of the transition state when HAT occurs from C10 on **1B** for the scavenging of ^•^OOH.

In gas phase as well as in solvent, $\Delta G_{HAT}^{‡}$ values for the two forms of ginkgolide B increase with the decreasing reactivities of the ROS. Hence, the trend of activation energies for the different ROS mirrors the thermodynamic trend of $\Delta G_{HAT}^{o}$: the lowest energy barriers correspond to the most exergonic reactions at a single site. Analogous considerations can be made by comparing the different sites of **1B**. HAT from the C10 site is characterized by smaller energy barriers than HAT from the C10OH site, regardless of the ROS or the environment. These kinetic differences are more remarkable for the peroxyl radicals, and they become less significant with ^•^OH. This is consistent with the reactivity–selectivity principle; hence, the most reactive ROS is less selective for a single site. Focusing on $\Delta G_{HAT}^{‡}$, for **1B** and the **1B anion** with respect to the C10 site, some considerations can be made. For all ROS, the energy barriers are always lower for the **1B anion** in gas phase. This is still consistent with the $\Delta G_{HAT}^{o}$ trend, since HAT from C10 is more favored for the **1B anion** in gas phase.

The presence of a solvent destabilizes the transition state with respect to the gas phase; this effect, which is found for each site and each ROS and is shown in Table 3, increases with increasing solvent polarity. In all of the considered cases, the energy barriers are slightly higher in benzene than in the gas phase, and they further increase in water. This effect is particularly remarkable for the **1B anion** in water, especially when comparing the C10 sites of the two forms of ginkgolide B in water. As previously stated, in water the $\Delta G_{HAT}^{o}$ differences between the neutral and the negative systems decrease, and this is reflected in the $\Delta G_{HAT}^{‡}\;$values. However, the effect on ^•^OH is particularly high, whereby the **1B anion** has a slightly higher barrier than **1B**.

### 3.4. HAT Energetics for Bilobalide

Finally, the scavenging potential of **2** via HAT was investigated. $\Delta G_{HAT}^{o}$ values in gas phase, water and benzene are reported for all sites in Figure S5 and Table S10. The chemical reactivity of **2** is very similar to those of the ginkgolides. The OH sites are the least reactive (negative $\Delta G_{HAT}^{o}$ only with ^•^OH), while C10 is the most reactive site. C10 of **2** has the same chemical topology as C10 in ginkgolides; it is bonded to a hydroxyl group and is in the α position with respect to a lactone carbonyl group. Furthermore, C10 is the only site characterized by negative $\Delta G_{HAT}^{o}$ values for peroxyl radicals. The similarities with ginkgolides also concern the energies in solvent. $\Delta G_{HAT}^{o}$ values observed for benzene show no remarkable differences to those observed for gas phase, while in water HAT reactions of **2** are more favorable, with differences of a few kcal mol^−1^.

**Scheme 3:**
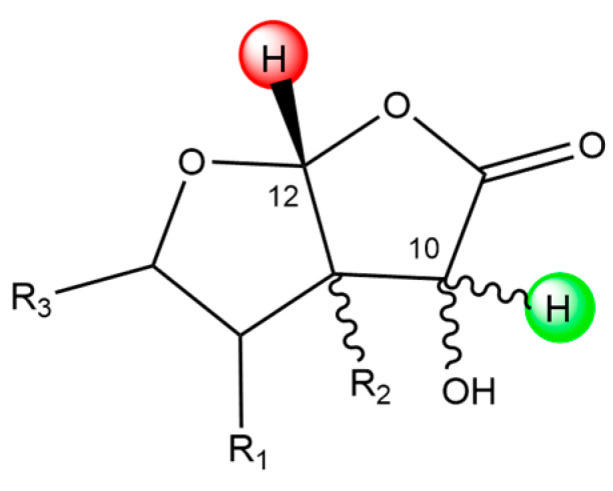
Representative chemical scaffold found in **1A–M**, the **1B anion** and **2**, including C10 and C12 sites. The green and red hydrogen atoms are particularly favored and unfavored C sites for HAT reactions, respectively.

We want to stress another parallelism between **2** and ginkgolides considering the C12 site. In both molecules, this site is bonded to two different oxygen atoms; in **2**, they both belong to a lactone group, while in ginkgolides only one oxygen belongs to a lactone group. C12 in each molecule is one of the least reactive sites, suggesting that this chemical position is not particularly favored for scavenging activity. Conversely, the chemical scaffold of C10 seems to be particularly favored for HAT reactions. Scheme 3 shows a representative chemical scaffold found in **1A–M**, the **1B anion** and **2**, focusing on the surroundings of the C10 and C12 sites.

## 4. Conclusions

Ginkgolides and bilobalide are naturally occurring polyhydroxylated compounds with unique chemical and pharmacological features. These molecules have been widely studied over the years, and experimental evidence shows that multiple mechanisms are involved in promoting their observed biological effects. Among these, direct and indirect antioxidant activities, as radical scavengers or mediated by interactions with cellular macromolecular targets, play a primary role.

In this study, with the aid of molecular modeling, we investigated the contributions of the different functional groups. We proved for the first time the direct involvement of C10 as a reactive site and the potential of completely saturated scaffolds in the scavenging of peroxyl radicals. More specifically, the latter site has the same chemical structure in ginkgolides and bilobalide, suggesting new possible features by means of which to improve the scavenging potential of chemical scaffolds. Furthermore, the C10 site displays selectivity for peroxyl radicals, unlike the C sites of Trolox and melatonin. In conclusion, this study led to the identification of peculiar topologies bearing highly reactive scavenging sites. The recognition that these moieties possess promising scavenging activities can improve the toolset for future studies in which similar structures will be inserted into model compounds to replicate their antioxidant activities in more readily available molecules. This increased knowledge certainly paves the way for the design and optimization of novel antioxidants.

In a more general context, the abovementioned findings support the crucial contributions of ginkgolides and bilobalide to the antioxidant properties of *G. biloba* extract. In fact, unlike flavonoids, which are well-known antioxidants that are present in several plants, ginkgolides and bilobalide represent peculiar components of *G. biloba* extract and contribute to its unique properties.

## Figures and Tables

**Table 1:** Gibbs free reaction energies ($\Delta G_{HAT}^{o}$ kcal mol^−1^) computed in the gas phase of **1B** and the **1B anion** for the scavenging of ^•^OH via HAT from the C1, C2, C10, C14 and C3OH sites. Level of theory: M06-2X/6-311+G(d,p)//M06-2X/6-31G(d).

Site	$\Delta G_{HAT}^{o}\;(1B)$	$\Delta G_{HAT}^{o}\;(1B\;\mathrm{anion})$	$\Delta \Delta G_{HAT}^{o}$
C1	−28.27	−20.16	8.11
C2	−16.94	−27.61	−10.67
C10	−34.38	−38.92	−4.54
C14	−26.55	−19.55	7.00
C3OH	−10.27	−23.41	−13.14

**Table 2:** Gibbs free reaction energies ($\Delta G_{SET}^{o}$ kcal mol^−1^) computed in gas phase, water and benzene for the **1B anion** for the scavenging of ^•^OH, ^•^OOH, ^•^OCH_3_, ^•^OOCH_3_ and ^•^OOCH=CH_2_ via SET. Level of theory: (SMD)-M06-2X/6-311+G(d,p)//M06-2X/6-31G(d).

ROS	$\Delta G_{SET}^{o}$	$\Delta G_{SET,\;water}^{o}$	$\Delta G_{SET,\;benzene}^{o}$
^•^OH	51.67	0.87	32.31
^•^OOH	65.85	23.10	50.13
^•^OCH_3_	56.18	20.18	41.55
^•^OOCH_3_	64.51	25.47	50.94
^•^OOCH=CH_2_	48.26	16.54	36.18

**Table 3:** Gibbs free activation energies ($\Delta G_{HAT}^{‡}$ kcal mol^−1^) computed in gas phase, water and benzene of **1B** and the **1B anion** for the scavenging of ^•^OH, ^•^OOH, ^•^OCH_3_, ^•^OOCH_3_ and ^•^OOCH=CH_2_ via HAT from C10 and C10OH. Level of theory: (SMD)-M06-2X/6-311+G(d,p)//M06-2X/6-31G(d).

Site	ROS	$\Delta G_{HAT}^{‡}$	$\Delta G_{HAT,\;water}^{‡}$	$\Delta G_{HAT,\;benzene}^{‡}$
	^•^OH	8.41	13.51	9.03
	^•^OOH	28.57	33.85	29.72
**1B-C10OH**	^•^OCH_3_	17.06	21.99	17.92
	^•^OOCH_3_	31.49	35.42	32.84
	^•^OOCH=CH_2_	28.28	31.20	29.16
	^•^OH	7.51	9.32	7.99
	^•^OOH	23.46	25.25	24.40
**1B-C10**	^•^OCH_3_	13.46	15.32	14.43
	^•^OOCH_3_	25.11	26.55	26.41
	^•^OOCH=CH_2_	24.33	23.58	24.91
	^•^OH	5.31	10.61	6.58
	^•^OOH	18.76	25.15	20.83
**1B anion-C10**	^•^OCH_3_	12.25	15.00	13.08
	^•^OOCH_3_	21.68	25.32	23.34
	^•^OOCH=CH_2_	20.05	23.79	21.59

## Data Availability

The data used are contained within the article and the Supplementary Materials.

## Supplementary Materials

The following supporting information can be downloaded at: https://www.mdpi.com/article/10.3390/antiox12020525/s1, Figures S1–S5: Gibbs free reaction energies ($\Delta G_{HAT}^{o}$) computed in gas phase, water and benzene for the scavenging of ·^•^OH, ^•^OOH, ^•^OCH_3_, ^•^OOCH_3_ and ^•^OOCH=CH_2_ via HAT from all the available sites of **1A, 1C, 1J, 1M,** and **2**. Level of theory: (SMD)-M06-2X/6-311+G(d,p)//M06-2X/6-31G(d); Tables S1–S6, S10: Gibbs free reaction energies ($\Delta G_{HAT}^{o}$) computed in gas phase, water and benzene for the scavenging of ^•^OH, ^•^OOH, ^•^OCH_3_, ^•^OOCH_3_ and ^•^OOCH=CH_2_ via HAT from all the available sites of **1A, 1B, 1C, 1J, 1M,** the **1B anion,** and **2**. Level of theory: (SMD)-M06-2X/6-311+G(d,p)//M06-2X/6-31G(d); Table S7: Gibbs free reaction energies ($\Delta G_{HAT}^{o}$ kcal mol^−1^) computed in water for **1B** and the **1B anion** for the scavenging of **·**OH via HAT from C1, C2, C14 and C3OH sites. Level of theory: M06-2X/6-311+G(d,p)//M06-2X/6-31G(d); Tables S8 and S9: Gibbs free reaction energies ($\Delta G_{HAT}^{o}$ kcal mol^−1^) computed in gas phase, water and benzene for the scavenging of ^•^OH, ^•^OOH, ^•^OCH_3_, ^•^OOCH_3_ and ^•^OOCH=CH_2_ via HAT from neutral Trolox (**3**, Scheme 1 in the text) and from the C4 sites of the two conformers of melatonin (**4**, Scheme 1 in the text). For comparison, the HAT values for C10 for **1B** and the **1B anion** are also included. Level of theory: (SMD)-M06-2X/6-311+G(d,p)//M06-2X/6-31G(d); Table S11: Coordinates of the structures of **1A–M**, the **1B anion**, and **2** and their radicals at each available site. Level of theory: M06-2X/6-31G(d); Tables S12–S14: Coordinates and imaginary frequencies of the transition states of the HAT from the C10 and C10OH sites of **1B** and from C10 of the **1B anion** to each ROS. Level of theory: M06-2X/6-31G(d); Table S15: Coordinates of the structures of the five ROS, their anion and protonated forms. Level of theory: M06-2X/6-31G(d).

## References

[1] Villamena F.A. (2013). Molecular Basis of Oxidative Stress: Chemistry, Mechanisms, and Disease Pathogenesis.

[2] Neha K., Haider M.R., Pathak A., Yar M.S. (2019). Medicinal Prospects of Antioxidants: A Review. Eur. J. Med. Chem..

[3] Sharifi-Rad M., Anil Kumar N.V., Zucca P., Varoni E.M., Dini L., Panzarini E., Rajkovic J., Tsouh Fokou P.V., Azzini E., Peluso I. (2020). Lifestyle, Oxidative Stress, and Antioxidants: Back and Forth in the Pathophysiology of Chronic Diseases. Front. Physiol..

[4] Sies H. (2015). Oxidative Stress: A Concept in Redox Biology and Medicine. Redox. Biol..

[5] Barnham K.J., Masters C.L., Bush A.I. (2004). Neurodegenerative Diseases and Oxidative Stress. Nat. Rev. Drug Discov..

[6] Dubois-deruy E., Peugnet V., Turkieh A., Pinet F. (2020). Oxidative Stress in Cardiovascular Diseases. Antioxidants.

[7] Klaunig J.E. (2019). Oxidative Stress and Cancer. Curr. Pharm. Des..

[8] Tobe E.M.D. (2013). Oxidative Stress, and Major Depressive Disorder. Neuropsychiatr. Dis. Treat..

[9] Emiliani F.E., Sedlak T.W., Sawa A. (2014). Oxidative Stress and Schizophrenia: Recent Breakthroughs from an Old Story. Curr. Opin. Psychiatry.

[10] Eleutherio E.C.A., Silva Magalhães R.S., Araújo Brasil A., Monteiro Neto J.R., Holanda Paranhos L.S. (2021). More than Just an Antioxidant. Arch. Biochem. Biophys..

[11] Deisseroth A., Dounce A.L. (1970). Catalase: Physical and Chemical Properties, Mechanism of Catalysis, and Physiological Role. Physiol. Rev..

[12] Flohé L., Toppo S., Orian L. (2022). The Glutathione Peroxidase Family: Discoveries and Mechanism. Free Radic. Biol. Med..

[13] Orian L., Flohé L. (2021). Selenium-Catalyzed Reduction of Hydroperoxides in Chemistry and Biology. Antioxidants.

[14] Narayanankutty A., Job J.T., Narayanankutty V. (2019). Glutathione, an Antioxidant Tripeptide: Dual Roles in Carcinogenesis and Chemoprevention. Curr. Protein. Pept. Sci..

[15] Reiter R.J., Mayo J.C., Tan D.-X., Sainz R.M., Alatorre-Jimenez M., Qin L. (2016). Melatonin as an Antioxidant: Under Promises but over Delivers. J. Pineal Res..

[16] Rahaman M., Hossain R., Herrera-Bravo J., Islam M.T., Atolani O., Adeyemi O.S., Owolodun O.A., Kambizi L., Daştan S.D., Calina D. (2023). Natural Antioxidants from Some Fruits, Seeds, Foods, Natural Products, and Associated Health Benefits: An Update. Food Sci. Nutr..

[17] Maitra I., Marcocci L., Droy-Lefaix M.T., Packer L. (1995). Peroxyl Radical Scavenging Activity of Ginkgo Biloba Extract EGb 761. Biochem. Pharmacol..

[18] Crimmins M.T., Pace J.M., Nantermet P.G., Kim-Meade A.S., Thomas J.B., Watterson S.H., Wagman A.S. (2000). The Total Synthesis of (±)-Ginkgolide B. J. Am. Chem. Soc..

[19] Beek T.A. (2005). Ginkgolides and Bilobalide: Their Physical, Chromatographic and Spectroscopic Properties. Bioorg. Med. Chem..

[20] Sarkar C., Quispe C., Jamaddar S., Hossain R., Ray P., Mondal M., Abdulwanis Mohamed Z., Sani Jaafaru M., Salehi B., Islam M.T. (2020). Therapeutic Promises of Ginkgolide A: A Literature-Based Review. Biomed. Pharmacother..

[21] Souza G., Marqui S.V., Matias J.N., Guiguer E.L., Barbalho S.M. (2020). Effects of Ginkgo Biloba on Diseases Related to Oxidative Stress. Planta Med..

[22] Feng Z., Sun Q., Chen W., Bai Y., Hu D., Xie X. (2019). The Neuroprotective Mechanisms of Ginkgolides and Bilobalide in Cerebral Ischemic Injury: A Literature Review. Mol. Med..

[23] Gachowska M., Szlasa W., Saczko J., Kulbacka J. (2021). Neuroregulatory Role of Ginkgolides. Mol. Biol. Rep..

[24] Lu J., Xie L., Liu K., Zhang X., Wang X., Dai X., Liang Y., Cao Y., Li X. (2021). Bilobalide: A Review of Its Pharmacology, Pharmacokinetics, Toxicity, and Safety. Phytother. Res..

[25] Su Q., Dong J., Zhang D., Yang L., Roy R. (2022). Protective Effects of the Bilobalide on Retinal Oxidative Stress and Inflammation in Streptozotocin-Induced Diabetic Rats. Appl. Biochem. Biotechnol..

[26] Liu Q., Jin Z., Xu Z., Yang H., Li L., Li G., Li F., Gu S., Zong S., Zhou J. (2019). Antioxidant Effects of Ginkgolides and Bilobalide against Cerebral Ischemia Injury by Activating the Akt/Nrf2 Pathway in Vitro and in Vivo. Cell Stress Chaperones.

[27] Lu L., Wang S., Fu L., Liu D., Zhu Y., Xu A. (2016). Bilobalide Protection of Normal Human Melanocytes from Hydrogen Peroxide-Induced Oxidative Damage via Promotion of Antioxidase Expression and Inhibition of Endoplasmic Reticulum Stress. Clin. Exp. Dermatol..

[28] Chandrasekaran K., Mehrabian Z., Spinnewyn B., Chinopoulos C., Drieu K., Fiskum G. (2002). Bilobalide, a Component of the Ginkgo Biloba Extract (EGb 761), Protects against Neuronal Death in Global Brain Ischemia and in Glutamate-Induced Excitotoxicity. Cell Mol. Biol..

[29] Guo R.Z., Liu X.G., Gao W., Dong X., Fanali S., Li P., Yang H. (2015). A Strategy for Screening Antioxidants in Ginkgo Biloba Extract by Comprehensive Two-Dimensional Ultra High Performance Liquid Chromatography. J. Chromatogr. A.

[30] Pietta P.-G. (2000). Flavonoids as Antioxidants. J. Nat. Prod..

[31] Bors W., Saran M. (1987). Radical Scavenging by Flavonoid Antioxidants. Free Radic. Res. Commun..

[32] Scholtyssek H., Damerau W., Wessel R., Schimke I. (1997). Antioxidative Activity of Ginkgolides against Superoxide in an Aprotic Environment. Chem. Biol. Interact..

[33] Zekri O., Boudeville P., Genay P., Perly B., Braquet P., Jouenne P., Burgot J.-L. (1996). Ionization Constants of Ginkgolide B in Aqueous Solution. Anal. Chem..

[34] Wang D.-L., Peng D.-Y., Tao X.-H., Cao Y., Chen W.-D., Liang Y., Xie L., Liu X.-D. (2013). The Pharmacokinetics and Conversion of the Lactone to the Carboxylate Forms of Ginkgolide B in Rat Plasma. J. Asian Nat. Prod. Res..

[35] Suehiro M., Simpson N.R., Underwood M.D., Castrillon J., Nakanishi K., Heertum R. (2005). In Vivo Biodistribution of Ginkgolide B, a Constituent of Ginkgo Biloba, Visualized by MicroPET. Planta Med..

[36] Nakanishi K., Habaguchi K., Nakadaira Y., Woods M.C., Maruyama M., Major R.T., Alauddin M., Patel A.R., Weinges K., Baehr W. (1971). Structure of Bilobalide, a Rare Tert-Butyl Containing Sesquiterpenoid Related to the C20-Ginkgolides. J. Am. Chem. Soc..

[37] Biber A., Koch E. (1999). Bioavailability of Ginkgolides and Bilobalide from Extracts of Ginkgo Biloba Using GC/MS. Planta Med..

[38] Galano A., Mazzone G., Alvarez-Diduk R., Marino T., Alvarez-Idaboy J.R., Russo N. (2016). Food Antioxidants: Chemical Insights at the Molecular Level. Annu. Rev. Food Sci. Technol..

[39] Bortoli M., Dalla Tiezza M., Muraro C., Pavan C., Ribaudo G., Rodighiero A., Tubaro C., Zagotto G., Orian L. (2019). Psychiatric Disorders and Oxidative Injury: Antioxidant Effects of Zolpidem Therapy Disclosed In Silico. Comput. Struct. Biotechnol. J..

[40] Muraro C., Dalla Tiezza M., Pavan C., Ribaudo G., Zagotto G., Orian L. (2019). Major Depressive Disorder and Oxidative Stress: In Silico Investigation of Fluoxetine Activity against ROS. Appl. Sci..

[41] Dalla Tiezza M., Hamlin T.A., Bickelhaupt F.M., Orian L. (2021). Radical Scavenging Potential of the Phenothiazine Scaffold A Computational Analysis. ChemMedChem.

[42] Ribaudo G., Bortoli M., Witt C.E., Parke B., Mena S., Oselladore E., Zagotto G., Hashemi P., Orian L. (2022). ROS-Scavenging Selenofluoxetine Derivatives Inhibit In Vivo Serotonin Reuptake. ACS Omega.

[43] Ribaudo G., Bortoli M., Ongaro A., Oselladore E., Gianoncelli A., Zagotto G., Orian L. (2020). Fluoxetine Scaffold to Design Tandem Molecular Antioxidants and Green Catalysts. RSC Adv..

[44] Ribaudo G., Bortoli M., Pavan C., Zagotto G., Orian L. (2020). Antioxidant Potential of Psychotropic Drugs: From Clinical Evidence to In Vitro and In Vivo Assessment and toward a New Challenge for in Silico Molecular Design. Antioxidants.

[45] Galano A., Raúl Alvarez-Idaboy J. (2019). Computational Strategies for Predicting Free Radical Scavengers’ Protection against Oxidative Stress: Where Are We and What Might Follow?. Int. J. Quantum. Chem..

[46] Muraro C., Polato M., Bortoli M., Aiolli F., Orian L. (2020). Radical Scavenging Activity of Natural Antioxidants and Drugs: Development of a Combined Machine Learning and Quantum Chemistry Protocol. J. Chem. Phys..

[47] Zhao Y., Truhlar D.G. (2008). The M06 Suite of Density Functionals for Main Group Thermochemistry, Thermochemical Kinetics, Noncovalent Interactions, Excited States, and Transition Elements: Two New Functionals and Systematic Testing of Four M06-Class Functionals and 12 Other Functionals. Theor. Chem. Account..

[48] Frisch M.J., Trucks G.W., Schlegel H.B., Scuseria G.E., Robb M.A., Cheeseman J.R., Scalmani G., Barone V., Petersson G.A., Nakatsuji H. Gaussian 16 Rev. C.01 2016.

[49] Marenich A.V., Cramer C.J., Truhlar D.G. (2009). Universal Solvation Model Based on Solute Electron Density and on a Continuum Model of the Solvent Defined by the Bulk Dielectric Constant and Atomic Surface Tensions. J. Phys. Chem. B.

[50] Spiegel M. (2022). Current Trends in Computational Quantum Chemistry Studies on Antioxidant Radical Scavenging Activity. J. Chem. Inf. Model.

[51] Jensen J.H. (2015). Predicting Accurate Absolute Binding Energies in Aqueous Solution: Thermodynamic Considerations for Electronic Structure Methods. Phys. Chem. Chem. Phys..

[52] Galano A., Álvarez-Diduk R., Ramírez-Silva M.T., Alarcón-Ángeles G., Rojas-Hernández A. (2009). Role of the Reacting Free Radicals on the Antioxidant Mechanism of Curcumin. Chem. Phys..

[53] Hanwell M.D., Curtis D.E., Lonie D.C., Vandermeerschd T., Zurek E., Hutchison G.R. (2012). Avogadro: An Advanced Semantic Chemical Editor, Visualization, and Analysis Platform. J. Cheminform..

[54] Droy-Lefaix M.T. (1997). Effect of the Antioxidant Action of Ginkgo Biloba Extract (EGb 761) on Aging and Oxidative Stress. Age (Omaha).

[55] Liu C., Cheng Y., Hu J., Zhang W., Chen N., Zhang J. (2006). Comparison of Antioxidant Activities between Salvianolic Acid B and Ginkgo Biloba Extract (EGb 761). Acta Pharmacol. Sin..

[56] Singh S.K., Srivastav S., Castellani R.J., Plascencia-Villa G., Perry G. (2019). Neuroprotective and Antioxidant Effect of Ginkgo Biloba Extract Against AD and Other Neurological Disorders. Neurotherapeutics.

[57] Di Meo F., Cuciniello R., Margarucci S., Bergamo P., Petillo O., Peluso G., Filosa S., Crispi S. (2020). Ginkgo Biloba Prevents Oxidative Stress-Induced Apoptosis Blocking P53 Activation in Neuroblastoma Cells. Antioxidants.

[58] Draganić I.G., Draganić Z.D. (1971). The Radiation Chemistry of Water.

[59] Pryor W.A. (1986). Oxy-Radicals and Related Species: Their Formation, Lifetimes, and Reactions. Annu. Rev. Physiol..

[60] Alberto M.E., Russo N., Grand A., Galano A. (2013). A Physicochemical Examination of the Free Radical Scavenging Activity of Trolox: Mechanism, Kinetics and Influence of the Environment. Phys. Chem. Chem. Phys..

[61] Galano A., Medina M.E., Tan D.X., Reiter R.J. (2015). Melatonin and Its Metabolites as Copper Chelating Agents and Their Role in Inhibiting Oxidative Stress: A Physicochemical Analysis. J. Pineal Res..

[62] Reiter R.J., Rosales-Corral S., Tan D.X., Jou M.J., Galano A., Xu B. (2017). Melatonin as a Mitochondria-Targeted Antioxidant: One of Evolution’s Best Ideas. Cell Mol. Life Sci..

[63] Zhang Z., Chen S., Mei H., Xuan J., Guo X., Couch L., Dobrovolsky V.N., Guo L., Mei N. (2015). Ginkgo Biloba Leaf Extract Induces DNA Damage by Inhibiting Topoisomerase II Activity in Human Hepatic Cells. Sci. Rep..

[64] Ding S., Dudley E., Plummer S., Tang J., Newton R.P., Brenton A.G. (2006). Quantitative Determination of Major Active Components in Ginkgo Biloba Dietary Supplements by Liquid Chromatography/Mass Spectrometry: Quantitation of Components in Ginkgo Biloba Extract by LC/MS. Rapid Commun. Mass Spectrom..

[65] Castañeda-Arriaga R., Marino T., Russo N., Alvarez-Idaboy J.R., Galano A. (2020). Chalcogen Effects on the Primary Antioxidant Activity of Chrysin and Quercetin. New J. Chem..

