# Supplementary material for: Radical Scavenging Potential of Ginkgolides and Bilobalide: Insight from Molecular Modeling
Source: Antioxidants (Basel). 2023 Feb 19;12(2):525. doi: 10.3390/antiox12020525 (PMC9952587; doi:10.3390/antiox12020525)
Supplement: Supplementary file 1 [file antioxidants-12-00525-s001.zip › antioxidants-2211197-supplementary.pdf]

# - SUPPORTING INFORMATION -

Radical scavenging potential of ginkgolides and bilobalide: insight from molecular modeling

Davide Zeppilli [1,§], Giovanni Ribaudo [2,§], Nicola Pompermaier [1], Andrea Madabeni [1], Marco Bortoli [3] and Laura Orian [1,*]

[1] Dipartimento di Scienze Chimiche Università degli Studi di Padova Via Marzolo 1 35129 Padova, Italy

[2] Dipartimento di Medicina Molecolare e Traslazionale, Università degli Studi di Brescia, Viale Europa 11, 25123 Brescia, Italy

[3] Department of Chemistry and Hylleraas Centre for Quantum Molecular Sciences, University of Oslo, Oslo 0315, Norway

* Correspondence: laura.orian@unipd.it (L.O.)

§ These authors contributed equally

LIST of TABLES and FIGURES:

Table S1 Gibbs free reaction energies (ΔG^{o}_{HAT}) computed in gas-phase, water and benzene for the scavenging of •OH, •OOH, •OCH_3, •$OOCH_3$ and •$OOCH{=}CH_2$ via HAT from all the available sites of **1B**. Level of theory: (SMD)-M06-2X/6-311+G(d,p)//M06-2X/6-31G(d)……………………………………………………3

Figure S1 Gibbs free reaction energies (ΔG^{o}_{HAT}) computed in (a) gas-phase, (b) water and (c) benzene for the scavenging of •OH, •OOH, •OCH_3, •$OOCH_3$ and •$OOCH{=}CH_2$ via HAT from all the available sites of **1A**. Level of theory: (SMD)-M06-2X/6-311+G(d,p)//M06-2X/6-31G(d)……………………………………………………4

Table S2 Gibbs free reaction energies (ΔG^{o}_{HAT}) computed in gas-phase, water and benzene for the scavenging of •OH, •OOH, •OCH_3, •$OOCH_3$ and •$OOCH{=}CH_2$ via HAT from all the available sites of **1A**. Level of theory: (SMD)-M06-2X/6-311+G(d,p)//M06-2X/6-31G(d)……………………………………………………4

Figure S2 Gibbs free reaction energies (ΔG^{o}_{HAT}) computed in (a) gas-phase, (b) water and (c) benzene for the scavenging of •OH, •OOH, •OCH_3, •$OOCH_3$ and •$OOCH{=}CH_2$ via HAT from all the available sites of **1C**. Level of theory: (SMD)-M06-2X/6-311+G(d,p)//M06-2X/6-31G(d)……………………………………………………5

Table S3 Gibbs free reaction energies (ΔG^{o}_{HAT}) computed in gas-phase, water and benzene for the scavenging of •OH, •OOH, •OCH_3, •$OOCH_3$ and •$OOCH{=}CH_2$ via HAT from all the available sites of **1C**. Level of theory: (SMD)-M06-2X/6-311+G(d,p)//M06-2X/6-31G(d)……………………………………………………5

Figure S3 Gibbs free reaction energies (ΔG^{o}_{HAT}) computed in (a) gas-phase, (b) water and (c) benzene for the scavenging of •OH, •OOH, •OCH_3, •$OOCH_3$ and •$OOCH{=}CH_2$ via HAT from all the available sites of **1J**. Level of theory: (SMD)-M06-2X/6-311+G(d,p)//M06-2X/6-31G(d)……………………………………………………6

Table S4 Gibbs free reaction energies (ΔG^{o}_{HAT}) computed in gas-phase, water and benzene for the scavenging of •OH, •OOH, •OCH_3, •$OOCH_3$ and •$OOCH{=}CH_2$ via HAT from all the available sites of **1J**. Level of theory: (SMD)-M06-2X/6-311+G(d,p)//M06-2X/6-31G(d)……………………………………………………6

Figure S4 Gibbs free reaction energies (ΔG^{o}_{HAT}) computed in (a) gas-phase, (b) water and (c) benzene for the scavenging of •OH, •OOH, •OCH_3, •$OOCH_3$ and •OOCH=CH_2 via HAT from all the available sites of **1M**. Level of theory: (SMD)-M06-2X/6-311+G(d,p)//M06-2X/6-31G(d)……7

Table S5 Gibbs free reaction energies (ΔG^{o}_{HAT}) computed in gas-phase, water and benzene for the scavenging of •OH, •OOH, •OCH_3, •$OOCH_3$ and •OOCH=CH_2 via HAT from all the available sites of **1M**. Level of theory: (SMD)-M06-2X/6-311+G(d,p)//M06-2X/6-31G(d)……7

Table S6 Gibbs free reaction energies (ΔG^{o}_{HAT}) computed in gas-phase, water and benzene for the scavenging of •OH, •OOH, •OCH_3, •$OOCH_3$ and •OOCH=CH_2 via HAT from all the available sites of **1B**$^-$. Level of theory: (SMD)-M06-2X/6-311+G(d,p)//M06-2X/6-31G(d)……8

Table S7 Gibbs free reaction energies (ΔG^{o}_{HAT} kcal mol^{-1}) computed in water of **1B** and **1B**$^-$ for the scavenging of ·OH via HAT from C1, C2, C14 and C3OH sites. Level of theory: M06-2X/6-311+G(d,p)//M06-2X/6-31G(d)……8

Table S8 Gibbs free reaction energies (ΔG^{o}_{HAT} kcal mol^{-1}) computed in gas-phase, water and benzene, for the scavenging of •OH, •OOH, •OCH_3, •$OOCH_3$ and •OOCH=CH_2 via HAT from neutral Trolox[a] (**3**, Scheme 1 in the text). For comparison the values of HAT from C10 of **1B** and **1B**$^-$ are also included. Level of theory: (SMD)-M06-2X/6-311+G(d,p)//M06-2X/6-31G(d)……9

Table S9 Gibbs free reaction energies (ΔG^{o}_{HAT} kcal mol^{-1}) computed in gas-phase, water and benzene, for the scavenging of •OH, •OOH, •OCH_3, •$OOCH_3$ and •OOCH=CH_2 via HAT from C4 site of melatonin[a] (**4**, Scheme 1 in the text). Two lowest energy conformers of melatonin have been considered[b]. For comparison the values of HAT from C10 of **1B** and **1B**$^-$ are also included. Level of theory: (SMD)-M06-2X/6-311+G(d,p)//M06-2X/6-31G(d)……9

Figure S5 Gibbs free reaction energies (ΔG^{o}_{HAT}) computed in (a) gas-phase, (b) water and (c) benzene for the scavenging of •OH, •OOH, •OCH_3, •$OOCH_3$ and •OOCH=CH_2 via HAT from all the available sites of **2**. Level of theory: (SMD)-M06-2X/6-311+G(d,p)//M06-2X/6-31G(d)……10

Table S10 Gibbs free reaction energies (ΔG^{o}_{HAT}) computed in gas-phase, water and benzene for the scavenging of ·•OH, •OOH, •OCH_3, •$OOCH_3$ and •OOCH=CH_2 via HAT from all the available sites of **2**. Level of theory: (SMD)-M06-2X/6-311+G(d,p)//M06-2X/6-31G(d)……10

Table S11 Coordinates of the structures of **1A-M**, **1B**$^-$, **2** and their radicals at each available site. Level of theory: M06-2X/6-31G(d)……11

Table S12 Coordinates and imaginary frequencies of the transition state of the HAT from C10 site of **1B** to each ROS. Level of theory: M06-2X/6-31G(d)……45

Table S13 Coordinates and imaginary frequencies of the transition state of the HAT from C10OH site of **1B** to each ROS. Level of theory: M06-2X/6-31G(d)……47

Table S14 Coordinates and imaginary frequencies of the transition state of the HAT from C10 site of **1B**$^-$ to each ROS. Level of theory: M06-2X/6-31G(d)……49

Table S15 Coordinates of the structures of the five ROSs, their anion and protonated forms. Level of theory: M06-2X/6-31G(d)……51

Table S1 Gibbs free reaction energies (ΔG^{o}_{HAT}) computed in gas-phase, water and benzene for the scavenging of •OH, •OOH, •OCH_3, •$OOCH_3$ and •OOCH=CH_2 via HAT from all the available sites of **1B**. Level of theory: (SMD)-M06-2X/6-311+G(d,p)//M06-2X/6-31G(d).

	ΔG^{o}_{HAT}					$\Delta G^{o}_{HAT,water}$					$\Delta G^{o}_{HAT,benzene}$				
	•OH	•OOH	•OCH_3	•$OOCH_3$	•OOCH=CH_2	•OH	•OOH	•OCH_3	•$OOCH_3$	•OOCH=CH_2	•OH	•OOH	•OCH_3	•$OOCH_3$	•OOCH=CH_2
**C1**	-28.27	3.72	-13.37	5.32	2.43	-28.64	4.24	-13.14	5.51	1.96	-28.41	4.03	-12.94	5.90	2.56
**C2**	-16.94	15.05	-2.04	16.65	13.76	-17.70	15.17	-2.20	16.44	12.89	-16.96	15.48	-1.49	17.35	14.01
**C6**	-15.41	16.58	-0.51	18.17	15.29	-17.66	15.21	-2.16	16.48	12.93	-15.56	16.88	-0.09	18.75	15.41
**C7**	-18.73	13.27	-3.82	14.86	11.97	-22.38	10.50	-6.88	11.77	8.22	-19.48	12.97	-4.01	14.83	11.50
**C8**	-24.95	7.04	-10.05	8.63	5.74	-27.82	5.05	-12.33	6.32	2.77	-25.70	6.74	-10.24	8.61	5.27
**C8tBu**	-17.83	14.16	-2.93	15.76	12.87	-20.59	12.29	-5.09	13.56	10.00	-18.13	14.31	-2.66	16.18	12.84
**C10**	-34.38	-2.39	-19.48	-0.79	-3.68	-39.35	-6.47	-23.85	-5.20	-8.75	-35.92	-3.48	-20.45	-1.61	-4.95
**C12**	-17.24	14.76	-2.34	16.35	13.46	-17.91	14.97	-2.40	16.24	12.69	-16.96	15.49	-1.49	17.35	14.01
**C14**	-26.55	5.45	-11.64	7.04	4.15	-29.59	3.29	-14.09	4.55	1.00	-26.98	5.47	-11.51	7.33	3.99
**C14Me**	-17.40	14.59	-2.50	16.18	13.30	-20.31	12.56	-4.81	13.83	10.28	-17.74	14.71	-2.27	16.57	13.24
**C1OH**	-10.70	21.30	4.20	22.89	20.00	-12.68	20.20	2.82	21.47	17.92	-11.80	20.64	3.67	22.51	19.17
**C3OH**	-11.48	20.51	3.42	22.10	19.22	-11.73	21.15	3.77	22.42	18.86	-11.70	20.74	3.77	22.61	19.27
**C10OH**	-10.27	21.72	4.63	23.32	20.43	-12.49	20.39	3.02	21.66	18.11	-11.28	21.16	4.19	23.03	19.69

Figure S1 Gibbs free reaction energies (ΔG^{o}_{HAT}) computed in (a) gas-phase, (b) water and (c) benzene for the scavenging of •OH, •OOH, •OCH_3, •$OOCH_3$ and •OOCH=CH_2 via HAT from all the available sites of **1A**. Level of theory: (SMD)-M06-2X/6-311+G(d,p)//M06-2X/6-31G(d).

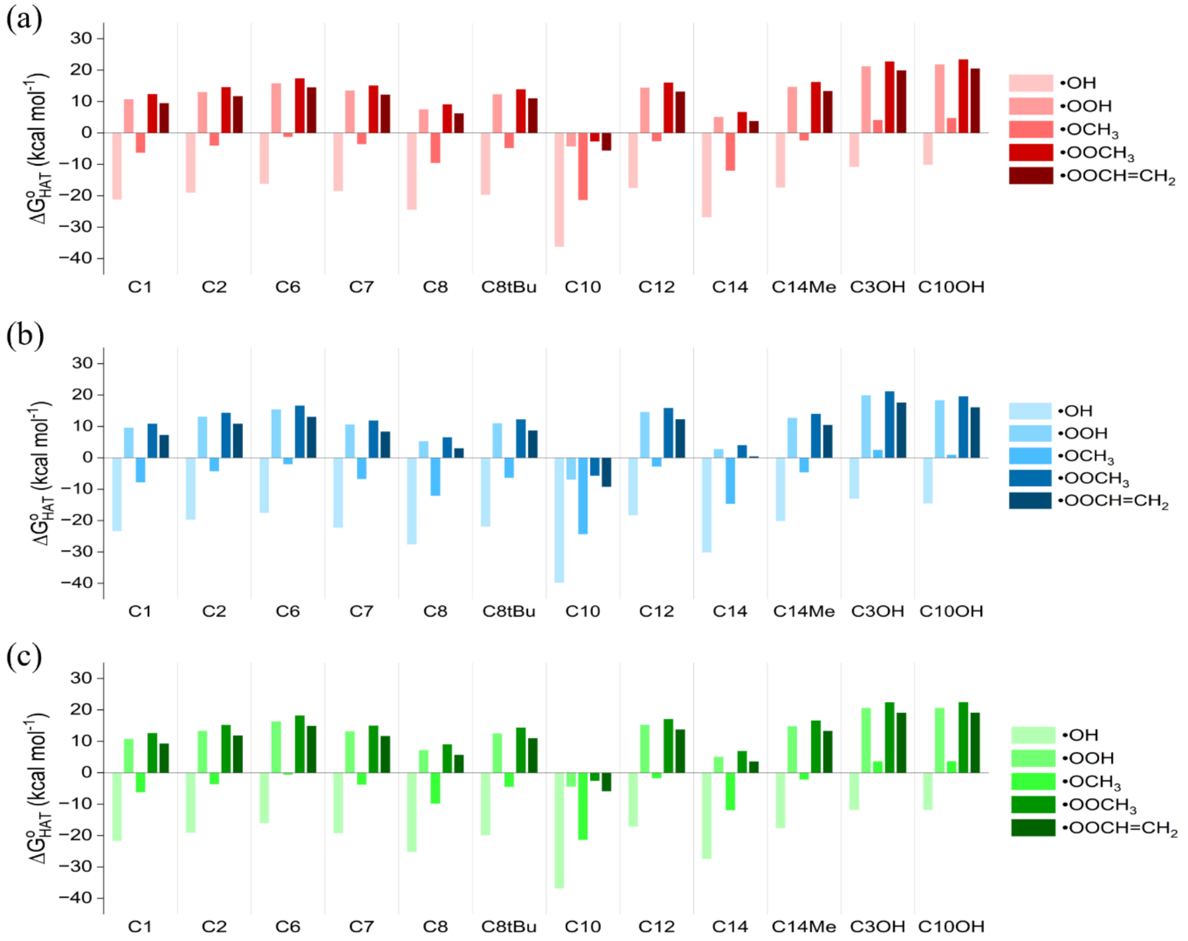

Table S2 Gibbs free reaction energies (ΔG^{o}_{HAT}) computed in gas-phase, water and benzene for the scavenging of •OH, •OOH, •OCH_3, •$OOCH_3$ and •OOCH=CH_2 via HAT from all the available sites of **1A**. Level of theory: (SMD)-M06-2X/6-311+G(d,p)//M06-2X/6-31G(d).

	ΔG^{o}_{HAT}					$\Delta G^{o}_{HAT,water}$					$\Delta G^{o}_{HAT,benzene}$				
	•OH	•OOH	•OCH_3	•$OOCH_3$	•OOCH=CH_2	•OH	•OOH	•OCH_3	•$OOCH_3$	•OOCH=CH_2	•OH	•OOH	•OCH_3	•$OOCH_3$	•OOCH=CH_2
**C1**	-21.21	10.79	-6.31	12.38	9.49	-23.33	9.55	-7.83	10.82	7.27	-21.68	10.77	-6.21	12.63	9.29
**C2**	-18.99	13.01	-4.09	14.60	11.71	-19.78	13.10	-4.28	14.36	10.81	-19.10	13.34	-3.63	15.21	11.87
**C6**	-16.18	15.81	-1.28	17.41	14.52	-17.54	15.34	-2.03	16.61	13.06	-16.09	16.35	-0.62	18.22	14.88
**C7**	-18.49	13.50	-3.59	15.09	12.20	-22.26	10.62	-6.76	11.88	8.33	-19.28	13.16	-3.81	15.03	11.69
**C8**	-24.45	7.54	-9.55	9.13	6.25	-27.60	5.28	-12.10	6.55	3.00	-25.25	7.19	-9.78	9.06	5.72
**C8tBu**	-19.70	12.30	-4.80	13.89	11.00	-21.88	11.00	-6.38	12.27	8.71	-19.94	12.50	-4.48	14.37	11.03
**C10**	-36.29	-4.29	-21.38	-2.70	-5.59	-39.83	-6.96	-24.33	-5.69	-9.24	-36.88	-4.44	-21.41	-2.57	-5.91
**C12**	-17.52	14.47	-2.62	16.07	13.18	-18.30	14.58	-2.80	15.85	12.29	-17.19	15.25	-1.72	17.12	13.78
**C14**	-26.89	5.10	-11.99	6.69	3.80	-30.13	2.75	-14.63	4.02	0.46	-27.38	5.06	-11.91	6.93	3.59
**C14Me**	-17.35	14.65	-2.44	16.24	13.35	-20.16	12.72	-4.66	13.99	10.43	-17.63	14.81	-2.16	16.68	13.34
**C3OH**	-10.80	21.20	4.11	22.79	19.90	-12.98	19.89	2.52	21.16	17.61	-11.84	20.60	3.63	22.47	19.13
**C10OH**	-10.15	21.85	4.76	23.44	20.55	-14.54	18.34	0.96	19.61	16.06	-11.81	20.63	3.65	22.50	19.16

Figure S2 Gibbs free reaction energies (ΔG^{o}_{HAT}) computed in (a) gas-phase, (b) water and (c) benzene for the scavenging of $^{\bullet}OH$, $^{\bullet}OOH$, $^{\bullet}OCH_3$, $^{\bullet}OOCH_3$ and $^{\bullet}OOCH{=}CH_2$ via HAT from all the available sites of **1C**. Level of theory: (SMD)-M06-2X/6-311+G(d,p)//M06-2X/6-31G(d).

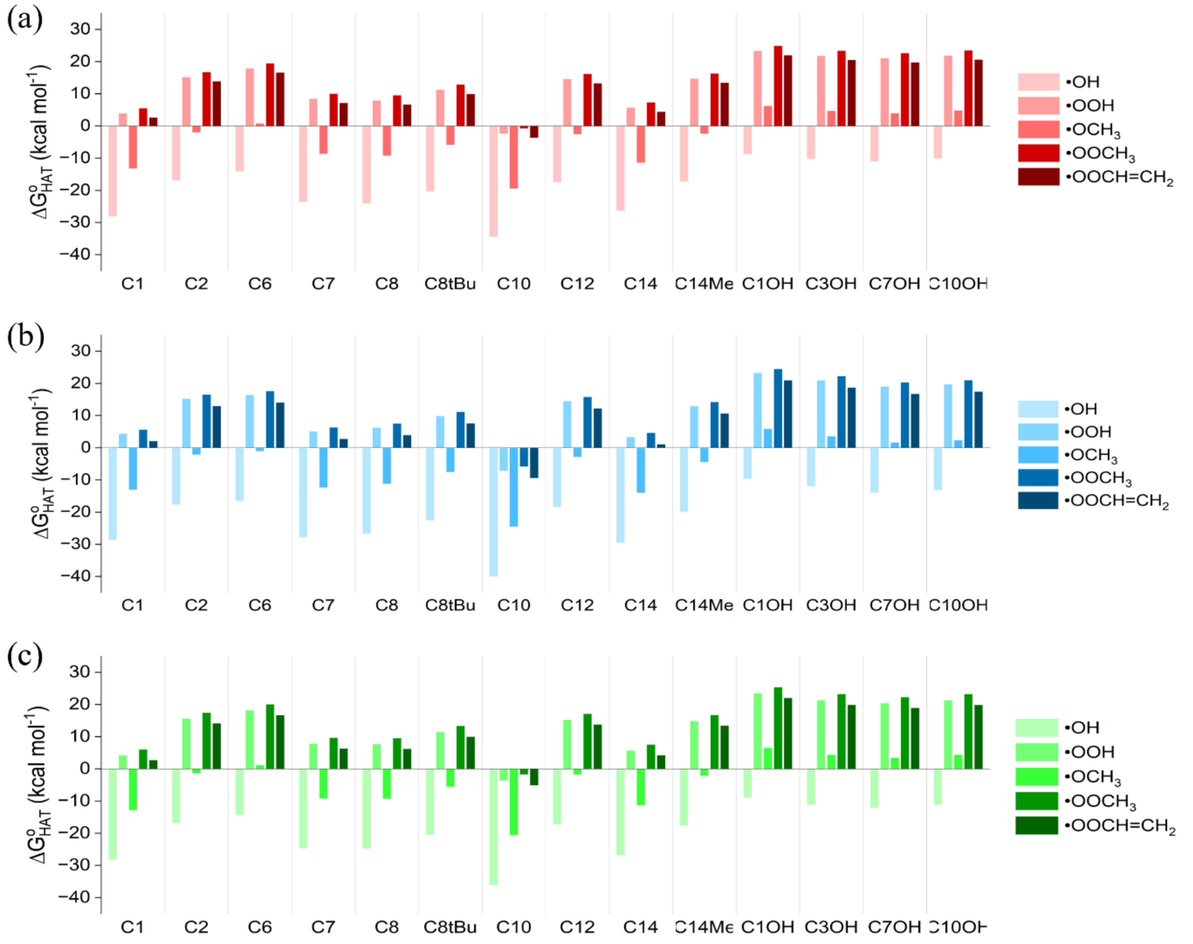

Table S3 Gibbs free reaction energies (ΔG^{o}_{HAT}) computed in gas-phase, water and benzene for the scavenging of $^{\bullet}OH$, $^{\bullet}OOH$, $^{\bullet}OCH_3$, $^{\bullet}OOCH_3$ and $^{\bullet}OOCH{=}CH_2$ via HAT from all the available sites of **1C**. Level of theory: (SMD)-M06-2X/6-311+G(d,p)//M06-2X/6-31G(d).

	ΔG^{o}_{HAT}					$\Delta G^{o}_{HAT,water}$					$\Delta G^{o}_{HAT,benzene}$				
	$^{\bullet}OH$	$^{\bullet}OOH$	$^{\bullet}OCH_3$	$^{\bullet}OOCH_3$	$^{\bullet}OOCH{=}CH_2$	$^{\bullet}OH$	$^{\bullet}OOH$	$^{\bullet}OCH_3$	$^{\bullet}OOCH_3$	$^{\bullet}OOCH{=}CH_2$	$^{\bullet}OH$	$^{\bullet}OOH$	$^{\bullet}OCH_3$	$^{\bullet}OOCH_3$	$^{\bullet}OOCH{=}CH_2$
**C1**	-28.10	3.90	-13.19	5.49	2.60	-28.53	4.35	-13.03	5.61	2.06	-28.28	4.17	-12.81	6.03	2.69
**C2**	-16.86	15.14	-1.96	16.73	13.84	-17.65	15.23	-2.15	16.50	12.95	-16.86	15.58	-1.39	17.45	14.11
**C6**	-14.13	17.87	0.78	19.46	16.57	-16.53	16.34	-1.03	17.61	14.06	-14.28	18.16	1.19	20.03	16.69
**C7**	-23.56	8.44	-8.66	10.03	7.14	-27.87	5.01	-12.37	6.28	2.73	-24.66	7.79	-9.19	9.65	6.32
**C8**	-24.09	7.90	-9.19	9.49	6.60	-26.68	6.20	-11.18	7.47	3.92	-24.77	7.67	-9.30	9.54	6.20
**C8tBu**	-20.27	11.23	-5.87	12.82	9.93	-22.53	9.84	-7.53	11.11	7.56	-20.50	11.44	-5.53	13.31	9.97
**C10**	-34.35	-2.35	-19.45	-0.76	-3.65	-39.99	-7.11	-24.49	-5.84	-9.39	-36.06	-3.62	-20.59	-1.75	-5.09
**C12**	-17.45	14.55	-2.55	16.14	13.25	-18.40	14.48	-2.90	15.75	12.19	-17.23	15.22	-1.76	17.08	13.75
**C14**	-26.31	5.68	-11.41	7.28	4.39	-29.54	3.34	-14.04	4.61	1.05	-26.78	5.66	-11.32	7.53	4.19
**C14Me**	-17.31	14.69	-2.40	16.28	13.39	-19.97	12.91	-4.47	14.18	10.63	-17.58	14.86	-2.11	16.73	13.39
**C1OH**	-8.72	23.27	6.18	24.86	21.98	-9.68	23.20	5.82	24.47	20.92	-8.97	23.47	6.50	25.34	22.00
**C3OH**	-10.24	21.75	4.66	23.34	20.46	-11.94	20.94	3.56	22.21	18.66	-11.10	21.34	4.37	23.21	19.87
**C7OH**	-10.98	21.01	3.92	22.61	19.72	-13.90	18.98	1.60	20.25	16.70	-12.05	20.40	3.42	22.26	18.93
**C10OH**	-10.10	21.89	4.80	23.48	20.60	-13.18	19.70	2.32	20.97	17.42	-11.11	21.33	4.35	23.20	19.86

Figure S3 Gibbs free reaction energies (ΔG^{o}_{HAT}) computed in (a) gas-phase, (b) water and (c) benzene for the scavenging of •OH, •OOH, •OCH₃, •OOCH₃ and •OOCH=CH₂ via HAT from all the available sites of **1J**. Level of theory: (SMD)-M06-2X/6-311+G(d,p)//M06-2X/6-31G(d).

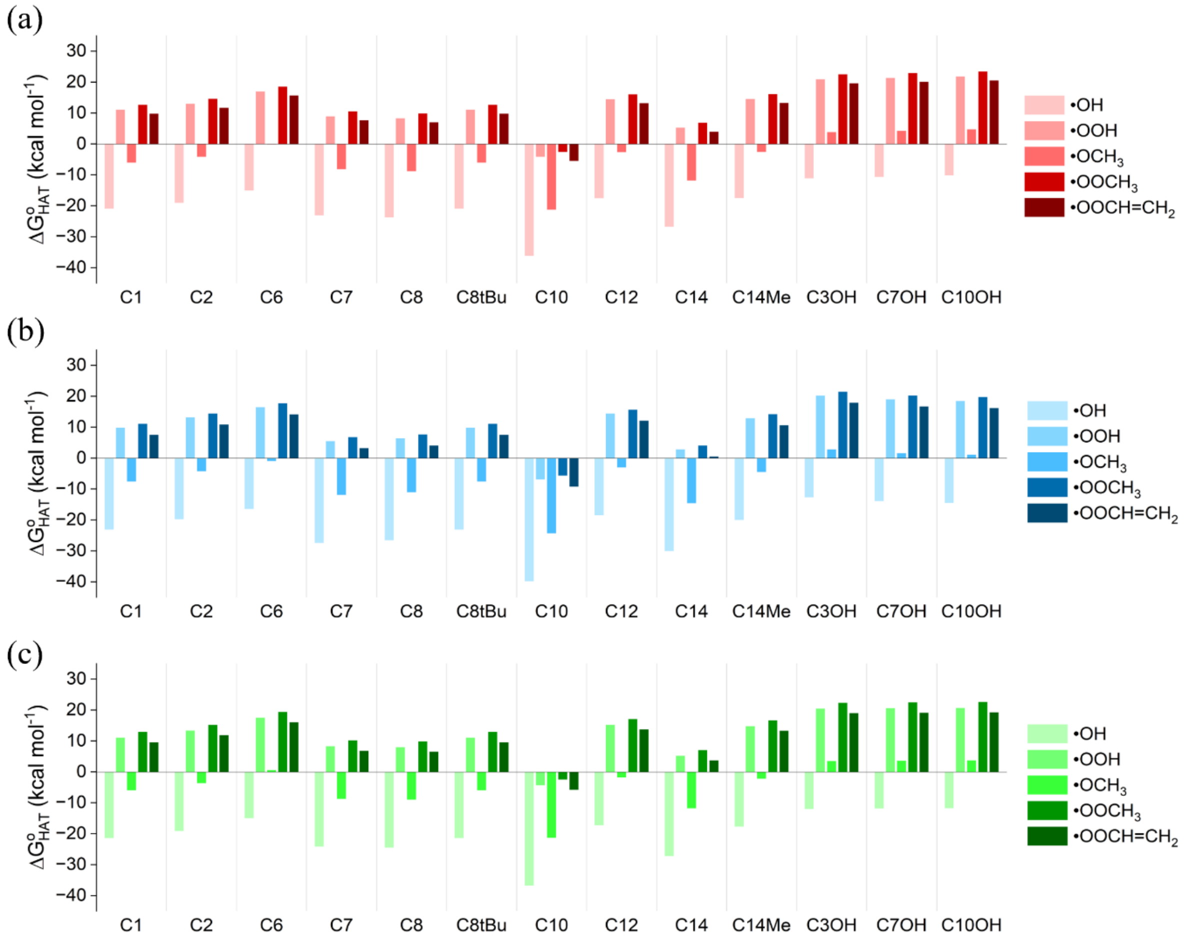

Table S4 Gibbs free reaction energies (ΔG^{o}_{HAT}) computed in gas-phase, water and benzene for the scavenging of •OH, •OOH, •OCH₃, •OOCH₃ and •OOCH=CH₂ via HAT from all the available sites of **1J**. Level of theory: (SMD)-M06-2X/6-311+G(d,p)//M06-2X/6-31G(d).

	ΔG^{o}_{HAT}					$\Delta G^{o}_{HAT,water}$					$\Delta G^{o}_{HAT,benzene}$				
	•OH	•OOH	•OCH₃	•OOCH₃	•OOCH=CH₂	•OH	•OOH	•OCH₃	•OOCH₃	•OOCH=CH₂	•OH	•OOH	•OCH₃	•OOCH₃	•OOCH=CH₂
**C1**	-20.92	11.07	-6.02	12.66	9.78	-23.08	9.80	-7.58	11.07	7.52	-21.41	11.03	-5.94	12.90	9.56
**C2**	-19.02	12.98	-4.12	14.57	11.68	-19.75	13.13	-4.25	14.40	10.85	-19.11	13.33	-3.65	15.20	11.86
**C6**	-15.03	16.96	-0.13	18.55	15.66	-16.45	16.43	-0.95	17.70	14.14	-14.94	17.51	0.53	19.37	16.03
**C7**	-23.09	8.91	-8.19	10.50	7.61	-27.39	5.48	-11.89	6.75	3.20	-24.15	8.29	-8.69	10.16	6.82
**C8**	-23.70	8.29	-8.80	9.88	6.99	-26.54	6.34	-11.03	7.61	4.06	-24.46	7.99	-8.99	9.85	6.52
**C8tBu**	-20.92	11.07	-6.02	12.66	9.78	-23.08	9.80	-7.58	11.07	7.52	-21.41	11.03	-5.94	12.90	9.56
**C10**	-36.14	-4.15	-21.24	-2.56	-5.45	-39.81	-6.94	-24.31	-5.67	-9.22	-36.77	-4.33	-21.31	-2.46	-5.80
**C12**	-17.54	14.46	-2.63	16.05	13.16	-18.51	14.37	-3.01	15.64	12.08	-17.26	15.18	-1.80	17.05	13.71
**C14**	-26.75	5.25	-11.84	6.84	3.95	-30.08	2.80	-14.58	4.07	0.51	-27.25	5.19	-11.78	7.06	3.72
**C14Me**	-17.45	14.55	-2.55	16.14	13.25	-19.98	12.89	-4.48	14.16	10.61	-17.68	14.76	-2.22	16.63	13.29
**C3OH**	-11.10	20.89	3.80	22.48	19.60	-12.70	20.18	2.80	21.44	17.89	-11.99	20.45	3.48	22.32	18.98
**C7OH**	-10.67	21.32	4.23	22.92	20.03	-13.91	18.97	1.59	20.23	16.68	-11.85	20.59	3.62	22.46	19.12
**C10OH**	-10.19	21.81	4.72	23.40	20.51	-14.44	18.44	1.06	19.71	16.15	-11.75	20.69	3.72	22.56	19.22

Figure S4 Gibbs free reaction energies (ΔG^{o}_{HAT}) computed in (a) gas-phase, (b) water and (c) benzene for the scavenging of •OH, •OOH, •OCH3, •OOCH3 and •OOCH=CH2 via HAT from all the available sites of **1M**. Level of theory: (SMD)-M06-2X/6-311+G(d,p)//M06-2X/6-31G(d).

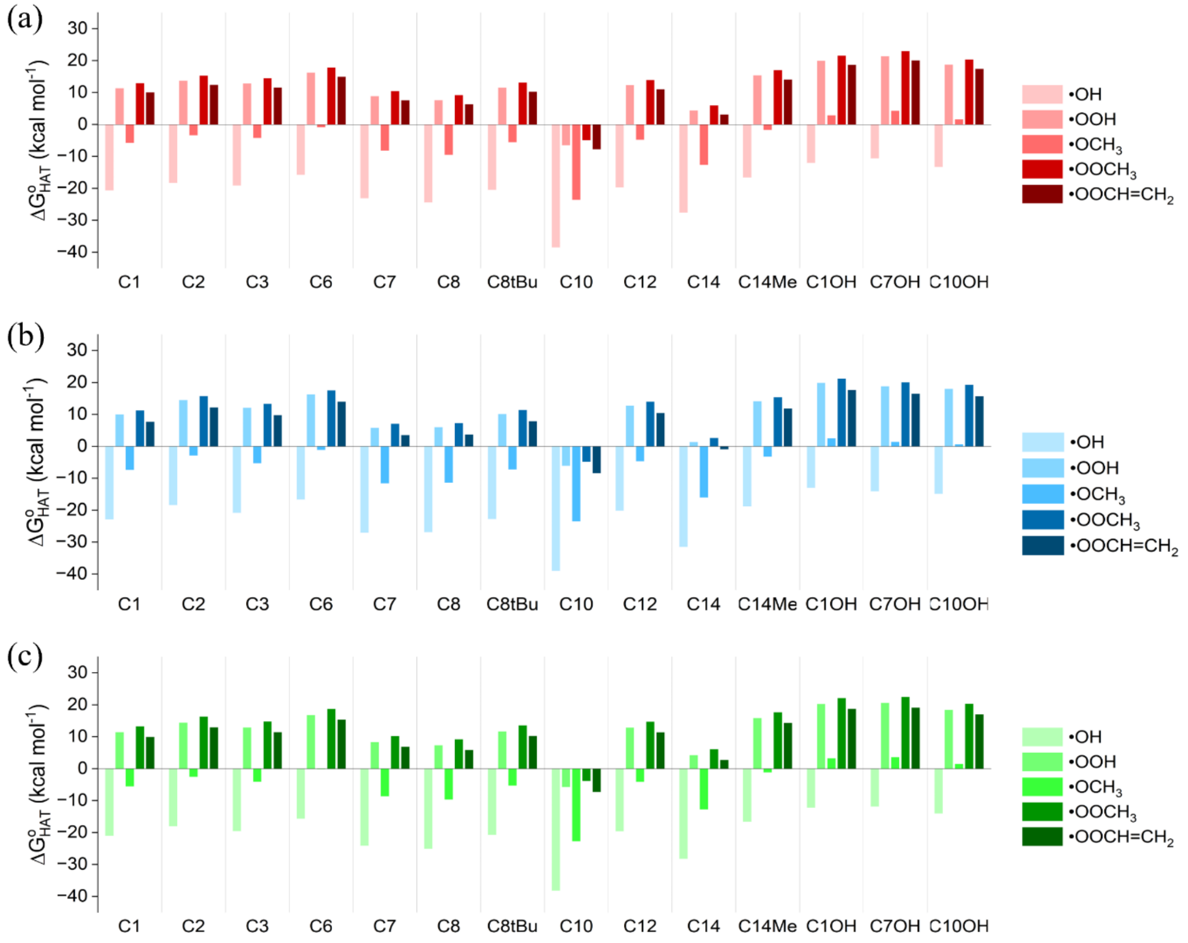

Table S5 Gibbs free reaction energies (ΔG^{o}_{HAT}) computed in gas-phase, water and benzene for the scavenging of •OH, •OOH, •OCH3, •OOCH3 and •OOCH=CH2 via HAT from all the available sites of **1M**. Level of theory: (SMD)-M06-2X/6-311+G(d,p)//M06-2X/6-31G(d).

	ΔG^{o}_{HAT}					$\Delta G^{o}_{HAT,water}$					$\Delta G^{o}_{HAT,benzene}$				
	•OH	•OOH	•OCH3	•OOCH3	•OOCH=CH2	•OH	•OOH	•OCH3	•OOCH3	•OOCH=CH2	•OH	•OOH	•OCH3	•OOCH3	•OOCH=CH2
**C1**	-20.67	11.33	-5.76	12.92	10.03	-22.91	9.97	-7.40	11.24	7.69	-21.04	11.40	-5.57	13.27	9.93
**C2**	-18.30	13.69	-3.40	15.29	12.40	-18.40	14.48	-2.90	15.75	12.20	-18.03	14.42	-2.56	16.28	12.95
**C3**	-19.12	12.87	-4.22	14.46	11.58	-20.81	12.06	-5.31	13.33	9.78	-19.55	12.90	-4.08	14.76	11.42
**C6**	-15.78	16.22	-0.87	17.81	14.92	-16.63	16.25	-1.13	17.52	13.97	-15.63	16.81	-0.16	18.68	15.34
**C7**	-23.13	8.87	-8.23	10.46	7.57	-27.08	5.80	-11.58	7.07	3.52	-24.12	8.32	-8.65	10.19	6.85
**C8**	-24.40	7.60	-9.49	9.19	6.30	-26.88	6.00	-11.38	7.27	3.71	-25.13	7.32	-9.66	9.18	5.85
**C8tBu**	-20.46	11.54	-5.56	13.13	10.24	-22.74	10.14	-7.24	11.41	7.85	-20.76	11.69	-5.29	13.55	10.21
**C10**	-38.50	-6.50	-23.60	-4.91	-7.80	-38.99	-6.12	-23.49	-4.85	-8.40	-38.22	-5.77	-22.75	-3.91	-7.24
**C12**	-19.69	12.31	-4.78	13.90	11.01	-20.17	12.71	-4.67	13.98	10.42	-19.59	12.85	-4.12	14.72	11.38
**C14**	-27.60	4.39	-12.70	5.99	3.10	-31.51	1.36	-16.01	2.63	-0.92	-28.24	4.21	-12.77	6.07	2.74
**C14Me**	-16.61	15.39	-1.70	16.98	14.09	-18.77	14.11	-3.27	15.38	11.82	-16.64	15.81	-1.17	17.67	14.34
**C1OH**	-12.03	19.96	2.87	21.56	18.67	-12.98	19.89	2.52	21.16	17.61	-12.23	20.21	3.24	22.08	18.74
**C7OH**	-10.63	21.37	4.27	22.96	20.07	-14.11	18.77	1.39	20.04	16.48	-11.86	20.58	3.61	22.45	19.11
**C10OH**	-13.28	18.72	1.63	20.31	17.42	-14.88	17.99	0.62	19.26	15.71	-14.01	18.44	1.46	20.30	16.96

Table S6 Gibbs free reaction energies (ΔG^{o}_{HAT}) computed in gas-phase, water and benzene for the scavenging of •OH, •OOH, •OCH₃, •OOCH₃ and •OOCH=CH₂ via HAT from all the available sites of **1B⁻**. Level of theory: (SMD)-M06-2X/6-311+G(d,p)//M06-2X/6-31G(d).

	ΔG^{o}_{HAT}					$\Delta G^{o}_{HAT,water}$					$\Delta G^{o}_{HAT,benzene}$				
	•OH	•OOH	$^{\bullet}OCH_3$	$^{\bullet}OOCH_3$	$^{\bullet}OOCH{=}CH_2$	•OH	•OOH	$^{\bullet}OCH_3$	$^{\bullet}OOCH_3$	$^{\bullet}OOCH{=}CH_2$	•OH	•OOH	$^{\bullet}OCH_3$	$^{\bullet}OOCH_3$	$^{\bullet}OOCH{=}CH_2$
**C1**	-20.16	11.83	-5.26	13.43	10.54	-22.25	10.63	-6.75	11.90	8.35	-20.51	11.93	-5.05	13.80	10.46
**C2**	-27.61	4.38	-12.71	5.98	3.09	-25.69	7.19	-10.19	8.46	4.91	-26.71	5.74	-11.24	7.60	4.27
**C6**	-18.50	13.49	-3.60	15.09	12.20	-19.36	13.52	-3.86	14.79	11.23	-18.56	13.88	-3.09	15.75	12.41
**C7**	-22.30	9.69	-7.40	11.28	8.40	-24.53	8.35	-9.03	9.62	6.06	-22.77	9.67	-7.30	11.54	8.20
**C8**	-21.88	10.12	-6.98	11.71	8.82	-24.06	8.82	-8.56	10.09	6.54	-22.46	9.98	-6.99	11.85	8.51
**C8tBu**	-19.97	12.03	-5.06	13.62	10.73	-22.71	10.17	-7.21	11.44	7.89	-20.41	12.03	-4.94	13.90	10.56
**C10**	-38.92	-6.93	-24.02	-5.33	-8.22	-38.44	-5.56	-22.94	-4.29	-7.85	-38.47	-6.03	-23.00	-4.16	-7.50
**C12**	-20.61	11.39	-5.71	12.98	10.09	-19.23	13.65	-3.73	14.92	11.37	-19.87	12.57	-4.41	14.44	11.10
**C14**	-19.55	12.45	-4.64	14.04	11.15	-22.26	10.61	-6.76	11.88	8.33	-20.12	12.32	-4.65	14.19	10.85
**C14Me**	-18.57	13.42	-3.67	15.01	12.12	-20.98	11.90	-5.48	13.16	9.61	-18.76	13.69	-3.29	15.55	12.21
**C1OH**	-7.14	24.85	7.76	26.45	23.56	-13.91	18.96	1.59	20.23	16.68	-9.27	23.17	6.20	25.04	21.70
**C3OH**	-10.84	21.16	4.07	22.75	19.86	-15.10	17.78	0.40	19.05	15.49	-12.24	20.20	3.23	22.07	18.73
**C10OH**	-23.41	8.58	-8.51	10.17	7.28	-21.65	11.23	-6.15	12.50	8.94	-23.13	9.31	-7.67	11.18	7.84
**C2OH**	1.13	33.13	16.03	34.72	31.83	-4.22	28.66	11.28	29.93	26.37	0.78	33.22	16.25	35.09	31.75

Table S7 Gibbs free reaction energies (ΔG^{o}_{HAT} kcal mol⁻¹) computed in water of **1B** and **1B⁻** for the scavenging of ·OH via HAT from C1, C2, C14 and C3OH sites. Level of theory: M06-2X/6-311+G(d,p)//M06-2X/6-31G(d).

Site	ΔG^{o}_{HAT} (1B)	ΔG^{o}_{HAT} (1B⁻)	$\Delta\Delta G^{o}_{HAT}$
C1	-28.64	-22.25	6.39
C2	-17.70	-25.69	-7.99
C10	-39.35	-38.44	0.91
C14	-29.59	-22.26	7.33
C3OH	-12.49	-21.65	-9.16

Table S8 Gibbs free reaction energies (ΔG^{o}_{HAT} kcal mol^{-1}) computed in gas-phase, water and benzene, for the scavenging of •OH, •OOH, •OCH$_3$, •OOCH$_3$ and •OOCH=CH$_2$ via HAT from neutral Trolox[a] (**3**, Scheme 1 in the text). For comparison the values of HAT from C10 of **1B** and **1B**$^-$ are also included. Level of theory: (SMD)-M06-2X/6-311+G(d,p)//M06-2X/6-31G(d).

	ΔG^{o}_{HAT}					$\Delta G^{o}_{HAT,water}$					$\Delta G^{o}_{HAT,benzene}$				
	•OH	•OOH	•OCH$_3$	•OOCH$_3$	•OOCH=CH$_2$	•OH	•OOH	•OCH$_3$	•OOCH$_3$	•OOCH=CH$_2$	•OH	•OOH	•OCH$_3$	•OOCH$_3$	•OOCH=CH$_2$
**C1**	-28.3	3.3	-13.8	4.9	2	-31.0	1.5	-15.9	2.8	-0.8	-28.6	3.5	-13.5	5.3	2.0
**C2**	-27.4	4.2	-12.9	5.8	2.9	-29.5	2.9	-14.4	4.2	0.6	-27.3	4.7	-12.3	6.6	3.3
**C3**	-27.7	3.9	-13.2	5.4	2.6	-30.4	2.1	-15.3	3.3	-0.2	-27.9	4.1	-12.9	6.0	2.6
**C4**	-16.6	15.0	-2.1	16.6	13.7	-19.0	13.5	-3.9	14.7	11.2	-16.9	15.2	-1.8	17.0	13.7
**C5**	-19.4	12.2	-4.9	13.8	10.9	-21.7	10.7	-6.6	12.0	8.5	-19.7	12.4	-4.6	14.2	10.9
**C6**	-31.4	0.2	-16.9	1.8	-1.2	-33.5	-1.0	-18.4	0.2	-3.3	-31.7	0.3	-16.7	2.2	-1.2
**O1**	-38.7	-7.1	-24.2	-5.5	-8.4	-41.7	-9.3	-26.7	-8.0	-11.6	-39.9	-7.8	-24.8	-6.0	-9.3
**O2**	-6.6	25.0	8.0	26.6	23.7	-5.6	26.9	9.5	28.2	24.6	-7.2	24.9	7.9	26.7	23.4
**1B - C10**	-34.4	-2.4	-19.5	-0.8	-3.7	-39.4	-6.5	-23.9	-5.2	-8.8	-35.9	-3.5	-20.5	-1.6	-5.0
**1B$^-$ - C10**	-38.9	-6.9	-24.0	-5.3	-8.2	-38.4	-5.6	-22.9	-4.3	-7.9	-38.5	-6.0	-23.0	-4.2	-7.5

[a] Bortoli, M.; Dalla Tiezza, M.; Muraro, C.; Pavan, C.; Ribaudo, G.; Rodighiero, A.; Tubaro, C.; Zagotto, G.; Orian, L. Psychiatric Disorders and Oxidative Injury: Antioxidant Effects of Zolpidem Therapy Disclosed In Silico. *Computational and Structural Biotechnology Journal* **2019**, *17*, 311–318, doi:10.1016/j.csbj.2019.02.004.

Table S9 Gibbs free reaction energies (ΔG^{o}_{HAT} kcal mol^{-1}) computed in gas-phase, water and benzene, for the scavenging of •OH, •OOH, •OCH$_3$, •OOCH$_3$ and •OOCH=CH$_2$ via HAT from C4 site of melatonin[a] (**4**, Scheme 1 in the text). Two lowest energy conformers of melatonin have been considered[b]. For comparison the values of HAT from C10 of **1B** and **1B**$^-$ are also included. Level of theory: (SMD)-M06-2X/6-311+G(d,p)//M06-2X/6-31G(d).

	ΔG^{o}_{HAT}					$\Delta G^{o}_{HAT,water}$					$\Delta G^{o}_{HAT,benzene}$				
	•OH	•OOH	•OCH$_3$	•OOCH$_3$	•OOCH=CH$_2$	•OH	•OOH	•OCH$_3$	•OOCH$_3$	•OOCH=CH$_2$	•OH	•OOH	•OCH$_3$	•OOCH$_3$	•OOCH=CH$_2$
**C1**	-28.9	2.8	-14.3	4.3	1.5	-33.8	-1.3	-18.7	-0.1	-3.6	-31.3	0.8	-16.2	2.7	-0.7
**C2**	-28.6	3.0	-14.1	4.5	1.7	-31.0	1.4	-16.0	2.7	-0.9	-29.1	3.0	-14.0	4.9	1.5
**1B - C10**	-34.4	-2.4	-19.5	-0.8	-3.7	-39.4	-6.5	-23.9	-5.2	-8.8	-35.9	-3.5	-20.5	-1.6	-5.0
**1B$^-$ - C10**	-38.9	-6.9	-24.0	-5.3	-8.2	-38.4	-5.6	-22.9	-4.3	-7.9	-38.5	-6.0	-23.0	-4.2	-7.5

[a] Bortoli, M.; Dalla Tiezza, M.; Muraro, C.; Pavan, C.; Ribaudo, G.; Rodighiero, A.; Tubaro, C.; Zagotto, G.; Orian, L. Psychiatric Disorders and Oxidative Injury: Antioxidant Effects of Zolpidem Therapy Disclosed In Silico. *Computational and Structural Biotechnology Journal* **2019**, *17*, 311–318, doi:10.1016/j.csbj.2019.02.004 [b] Fogueri, U.R.; Kozuch, S.; Karton, A.; Martin, J.M.L. The Melatonin Conformer Space: Benchmark and Assessment of Wave Function and DFT Methods for a Paradigmatic Biological and Pharmacological Molecule. *J Phys Chem A* **2013**, *117*, 2269–2277, doi:10.1021/jp312644t.

Figure S5 Gibbs free reaction energies (ΔG^{o}_{HAT}) computed in (a) gas-phase, (b) water and (c) benzene for the scavenging of $^{\bullet}$OH, $^{\bullet}$OOH, $^{\bullet}OCH_3$, $^{\bullet}OOCH_3$ and $^{\bullet}OOCH{=}CH_2$ via HAT from all the available sites of **2**. Level of theory: (SMD)-M06-2X/6-311+G(d,p)//M06-2X/6-31G(d).

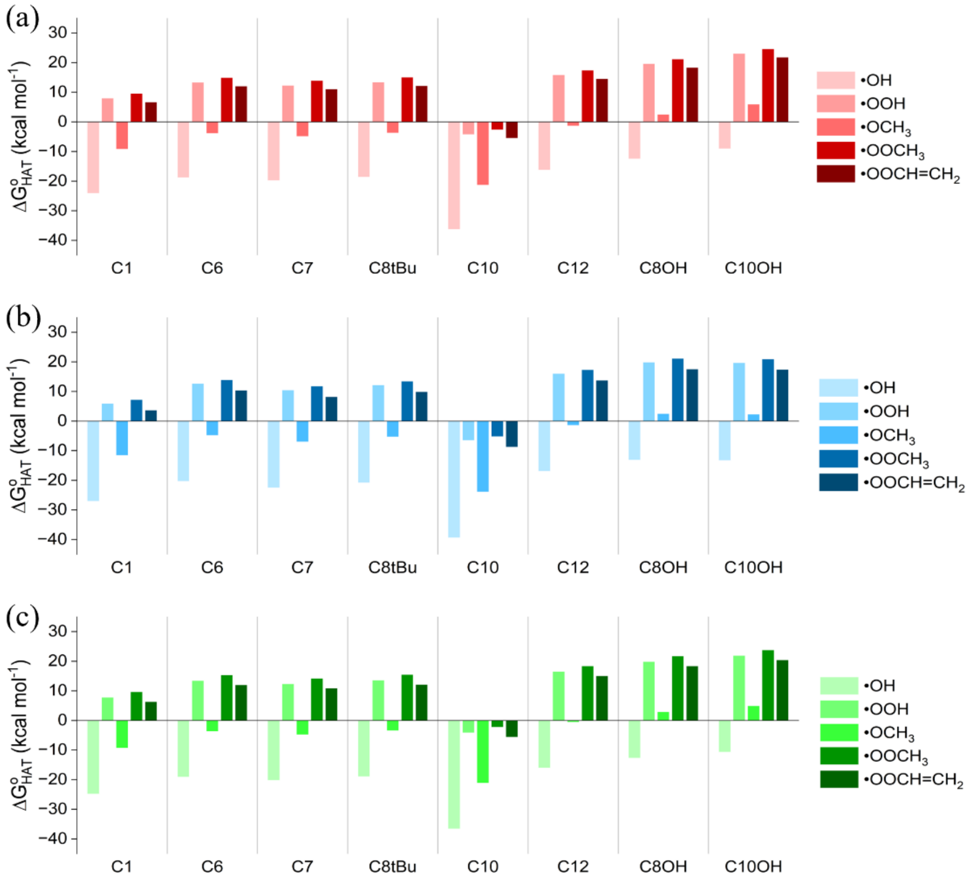

Table S10 Gibbs free reaction energies (ΔG^{o}_{HAT}) computed in gas-phase, water and benzene for the scavenging of $^{\bullet}$OH, $^{\bullet}$OOH, $^{\bullet}OCH_3$, $^{\bullet}OOCH_3$ and $^{\bullet}OOCH{=}CH_2$ via HAT from all the available sites of **2**. Level of theory: (SMD)-M06-2X/6-311+G(d,p)//M06-2X/6-31G(d).

	ΔG^{o}_{HAT}					$\Delta G^{o}_{HAT,water}$					$\Delta G^{o}_{HAT,benzene}$				
	$^{\bullet}$OH	$^{\bullet}$OOH	$^{\bullet}OCH_3$	$^{\bullet}OOCH_3$	$^{\bullet}OOCH{=}CH_2$	$^{\bullet}$OH	$^{\bullet}$OOH	$^{\bullet}OCH_3$	$^{\bullet}OOCH_3$	$^{\bullet}OOCH{=}CH_2$	$^{\bullet}$OH	$^{\bullet}$OOH	$^{\bullet}OCH_3$	$^{\bullet}OOCH_3$	$^{\bullet}OOCH{=}CH_2$
C1	-24.07	7.92	-9.17	9.51	6.62	-26.98	5.90	-11.48	7.17	3.61	-24.71	7.74	-9.24	9.60	6.26
C6	-18.70	13.29	-3.80	14.88	11.99	-20.25	12.62	-4.75	13.89	10.34	-19.04	13.40	-3.57	15.27	11.93
C7	-19.72	12.28	-4.81	13.87	10.98	-22.44	10.44	-6.94	11.71	8.15	-20.17	12.28	-4.70	14.14	10.80
C8tBu	-18.58	13.41	-3.68	15.01	12.12	-20.76	12.11	-5.26	13.38	9.83	-18.87	13.58	-3.40	15.44	12.10
C10	-36.17	-4.18	-21.27	-2.58	-5.47	-39.33	-6.45	-23.83	-5.18	-8.73	-36.52	-4.08	-21.05	-2.21	-5.55
C12	-16.20	15.80	-1.29	17.39	14.50	-16.88	16.00	-1.38	17.27	13.71	-15.95	16.50	-0.48	18.36	15.03
C8OH	-12.42	19.57	2.48	21.16	18.27	-13.06	19.81	2.44	21.08	17.53	-12.61	19.83	2.86	21.70	18.36
C10OH	-8.98	23.02	5.92	24.61	21.72	-13.25	19.62	2.25	20.89	17.34	-10.58	21.86	4.89	23.73	20.39

Table S11 Coordinates of the structures of **1A-M**, **1B$^-$**, **2** and their radicals at each available site. Level of theory: M06-2X/6-31G(d).

1A

C	0.092454	-0.044483	0.011545
C	0.359148	1.432661	-0.300989
C	-0.956837	1.980947	-0.892187
C	-1.628099	0.751205	-1.548557
C	-0.849352	-0.499056	-1.116728
C	-0.120983	-0.945706	-2.410626
C	-0.576530	-2.373641	-2.648291
C	-2.048890	-2.353941	-2.207816
C	-1.975215	-1.560299	-0.871827
C	-1.838113	-2.325932	0.437781
C	-2.344396	-1.318178	1.456658
C	-3.186292	-0.647157	-0.578528
C	-1.487383	0.809459	-3.071237
C	-1.655110	2.523401	0.358961
C	-0.473961	2.899959	1.247155
C	-2.621524	3.675810	0.141868
C	-2.771157	-3.724038	-2.277555
C	-4.188764	-3.633876	-1.690918
C	-1.970802	-4.855594	-1.610089
C	-2.934583	-4.088809	-3.763977
O	-0.626897	2.994609	-1.808005
O	-0.549266	-2.783252	0.724149
O	-2.045388	-1.286979	2.616588
O	-3.183955	-0.451182	0.855804
O	-3.001337	0.565498	-1.223877
O	-0.596688	-0.109926	-3.482673
O	-2.070217	1.554058	-3.806276
O	-0.461638	3.663211	2.165393
O	0.635123	2.202583	0.861149
H	-0.379592	-0.095128	0.997253
H	1.181780	1.579064	-1.007122
H	0.963799	-0.822613	-2.364108
H	-0.421692	-2.678087	-3.685799
H	0.000252	-3.034160	-1.989926
H	-2.589616	-1.697494	-2.910009
H	-2.558805	-3.155925	0.456237
H	-4.169983	-1.029532	-0.852543
H	-2.174524	1.702048	0.870680
H	-3.482967	3.344354	-0.444817
H	-2.124985	4.499509	-0.376190
H	-2.971570	4.044284	1.108288
H	-4.210097	-3.437006	-0.614375
H	-4.716649	-4.579382	-1.852466
H	-4.766087	-2.847118	-2.191176
H	-1.639545	-4.629241	-0.593394
H	-1.074558	-5.089457	-2.193875
H	-2.581158	-5.764208	-1.574212
H	-3.524053	-3.333957	-4.295865
H	-3.452433	-5.049202	-3.858044
H	-1.968374	-4.184959	-4.267847
H	-0.449925	-2.751738	1.692775
H	-1.409436	3.203660	-2.345334
H	1.000267	-0.648413	0.065788

C1

C	0.100508	-0.062292	-0.082371
C	0.308029	1.414060	-0.160158
C	-0.952538	1.975256	-0.863354
C	-1.601680	0.746322	-1.567174
C	-0.815364	-0.513586	-1.172321
C	-0.131076	-0.963400	-2.501825
C	-0.610671	-2.384147	-2.733786
C	-2.061567	-2.365486	-2.227194
C	-1.930790	-1.582468	-0.893098
C	-1.730597	-2.331123	0.416718
C	-2.114188	-1.271386	1.436609
C	-3.116366	-0.660072	-0.544592
C	-1.475316	0.842768	-3.087558
C	-1.755686	2.545361	0.310731
C	-0.692044	2.806221	1.367519
C	-2.601086	3.771006	0.004637
C	-2.790650	-3.732779	-2.270274
C	-4.185626	-3.631821	-1.634514
C	-1.978108	-4.864837	-1.620610
C	-3.003171	-4.101823	-3.749051
O	-0.524694	2.957045	-1.773545
O	-0.449183	-2.846995	0.626800
O	-1.716002	-1.203162	2.562670
O	-3.008673	-0.424410	0.878161
O	-2.971912	0.528215	-1.244933
O	-0.634784	-0.104710	-3.540894
O	-2.035016	1.631864	-3.793979
O	-0.767633	3.525387	2.318085
O	0.416470	2.055508	1.107078
H	1.202737	1.686442	-0.736863
H	0.955164	-0.851931	-2.487971
H	-0.505568	-2.676235	-3.781162
H	-0.006997	-3.055345	-2.111694
H	-2.630855	-1.702409	-2.899993
H	-2.488422	-3.123646	0.506118
H	-4.117949	-1.043155	-0.741946
H	-2.390961	1.756356	0.728901
H	-3.400167	3.517320	-0.698603
H	-1.986779	4.568237	-0.420223
H	-3.049516	4.141823	0.928507
H	-4.162380	-3.439691	-0.557112
H	-4.727823	-4.571938	-1.779223
H	-4.773644	-2.837390	-2.109664
H	-1.637541	-4.641674	-0.606447
H	-1.088242	-5.093974	-2.215866
H	-2.586112	-5.774973	-1.582100
H	-3.597707	-3.341231	-4.267067
H	-3.536625	-5.055342	-3.823238
H	-2.053579	-4.213759	-4.280570
H	-0.285791	-2.794417	1.586145
H	-1.274181	3.209862	-2.338931
H	0.755339	-0.725664	0.464786

C2

C	0.179493	-0.136418	0.028012
C	0.081899	1.354989	0.095199
C	-0.991629	1.958886	-0.765310
C	-1.634257	0.727117	-1.456914
C	-0.814597	-0.529980	-1.097500
C	-0.157477	-0.934111	-2.442145
C	-0.611763	-2.359316	-2.697235
C	-2.061808	-2.361033	-2.187530
C	-1.924898	-1.611868	-0.831554
C	-1.736295	-2.419774	0.444440
C	-2.155007	-1.426729	1.519193
C	-3.115796	-0.713651	-0.445291
C	-1.543066	0.848605	-2.978301
C	-1.845856	2.699830	0.273628
C	-0.841411	2.946925	1.392595
C	-2.545605	3.963330	-0.195824
C	-2.787888	-3.728326	-2.273496
C	-4.183452	-3.655736	-1.634153
C	-1.969860	-4.882476	-1.670923
C	-3.004490	-4.044520	-3.764378
O	-0.388264	2.830872	-1.700625
O	-0.452086	-2.933304	0.642043
O	-1.804708	-1.447430	2.664707
O	-2.988004	-0.510141	0.984030
O	-2.994015	0.493360	-1.106833
O	-0.703814	-0.078559	-3.464360
O	-2.126769	1.646873	-3.655992
O	-0.863324	3.763637	2.257856
O	0.169603	2.001386	1.298813
H	-0.065019	-0.557867	1.005822
H	0.925273	-0.793837	-2.454314
H	-0.505426	-2.633471	-3.749163
H	-0.000188	-3.033442	-2.085796
H	-2.634556	-1.681773	-2.840408

H	-2.485548	-3.224440	0.476765
H	-4.115787	-1.105011	-0.635871
H	-2.574834	1.991044	0.688095
H	-3.279780	3.723118	-0.971152
H	-1.822428	4.678302	-0.595091
H	-3.062425	4.434696	0.642732
H	-4.164183	-3.508025	-0.549749
H	-4.723571	-4.590226	-1.817956
H	-4.772489	-2.843969	-2.077725
H	-1.624213	-4.699171	-0.650615
H	-1.082017	-5.084825	-2.278809
H	-2.575093	-5.795302	-1.665866
H	-3.604474	-3.268460	-4.252212
H	-3.533999	-4.997213	-3.870768
H	-2.056711	-4.132748	-4.303312
H	-0.312417	-2.978277	1.605004
H	-1.080744	3.157231	-2.301438
H	1.187394	-0.501186	-0.202390

C6

C	0.113670	-0.056681	-0.059143
C	0.372098	1.428181	-0.336467
C	-0.955735	1.995525	-0.882281
C	-1.627533	0.791050	-1.581106
C	-0.868790	-0.479606	-1.168683
C	-0.229642	-0.939901	-2.467606
C	-0.582200	-2.374632	-2.693518
C	-2.061644	-2.375496	-2.223880
C	-2.001007	-1.538769	-0.907591
C	-1.882028	-2.271577	0.422985
C	-2.393854	-1.236275	1.408545
C	-3.213228	-0.612293	-0.651734
C	-1.448354	0.884880	-3.100914
C	-1.633730	2.484765	0.401259
C	-0.435097	2.839206	1.276405
C	-2.612502	3.637223	0.248582
C	-2.762839	-3.757285	-2.254509
C	-4.190498	-3.660000	-1.692566
C	-1.958800	-4.854784	-1.537644
C	-2.900248	-4.176139	-3.729319
O	-0.639736	3.050406	-1.755334
O	-0.602989	-2.737302	0.732883
O	-2.097815	-1.161843	2.567001
O	-3.242847	-0.398955	0.777728
O	-3.010464	0.597176	-1.303924
O	-0.606334	-0.102719	-3.514111
O	-1.958195	1.669044	-3.842713
O	-0.409498	3.571237	2.219551
O	0.669582	2.165414	0.842152
H	-0.327166	-0.139559	0.938775
H	1.180385	1.592324	-1.054473
H	-0.446810	-2.680561	-3.732092
H	0.018612	-3.014843	-2.037846
H	-2.612878	-1.745828	-2.942705
H	-2.612359	-3.092832	0.453322
H	-4.192743	-0.990845	-0.946285
H	-2.137076	1.643573	0.895513
H	-3.485381	3.322020	-0.330092
H	-2.133341	4.484292	-0.247813
H	-2.942676	3.966305	1.235957
H	-4.231640	-3.418966	-0.625776
H	-4.705466	-4.617366	-1.823213
H	-4.768272	-2.900833	-2.233501
H	-1.660314	-4.599082	-0.518219
H	-1.042442	-5.083802	-2.091346
H	-2.552001	-5.774560	-1.497475
H	-3.478348	-3.440710	-4.299730
H	-3.418700	-5.138326	-3.797126
H	-1.925237	-4.293480	-4.211212
H	-0.505342	-2.669985	1.699829
H	-1.431771	3.295083	-2.261769
H	1.020815	-0.662604	-0.061847

C7

C	0.076894	-0.055556	0.045735
C	0.367112	1.416118	-0.269316
C	-0.936059	1.978197	-0.873443
C	-1.608439	0.755934	-1.545693
C	-0.854813	-0.504804	-1.093906
C	-0.106332	-0.972174	-2.370399
C	-0.682500	-2.302802	-2.680962
C	-2.091777	-2.353581	-2.182972
C	-2.000850	-1.543322	-0.851967
C	-1.893223	-2.291469	0.470175
C	-2.409459	-1.263333	1.464496
C	-3.204917	-0.611541	-0.594205
C	-1.411770	0.826886	-3.061302
C	-1.643735	2.523371	0.371318
C	-0.467590	2.890287	1.270791
C	-2.598659	3.684050	0.146981
C	-2.757375	-3.754207	-2.229449
C	-4.181346	-3.707413	-1.655580
C	-1.906925	-4.828181	-1.534120
C	-2.891508	-4.149205	-3.710853
O	-0.585843	2.994609	-1.778565
O	-0.615516	-2.756138	0.785188
O	-2.133777	-1.217320	2.629418
O	-3.230647	-0.397316	0.835543
O	-2.991147	0.587984	-1.255386
O	-0.460225	-0.044150	-3.438203
O	-1.989168	1.559652	-3.812915
O	-0.458388	3.652808	2.189658
O	0.640027	2.185740	0.893909
H	-0.410700	-0.093601	1.024262
H	1.198442	1.550046	-0.967707
H	0.981826	-0.941622	-2.284780
H	-0.276905	-2.945737	-3.451252
H	-2.705891	-1.720222	-2.858843
H	-2.623530	-3.112565	0.489655
H	-4.186393	-0.989551	-0.885473
H	-2.175037	1.706538	0.877493
H	-3.456534	3.361100	-0.449573
H	-2.090256	4.505556	-0.363004
H	-2.955894	4.052528	1.110765
H	-4.221417	-3.471291	-0.587876
H	-4.664835	-4.681095	-1.785262
H	-4.788784	-2.965682	-2.188125
H	-1.699349	-4.619937	-0.482500
H	-0.938390	-4.932262	-2.034010
H	-2.418017	-5.795044	-1.593992
H	-3.487211	-3.415493	-4.265044
H	-3.389856	-5.120640	-3.793526
H	-1.917743	-4.236706	-4.201791
H	-0.532950	-2.709996	1.754726
H	-1.361595	3.220012	-2.318972
H	0.974067	-0.672724	0.117463

C8

C	0.119174	-0.010323	0.038453
C	0.342852	1.461963	-0.327569
C	-0.992331	1.953204	-0.926786
C	-1.639671	0.683250	-1.528446
C	-0.815588	-0.527019	-1.067051
C	-0.085321	-0.981394	-2.358787
C	-0.488534	-2.431828	-2.570098
C	-1.879518	-2.503154	-2.001285
C	-1.903693	-1.614094	-0.780120
C	-1.750564	-2.343085	0.560499
C	-2.278254	-1.320641	1.549512
C	-3.148835	-0.727043	-0.506441
C	-1.530737	0.690131	-3.053370
C	-1.693959	2.523974	0.309875
C	-0.516441	2.962814	1.173552
C	-2.692015	3.641933	0.058843
C	-2.769775	-3.692195	-2.287092
C	-4.181437	-3.549958	-1.701302
C	-2.109106	-4.972024	-1.727813
C	-2.919954	-3.850671	-3.812309
O	-0.697668	2.938290	-1.884788
O	-0.450504	-2.761729	0.843364
O	-1.968609	-1.239065	2.704525
O	-3.140269	-0.495075	0.924858
O	-2.999762	0.475960	-1.172420
O	-0.600042	-0.195212	-3.447023

O	-2.158586	1.382259	-3.802361
O	-0.516502	3.759727	2.062839
O	0.607126	2.279733	0.804121
H	-0.348043	-0.040528	1.027603
H	1.155690	1.605128	-1.045649
H	0.994614	-0.818488	-2.325389
H	-0.408865	-2.713781	-3.623038
H	0.194445	-3.077527	-1.992744
H	-2.451226	-3.192951	0.583020
H	-4.123707	-1.137913	-0.758785
H	-2.187772	1.708326	0.855332
H	-3.548573	3.266507	-0.508200
H	-2.221335	4.458687	-0.493299
H	-3.044641	4.036686	1.013877
H	-4.185501	-3.435108	-0.611904
H	-4.760919	-4.449796	-1.930172
H	-4.708337	-2.698290	-2.144544
H	-1.953839	-4.917958	-0.645227
H	-1.134356	-5.143971	-2.195372
H	-2.741045	-5.841696	-1.939127
H	-3.311273	-2.933031	-4.263213
H	-3.614166	-4.668138	-4.033662
H	-1.966587	-4.088991	-4.291957
H	-0.334270	-2.684398	1.807328
H	-1.492498	3.109422	-2.417567
H	1.043742	-0.586775	0.108134

C8tBu

C	0.094105	-0.039080	0.011974
C	0.378742	1.437685	-0.285890
C	-0.924715	2.004057	-0.888387
C	-1.602213	0.786501	-1.560505
C	-0.841848	-0.475660	-1.127544
C	-0.111397	-0.925300	-2.420430
C	-0.573855	-2.350501	-2.661896
C	-2.044668	-2.329216	-2.218601
C	-1.979124	-1.522638	-0.892105
C	-1.885077	-2.296097	0.416367
C	-2.395649	-1.286620	1.429022
C	-3.187643	-0.599861	-0.617708
C	-1.448344	0.851728	-3.081428
C	-1.631380	2.546091	0.358156
C	-0.456743	2.903489	1.262601
C	-2.582347	3.710609	0.137539
C	-2.752199	-3.714085	-2.228316
C	-4.088103	-3.626368	-1.546710
C	-1.887656	-4.824936	-1.607370
C	-3.015475	-4.095816	-3.704299
O	-0.572920	3.019714	-1.793943
O	-0.611741	-2.776183	0.735748
O	-2.115599	-1.262325	2.594138
O	-3.209321	-0.404580	0.815787
O	-2.981550	0.613528	-1.255650
O	-0.576155	-0.085150	-3.493096
O	-2.011484	1.611512	-3.816220
O	-0.446662	3.660523	2.186026
O	0.648993	2.196237	0.885038
H	-0.389861	-0.094215	0.991825
H	1.210867	1.581224	-0.981435
H	0.974244	-0.810609	-2.368982
H	-0.420425	-2.656805	-3.699515
H	0.001787	-3.011811	-2.003736
H	-2.596906	-1.695923	-2.932201
H	-2.630265	-3.107932	0.398075
H	-4.167283	-0.979020	-0.908423
H	-2.165971	1.727226	0.858116
H	-3.440446	3.392584	-0.461348
H	-2.070703	4.532179	-0.369108
H	-2.939650	4.076534	1.102271
H	-4.459069	-4.461882	-0.962516
H	-4.830536	-2.932933	-1.932044
H	-1.493724	-4.574785	-0.618656
H	-1.032061	-5.050764	-2.251305
H	-2.479592	-5.741910	-1.520911
H	-3.664156	-3.361478	-4.192496
H	-3.506882	-5.072240	-3.756198
H	-2.079745	-4.158675	-4.270107
H	-0.535315	-2.738588	1.706340
H	-1.347334	3.241505	-2.337882
H	0.995102	-0.652905	0.071218

C10

C	0.125848	-0.040088	-0.076099
C	0.397345	1.446824	-0.333464
C	-0.921886	2.017498	-0.899927
C	-1.610425	0.812076	-1.582396
C	-0.826354	-0.447383	-1.207716
C	-0.146256	-0.884249	-2.526573
C	-0.574050	-2.327628	-2.738311
C	-2.025661	-2.352819	-2.237881
C	-1.947352	-1.513923	-0.927112
C	-1.790382	-2.149350	0.397492
C	-2.558448	-1.434858	1.389688
C	-3.193708	-0.623488	-0.663353
C	-1.512928	0.899860	-3.108548
C	-1.612149	2.518369	0.373149
C	-0.428073	2.855810	1.272304
C	-2.574543	3.681589	0.201000
C	-2.723117	-3.736814	-2.228437
C	-4.105552	-3.636929	-1.570650
C	-1.907236	-4.825490	-1.519767
C	-2.923569	-4.166451	-3.691100
O	-0.595139	3.054866	-1.788500
O	-0.752156	-2.896349	0.776491
O	-2.485104	-1.580596	2.588257
O	-3.353479	-0.521544	0.759852
O	-2.973345	0.629762	-1.226519
O	-0.687633	-0.058183	-3.572761
O	-2.084699	1.684699	-3.808746
O	-0.409303	3.581376	2.220135
O	0.678710	2.170243	0.855750
H	-0.359529	-0.126772	0.903091
H	1.217960	1.616785	-1.036361
H	0.937327	-0.737701	-2.526427
H	-0.456187	-2.634911	-3.780097
H	0.054709	-2.967909	-2.107599
H	-2.611453	-1.728865	-2.933304
H	-4.130977	-1.010398	-1.064209
H	-2.136900	1.679921	0.852458
H	-3.439065	3.373780	-0.393751
H	-2.076233	4.521104	-0.289295
H	-2.920142	4.016434	1.181103
H	-4.038263	-3.353666	-0.513575
H	-4.610783	-4.607158	-1.616487
H	-4.743042	-2.910674	-2.088716
H	-1.741962	-4.608547	-0.462809
H	-0.931570	-4.971766	-1.994405
H	-2.446359	-5.776617	-1.585540
H	-3.500545	-3.421607	-4.250373
H	-3.469355	-5.114820	-3.731119
H	-1.968132	-4.314266	-4.204333
H	-0.797882	-2.959157	1.753104
H	-1.381474	3.282192	-2.312838
H	1.032704	-0.649634	-0.040198

C12

C	0.111700	-0.026784	0.021521
C	0.359833	1.450665	-0.301425
C	-0.958702	1.973785	-0.910661
C	-1.607909	0.726723	-1.556980
C	-0.802814	-0.507073	-1.114587
C	-0.078043	-0.957030	-2.408629
C	-0.539023	-2.386558	-2.642802
C	-2.007719	-2.353100	-2.194906
C	-1.918499	-1.578904	-0.849208
C	-1.788011	-2.357585	0.455291
C	-2.391084	-1.406007	1.478887
C	-3.090866	-0.668331	-0.542303
C	-1.480369	0.771506	-3.077833
C	-1.675566	2.519629	0.329451
C	-0.507501	2.922410	1.223809
C	-2.658154	3.654155	0.092290
C	-2.773835	-3.694715	-2.270420
C	-4.170403	-3.550844	-1.644054

C	-1.991076	-4.863085	-1.647302
C	-2.987148	-4.025216	-3.758300
O	-0.636944	2.979845	-1.836138
O	-0.487658	-2.756761	0.775351
O	-2.181190	-1.401202	2.655133
O	-3.231219	-0.536788	0.847159
O	-2.983319	0.522968	-1.184384
O	-0.560617	-0.123015	-3.481000
O	-2.082827	1.496163	-3.816617
O	-0.514924	3.694637	2.134301
O	0.613987	2.236085	0.855184
H	-0.386595	-0.079146	0.996196
H	1.186533	1.601065	-1.001859
H	1.006762	-0.833133	-2.368781
H	-0.392083	-2.690919	-3.681622
H	0.038869	-3.051081	-1.988919
H	-2.541777	-1.675356	-2.880288
H	-2.467090	-3.220192	0.430741
H	-2.187021	1.696478	0.846882
H	-3.509679	3.303451	-0.497602
H	-2.169909	4.480465	-0.429535
H	-3.021778	4.026931	1.051977
H	-4.156925	-3.407682	-0.559415
H	-4.760972	-4.451272	-1.843179
H	-4.701145	-2.696318	-2.079450
H	-1.611186	-4.662146	-0.641838
H	-1.126385	-5.119529	-2.268637
H	-2.630960	-5.750376	-1.596486
H	-3.582643	-3.251215	-4.254415
H	-3.520364	-4.976635	-3.857249
H	-2.037448	-4.121742	-4.293336
H	-0.437844	-2.817929	1.745439
H	-1.423240	3.183741	-2.370112
H	1.026162	-0.616850	0.104016

C14

C	0.185163	-0.024073	-0.069450
C	0.465058	1.461831	-0.337859
C	-0.829924	2.022824	-0.973591
C	-1.498034	0.806195	-1.654957
C	-0.772399	-0.463119	-1.188694
C	-0.061538	-0.976947	-2.465955
C	-0.570222	-2.392780	-2.662331
C	-2.040035	-2.309055	-2.221783
C	-1.942631	-1.470581	-0.914815
C	-1.854772	-2.180019	0.429682
C	-2.318841	-1.099604	1.396028
C	-3.118322	-0.499510	-0.674067
C	-1.312405	0.843233	-3.174769
C	-1.515257	2.574510	0.238099
C	-0.504780	2.750136	1.281010
C	-2.874532	3.150540	0.334424
C	-2.807490	-3.655869	-2.243790
C	-4.227684	-3.491729	-1.680325
C	-2.055877	-4.783648	-1.516620
C	-2.965374	-4.078404	-3.715349
O	-0.491253	3.052776	-1.880687
O	-0.594675	-2.690170	0.751545
O	-2.035841	-1.036083	2.558089
O	-3.102324	-0.216554	0.743764
O	-2.889790	0.660624	-1.394019
O	-0.497805	-0.151149	-3.562875
O	-1.821723	1.625112	-3.926835
O	-0.615294	3.317619	2.336435
O	0.673556	2.170028	0.876204
H	-0.272360	-0.085855	0.920880
H	1.322355	1.639370	-0.992374
H	1.027087	-0.895315	-2.418266
H	-0.427854	-2.733171	-3.690433
H	-0.015365	-3.053758	-1.985738
H	-2.558043	-1.659228	-2.946955
H	-2.618220	-2.969941	0.478670
H	-4.115758	-0.860682	-0.927534
H	-3.610817	2.367261	0.549315
H	-3.169434	3.625789	-0.607292
H	-2.903278	3.884853	1.143196
H	-4.253682	-3.250771	-0.613010
H	-4.787319	-4.423792	-1.810134
H	-4.770614	-2.705929	-2.219218
H	-1.736456	-4.526153	-0.503620
H	-1.157213	-5.069760	-2.072808
H	-2.695805	-5.670379	-1.456132
H	-3.516259	-3.324429	-4.288390
H	-3.520689	-5.020362	-3.775885
H	-1.997131	-4.235567	-4.199504
H	-0.502339	-2.618319	1.718783
H	-1.237334	3.199613	-2.486744
H	1.089495	-0.635005	-0.033176

C14Me

C	0.074999	-0.055119	0.035958
C	0.336432	1.421959	-0.276192
C	-0.976084	1.961696	-0.874029
C	-1.648699	0.734135	-1.528406
C	-0.859929	-0.510437	-1.099082
C	-0.122984	-0.941582	-2.393978
C	-0.574345	-2.369145	-2.646133
C	-2.047546	-2.359564	-2.207537
C	-1.978194	-1.580590	-0.862787
C	-1.833633	-2.359496	0.438084
C	-2.342010	-1.365481	1.469425
C	-3.193460	-0.676893	-0.556364
C	-1.518312	0.794756	-3.052024
C	-1.684587	2.505499	0.381445
C	-0.494957	2.926175	1.241449
C	-2.673342	3.586358	0.146979
C	-2.764610	-3.731517	-2.292867
C	-4.181775	-3.654026	-1.703471
C	-1.959210	-4.867650	-1.639391
C	-2.928077	-4.079323	-3.783306
O	-0.644588	2.979043	-1.777961
O	-0.540751	-2.810956	0.716230
O	-2.040548	-1.346349	2.629097
O	-3.185112	-0.493835	0.880580
O	-3.018005	0.543539	-1.189017
O	-0.593236	-0.097502	-3.459728
O	-2.132474	1.506715	-3.790040
O	-0.474085	3.735992	2.117817
O	0.604653	2.204413	0.881304
H	-0.400260	-0.109796	1.019912
H	1.161208	1.569913	-0.979768
H	0.961433	-0.818580	-2.338577
H	-0.417502	-2.662086	-3.686629
H	0.003412	-3.034981	-1.993994
H	-2.590246	-1.697540	-2.903017
H	-2.548889	-3.194354	0.449573
H	-4.176376	-1.062659	-0.828465
H	-2.162610	1.662303	0.905869
H	-3.637294	3.343116	-0.281107
H	-2.494717	4.576929	0.543091
H	-4.202106	-3.471538	-0.624339
H	-4.706597	-4.599172	-1.876726
H	-4.762470	-2.862749	-2.192554
H	-1.627301	-4.651413	-0.620668
H	-1.062969	-5.091610	-2.227013
H	-2.566239	-5.778821	-1.612758
H	-3.522087	-3.321197	-4.305354
H	-3.440989	-5.041255	-3.888245
H	-1.961980	-4.164173	-4.289402
H	-0.440853	-2.793433	1.685124
H	-1.466659	3.354977	-2.135571
H	0.984945	-0.655557	0.091568

C3OH

C	0.064797	-0.037831	0.032964
C	0.369189	1.428289	-0.290274
C	-0.984834	1.982438	-0.904075
C	-1.651442	0.739535	-1.545268
C	-0.860004	-0.499220	-1.101984
C	-0.111046	-0.938632	-2.386966
C	-0.572000	-2.364177	-2.639206
C	-2.046494	-2.347348	-2.204666
C	-1.977322	-1.567577	-0.860348
C	-1.829673	-2.343498	0.441830

C	-2.337440	-1.347991	1.472591
C	-3.190531	-0.661316	-0.552678
C	-1.527139	0.773452	-3.073785
C	-1.658826	2.546827	0.355123
C	-0.471444	2.919114	1.234866
C	-2.605075	3.713680	0.114796
C	-2.768591	-3.716744	-2.288748
C	-4.184053	-3.634298	-1.696110
C	-1.965695	-4.855347	-1.636363
C	-2.935824	-4.063899	-3.778866
O	-0.601218	2.941049	-1.780223
O	-0.533624	-2.787706	0.719165
O	-2.032251	-1.327246	2.631399
O	-3.177805	-0.474467	0.883905
O	-3.013206	0.557611	-1.187447
O	-0.557575	-0.091767	-3.455910
O	-2.174413	1.423343	-3.833804
O	-0.447941	3.682334	2.149906
O	0.640411	2.217487	0.843800
H	-0.420088	-0.062657	1.013286
H	1.192572	1.547067	-0.999978
H	0.974260	-0.829480	-2.316023
H	-0.414334	-2.657207	-3.679495
H	-0.000370	-3.034373	-1.986104
H	-2.585744	-1.684175	-2.901870
H	-2.541394	-3.181182	0.455752
H	-4.174927	-1.045291	-0.821452
H	-2.192515	1.737678	0.871678
H	-3.481699	3.377099	-0.443372
H	-2.102016	4.499789	-0.452841
H	-2.924437	4.125557	1.074378
H	-4.200945	-3.452569	-0.616696
H	-4.712705	-4.577412	-1.868630
H	-4.763069	-2.840678	-2.183289
H	-1.628891	-4.638703	-0.619240
H	-1.072741	-5.084505	-2.226935
H	-2.576696	-5.763687	-1.605502
H	-3.529752	-3.304766	-4.299431
H	-3.450321	-5.025071	-3.882881
H	-1.970885	-4.149949	-4.286979
H	-0.438821	-2.783947	1.688742
H	0.969421	-0.644641	0.109407

C10OH

C	0.079705	-0.050319	0.006359
C	0.360590	1.430152	-0.285218
C	-0.943142	1.993606	-0.892324
C	-1.618456	0.775464	-1.565549
C	-0.853564	-0.481908	-1.137752
C	-0.124498	-0.933210	-2.429046
C	-0.574699	-2.365140	-2.656187
C	-2.046894	-2.351094	-2.213978
C	-1.983629	-1.535621	-0.892290
C	-1.803857	-2.267731	0.437840
C	-2.432112	-1.304581	1.472627
C	-3.193824	-0.624139	-0.617553
C	-1.470109	0.839117	-3.087653
C	-1.654296	2.535729	0.351286
C	-0.483122	2.895036	1.259422
C	-2.605647	3.699181	0.126512
C	-2.763762	-3.725855	-2.264537
C	-4.157327	-3.647067	-1.622773
C	-1.938983	-4.861246	-1.635090
C	-2.975769	-4.083935	-3.746323
O	-0.587740	3.007177	-1.798196
O	-0.561430	-2.705141	0.764127
O	-2.266284	-1.296772	2.650704
O	-3.230545	-0.433750	0.809857
O	-2.994849	0.593467	-1.254460
O	-0.603774	-0.103587	-3.502447
O	-2.033683	1.599046	-3.821163
O	-0.475396	3.654240	2.180381
O	0.624463	2.185842	0.886860
H	-0.403598	-0.108319	0.988092
H	1.194603	1.575377	-0.978033
H	0.960276	-0.807777	-2.382885
H	-0.419246	-2.679218	-3.690906
H	0.008107	-3.015637	-1.993526
H	-2.591814	-1.707362	-2.924375
H	-2.439027	-3.171794	0.490075
H	-4.170505	-1.003526	-0.920351
H	-2.190350	1.716536	0.849435
H	-3.460555	3.380457	-0.476567
H	-2.092558	4.521919	-0.376822
H	-2.968107	4.064071	1.089665
H	-4.138527	-3.429759	-0.548868
H	-4.677398	-4.602670	-1.744415
H	-4.767294	-2.877619	-2.110318
H	-1.617616	-4.667652	-0.608368
H	-1.036944	-5.058809	-2.222675
H	-2.531182	-5.782207	-1.627992
H	-3.590690	-3.331586	-4.251818
H	-3.486247	-5.049092	-3.830131
H	-2.025930	-4.164958	-4.283305
H	-1.363299	3.237518	-2.336921
H	0.986285	-0.658255	0.064462

1B

C	0.021258	-0.024291	0.080603
C	0.335864	1.427441	-0.283397
C	-0.971689	1.972739	-0.884574
C	-1.646717	0.725371	-1.518025
C	-0.867920	-0.515604	-1.068212
C	-0.096536	-0.963773	-2.336629
C	-0.558974	-2.388780	-2.584275
C	-2.041378	-2.361969	-2.176474
C	-1.988985	-1.584222	-0.831984
C	-1.844146	-2.373154	0.473608
C	-2.322687	-1.354171	1.511696
C	-3.190629	-0.672363	-0.527330
C	-1.479825	0.764562	-3.039868
C	-1.664655	2.543763	0.358289
C	-0.480652	2.924717	1.241986
C	-2.617995	3.703101	0.119683
C	-2.777955	-3.722817	-2.281163
C	-4.202410	-3.623332	-1.714246
C	-2.009411	-4.878242	-1.619550
C	-2.919910	-4.057226	-3.776943
O	1.153292	-0.861532	0.229595
O	-0.635586	2.965552	-1.818257
O	-0.631452	-2.991567	0.742865
O	-2.040552	-1.279695	2.668618
O	-3.159158	-0.467781	0.894878
O	-3.013484	0.543097	-1.188970
O	-0.544975	-0.122193	-3.418004
O	-2.069876	1.485622	-3.792471
O	-0.454652	3.697856	2.148919
O	0.630195	2.209916	0.862402
H	-0.537472	0.008198	1.025618
H	1.169913	1.501068	-0.988711
H	0.985376	-0.849132	-2.257703
H	-0.380956	-2.693189	-3.617981
H	-0.002639	-3.052132	-1.911658
H	-2.562308	-1.693432	-2.882781
H	-2.597596	-3.168418	0.488130
H	-4.177519	-1.052079	-0.793846
H	-2.196345	1.735956	0.880887
H	-3.479936	3.368915	-0.464456
H	-2.111941	4.513336	-0.409958
H	-2.969215	4.089298	1.078648
H	-4.233424	-3.452043	-0.633520
H	-4.741757	-4.556992	-1.904092
H	-4.761385	-2.816453	-2.203392
H	-1.732161	-4.691823	-0.579703
H	-1.084165	-5.089482	-2.165747
H	-2.620520	-5.786550	-1.652808
H	-3.487418	-3.283647	-4.305986
H	-3.450904	-5.007284	-3.897939
H	-1.946083	-4.160378	-4.264818
H	0.098133	-2.343385	0.725016
H	1.698536	-0.507608	0.950865
H	-1.412706	3.159492	-2.369592

C1

C	0.072942	-0.019652	-0.055772
C	0.323496	1.442309	-0.205693
C	-0.955553	1.971331	-0.876269
C	-1.591591	0.715357	-1.556328
C	-0.799785	-0.540354	-1.147161
C	-0.096864	-1.001121	-2.460819
C	-0.591724	-2.416522	-2.695541
C	-2.049571	-2.377359	-2.208606
C	-1.923861	-1.607606	-0.868416
C	-1.729364	-2.380248	0.439262
C	-2.033484	-1.285225	1.459286
C	-3.093514	-0.681928	-0.507670
C	-1.449841	0.789779	-3.077850
C	-1.737105	2.523680	0.324187
C	-0.651059	2.817184	1.349515
C	-2.622939	3.726307	0.041541
C	-2.807388	-3.728932	-2.279762
C	-4.211423	-3.600246	-1.670636
C	-2.045587	-4.891094	-1.624481
C	-2.995315	-4.074795	-3.767626
O	0.991571	-0.807736	0.565201
O	-0.578939	2.960847	-1.797422
O	-0.544679	-3.076400	0.624479
O	-1.579447	-1.137423	2.552749
O	-2.950312	-0.437692	0.904160
O	-2.956303	0.503888	-1.225965
O	-0.575959	-0.136400	-3.508248
O	-2.020955	1.557382	-3.799073
O	-0.710443	3.529132	2.304073
O	0.482203	2.102584	1.046346
H	1.212560	1.671634	-0.810144
H	0.990388	-0.902632	-2.430884
H	-0.474696	-2.712884	-3.740351
H	-0.008779	-3.092730	-2.059794
H	-2.598386	-1.699988	-2.884846
H	-2.544493	-3.107614	0.536135
H	-4.100401	-1.059221	-0.688521
H	-2.337398	1.718021	0.762654
H	-3.438035	3.445819	-0.632031
H	-2.044681	4.535746	-0.409709
H	-3.050102	4.090571	0.978045
H	-4.200314	-3.427386	-0.589688
H	-4.774760	-4.523738	-1.839651
H	-4.770212	-2.782932	-2.142499
H	-1.788106	-4.718655	-0.577492
H	-1.111880	-5.096629	-2.157959
H	-2.657167	-5.798251	-1.678618
H	-3.546310	-3.287051	-4.293428
H	-3.562885	-5.006118	-3.865467
H	-2.037149	-4.219180	-4.275601
H	0.149028	-2.444673	0.884335
H	1.374961	-0.298615	1.301931
H	-1.337007	3.162520	-2.372254

C2

C	0.047823	-0.122513	0.137220
C	0.165302	1.366025	-0.006633
C	-0.977371	1.954857	-0.793482
C	-1.659479	0.715063	-1.434604
C	-0.866229	-0.535683	-1.032899
C	-0.125204	-0.944180	-2.332962
C	-0.575909	-2.369942	-2.597890
C	-2.051561	-2.363731	-2.163114
C	-1.978873	-1.614938	-0.803126
C	-1.801994	-2.437020	0.477754
C	-2.289530	-1.463876	1.558266
C	-3.176487	-0.715825	-0.448531
C	-1.531948	0.793704	-2.958247
C	-1.753573	2.675394	0.320939
C	-0.631325	3.031862	1.288026
C	-2.590341	3.870631	-0.098212
C	-2.781471	-3.726642	-2.284469
C	-4.193668	-3.650761	-1.684101
C	-1.990059	-4.893166	-1.670757
C	-2.955091	-4.024726	-3.784641
O	1.246321	-0.884599	0.093756
O	-0.458557	2.853706	-1.746232
O	-0.573790	-3.042299	0.713956
O	-2.026581	-1.451709	2.721929
O	-3.116031	-0.545399	0.976410
O	-3.016638	0.518222	-1.077440
O	-0.626049	-0.095379	-3.385832
O	-2.131632	1.547182	-3.672022
O	-0.568469	3.895916	2.099605
O	0.404539	2.106280	1.117309
H	-0.433949	-0.294532	1.111814
H	0.954237	-0.804558	-2.283035
H	-0.415177	-2.653695	-3.640287
H	-0.000274	-3.040746	-1.949308
H	-2.588970	-1.683986	-2.845515
H	-2.537487	-3.248716	0.478801
H	-4.167601	-1.091805	-0.705282
H	-2.377391	1.933186	0.840485
H	-3.389203	3.550696	-0.773614
H	-1.969790	4.617323	-0.599080
H	-3.039846	4.337114	0.780884
H	-4.202561	-3.505934	-0.599067
H	-4.731586	-4.582942	-1.885107
H	-4.767935	-2.835779	-2.141065
H	-1.686819	-4.731215	-0.634018
H	-1.077950	-5.084585	-2.245630
H	-2.596414	-5.804382	-1.710919
H	-3.541228	-3.243275	-4.280739
H	-3.480421	-4.976349	-3.917631
H	-1.991824	-4.106216	-4.296917
H	0.158928	-2.403305	0.638159
H	1.854574	-0.535631	0.764318
H	-1.183444	3.135072	-2.331268

C6

C	0.030686	-0.035547	0.024447
C	0.343626	1.422622	-0.309930
C	-0.972985	1.990649	-0.871015
C	-1.650468	0.768493	-1.546798
C	-0.892524	-0.492168	-1.113717
C	-0.204623	-0.954512	-2.387404
C	-0.555692	-2.389470	-2.615566
C	-2.049514	-2.383911	-2.188915
C	-2.017730	-1.560437	-0.865568
C	-1.888747	-2.315769	0.461651
C	-2.384071	-1.274121	1.467945
C	-3.222602	-0.636583	-0.599754
C	-1.446433	0.841178	-3.065696
C	-1.647140	2.514135	0.402267
C	-0.446186	2.871868	1.274338
C	-2.608540	3.677635	0.222655
C	-2.764860	-3.757795	-2.256489
C	-4.200591	-3.650362	-1.718933
C	-1.996231	-4.880448	-1.541891
C	-2.876209	-4.146704	-3.741651
O	1.154735	-0.886909	0.120735
O	-0.644345	3.021613	-1.765110
O	-0.683455	-2.932846	0.760783
O	-2.119138	-1.163835	2.625652
O	-3.227226	-0.416559	0.817876
O	-3.027011	0.577292	-1.265916
O	-0.562413	-0.120767	-3.445240
O	-1.964634	1.602080	-3.825604
O	-0.406660	3.617041	2.203938
O	0.659673	2.176624	0.849435
H	-0.506310	-0.025787	0.983488
H	1.164627	1.505728	-1.029070
H	-0.389615	-2.701348	-3.647765
H	0.022929	-3.025943	-1.937052
H	-2.576099	-1.743721	-2.917278
H	-2.645707	-3.107784	0.485407
H	-4.204268	-1.012889	-0.890436
H	-2.167059	1.688665	0.908162
H	-3.480885	3.361878	-0.356285
H	-2.115173	4.509300	-0.285440
H	-2.942563	4.027377	1.201521
H	-4.253308	-3.432471	-0.647578
H	-4.726637	-4.597373	-1.877664

H	-4.758869	-2.871427	-2.252368
H	-1.760139	-4.666830	-0.497589
H	-1.048067	-5.083553	-2.050595
H	-2.587279	-5.801892	-1.577295
H	-3.429016	-3.391512	-4.311492
H	-3.408638	-5.098605	-3.838727
H	-1.892522	-4.272175	-4.203823
H	0.063155	-2.309997	0.672276
H	1.731651	-0.546715	0.823847
H	-1.430368	3.256980	-2.285823

C7

C	0.011761	-0.032181	0.107611
C	0.343096	1.416028	-0.254851
C	-0.953411	1.973434	-0.866459
C	-1.628298	0.733363	-1.516421
C	-0.873390	-0.517604	-1.048655
C	-0.085721	-0.987952	-2.299606
C	-0.660014	-2.319325	-2.612962
C	-2.082061	-2.361899	-2.150476
C	-2.014156	-1.564593	-0.813112
C	-1.895713	-2.336764	0.503787
C	-2.379516	-1.296200	1.519807
C	-3.208304	-0.634610	-0.541672
C	-1.409273	0.783873	-3.031380
C	-1.654893	2.544274	0.371859
C	-0.475436	2.916251	1.265937
C	-2.598772	3.710508	0.128918
C	-2.766645	-3.752420	-2.230584
C	-4.200743	-3.688366	-1.685906
C	-1.958677	-4.854240	-1.529497
C	-2.870402	-4.121043	-3.721196
O	1.135263	-0.877867	0.266331
O	-0.600059	2.970828	-1.788854
O	-0.697954	-2.967096	0.796838
O	-2.119030	-1.206879	2.680318
O	-3.199937	-0.411215	0.876260
O	-3.004396	0.566893	-1.221151
O	-0.424109	-0.060661	-3.375963
O	-1.992180	1.494555	-3.799818
O	-0.452257	3.687690	2.174373
O	0.634237	2.195868	0.893641
H	-0.554139	0.005008	1.048048
H	1.183650	1.481052	-0.953342
H	0.998152	-0.957217	-2.185818
H	-0.232649	-2.973265	-3.361702
H	-2.672543	-1.715114	-2.835224
H	-2.662373	-3.119278	0.521449
H	-4.193729	-1.010286	-0.822697
H	-2.196278	1.739387	0.888511
H	-3.457635	3.384018	-0.464014
H	-2.083019	4.519815	-0.392738
H	-2.955983	4.095118	1.086316
H	-4.253099	-3.471644	-0.614438
H	-4.698818	-4.650896	-1.840977
H	-4.785172	-2.926059	-2.215355
H	-1.827812	-4.695427	-0.457878
H	-0.956711	-4.933748	-1.963889
H	-2.461272	-5.817030	-1.671664
H	-3.431787	-3.364329	-4.280462
H	-3.390251	-5.078151	-3.832454
H	-1.885635	-4.226398	-4.186424
H	0.051084	-2.346351	0.720046
H	1.672204	-0.532663	0.997911
H	-1.370331	3.177259	-2.345099

C8

C	0.035988	0.003702	0.113171
C	0.318466	1.445712	-0.312415
C	-1.006742	1.938082	-0.922921
C	-1.666425	0.651424	-1.491722
C	-0.842270	-0.548143	-1.013330
C	-0.066894	-0.999901	-2.278874
C	-0.474096	-2.448779	-2.496034
C	-1.884593	-2.508265	-1.973672
C	-1.927076	-1.641705	-0.740820
C	-1.760552	-2.407593	0.589739
C	-2.240827	-1.373907	1.608019
C	-3.155761	-0.754205	-0.439920
C	-1.538830	0.632403	-3.016267
C	-1.698456	2.549092	0.301560
C	-0.514780	2.988882	1.157580
C	-2.676125	3.678855	0.023052
C	-2.779029	-3.683372	-2.296519
C	-4.196857	-3.537177	-1.727944
C	-2.137564	-4.977907	-1.749483
C	-2.906973	-3.809304	-3.826566
O	1.182017	-0.806391	0.296946
O	-0.701329	2.893249	-1.905087
O	-0.534379	-3.002417	0.834766
O	-1.935251	-1.259180	2.756176
O	-3.099514	-0.519133	0.980237
O	-3.017871	0.453145	-1.118971
O	-0.558524	-0.210282	-3.377518
O	-2.180691	1.291654	-3.782759
O	-0.496240	3.799747	2.031256
O	0.606315	2.280399	0.797723
H	-0.525382	0.065494	1.055249
H	1.144450	1.508489	-1.028153
H	1.010416	-0.841574	-2.213786
H	-0.357708	-2.739078	-3.543057
H	0.181875	-3.091915	-1.885395
H	-2.502550	-3.215784	0.609430
H	-4.138216	-1.158673	-0.673785
H	-2.210068	1.753600	0.861983
H	-3.535885	3.303942	-0.539270
H	-2.190375	4.475976	-0.544271
H	-3.027432	4.098248	0.967933
H	-4.213693	-3.458087	-0.635553
H	-4.786863	-4.419896	-1.993542
H	-4.703419	-2.662735	-2.150430
H	-1.989136	-4.939097	-0.665538
H	-1.160496	-5.153603	-2.210946
H	-2.777184	-5.837308	-1.979226
H	-3.294985	-2.883313	-4.263225
H	-3.594909	-4.624133	-4.075575
H	-1.945876	-4.033926	-4.297451
H	0.173900	-2.330496	0.838525
H	1.727591	-0.404525	0.992332
H	-1.492974	3.056404	-2.445625

C8tBu

C	0.027151	-0.006252	0.085114
C	0.337764	1.448706	-0.269207
C	-0.972111	1.994040	-0.866049
C	-1.642816	0.749065	-1.508870
C	-0.859627	-0.492261	-1.067210
C	-0.086627	-0.929023	-2.340551
C	-0.543361	-2.353995	-2.595675
C	-2.021042	-2.336435	-2.179912
C	-1.977398	-1.565460	-0.834248
C	-1.835790	-2.359657	0.468074
C	-2.319075	-1.346664	1.509490
C	-3.183657	-0.659931	-0.529049
C	-1.475845	0.800133	-3.030344
C	-1.666495	2.553476	0.381318
C	-0.483419	2.931880	1.267442
C	-2.623626	3.711525	0.151718
C	-2.732846	-3.710010	-2.305523
C	-4.179045	-3.631486	-1.769683
C	-1.970485	-4.862106	-1.627266
C	-2.816674	-4.016931	-3.776429
O	1.159712	-0.842961	0.230905
O	-0.639572	2.994992	-1.792213
O	-0.624609	-2.979953	0.737557
O	-2.039302	-1.275546	2.667133
O	-3.158073	-0.461643	0.894129
O	-3.009707	0.559552	-1.183580
O	-0.540181	-0.081857	-3.415629
O	-2.068606	1.525384	-3.776987
O	-0.459818	3.698207	2.180194
O	0.629914	2.224155	0.881898
H	-0.533187	0.019306	1.029562
H	1.171166	1.530231	-0.974437

H	0.994630	-0.808832	-2.261230
H	-0.385260	-2.664060	-3.631812
H	0.019962	-3.018360	-1.929822
H	-2.550568	-1.671552	-2.883316
H	-2.591293	-3.153770	0.475177
H	-4.167855	-1.043882	-0.800950
H	-2.195522	1.740209	0.898048
H	-3.484862	3.378973	-0.434383
H	-2.120442	4.527205	-0.372260
H	-2.975454	4.089479	1.113752
H	-4.227188	-3.466486	-0.687000
H	-4.701515	-4.569974	-1.978048
H	-4.733727	-2.826730	-2.265621
H	-1.742475	-4.684196	-0.574697
H	-1.022091	-5.049168	-2.139850
H	-2.566952	-5.777849	-1.698421
H	-3.155024	-3.255197	-4.472023
H	-2.831491	-5.048186	-4.111294
H	0.104719	-2.331320	0.726135
H	1.711341	-0.483291	0.944453
H	-1.416833	3.189049	-2.343331

C10

C	0.024643	-0.089375	-0.080001
C	0.416325	1.362552	-0.359694
C	-0.891371	2.036842	-0.836064
C	-1.738099	0.871139	-1.423608
C	-0.959394	-0.415752	-1.192881
C	-0.322698	-0.752012	-2.553884
C	-0.630194	-2.223920	-2.774745
C	-2.054636	-2.382759	-2.225248
C	-2.063849	-1.483186	-0.937665
C	-1.987650	-2.079890	0.420640
C	-3.117175	-1.691059	1.241663
C	-3.371961	-0.636305	-0.783094
C	-1.828294	0.973986	-2.953543
C	-1.424733	2.636797	0.469127
C	-0.155460	2.852904	1.284798
C	-2.264995	3.895983	0.333038
C	-2.625607	-3.817715	-2.156037
C	-4.084456	-3.758629	-1.683749
C	-1.832342	-4.764100	-1.245095
C	-2.602812	-4.395949	-3.580548
O	1.080338	-1.034260	-0.090826
O	-0.548080	3.004992	-1.792566
O	-0.935659	-2.674188	0.983221
O	-3.360195	-1.945093	2.389441
O	-3.962798	-0.924540	0.451032
O	-2.998015	0.732691	-0.815471
O	-1.027077	0.051173	-3.523082
O	-2.502834	1.747311	-3.570006
O	0.006609	3.574340	2.219949
O	0.845795	2.044495	0.806639
H	-0.493824	-0.111104	0.891528
H	1.201404	1.435188	-1.118911
H	0.739423	-0.507181	-2.624237
H	-0.536564	-2.502351	-3.827111
H	0.093368	-2.810192	-2.193949
H	-2.705534	-1.850904	-2.936758
H	-4.111856	-0.828967	-1.565434
H	-2.005297	1.868282	0.997596
H	-3.193792	3.674607	-0.200453
H	-1.712592	4.671297	-0.203079
H	-2.511915	4.278984	1.325131
H	-4.176833	-3.341484	-0.675322
H	-4.514969	-4.765080	-1.661189
H	-4.694604	-3.151735	-2.363680
H	-1.937061	-4.520906	-0.186251
H	-0.765244	-4.771550	-1.495225
H	-2.201934	-5.785736	-1.383674
H	-3.090592	-3.722213	-4.294439
H	-3.134494	-5.352688	-3.603263
H	-1.579925	-4.578703	-3.925854
H	-0.128425	-2.518310	0.452860
H	1.710814	-0.800301	0.609343
H	-1.361374	3.336251	-2.210256

C12

C	0.038399	-0.018280	0.084080
C	0.335849	1.437192	-0.277987
C	-0.971733	1.962961	-0.897327
C	-1.626457	0.702701	-1.527649
C	-0.825304	-0.525581	-1.072665
C	-0.059707	-0.975134	-2.342388
C	-0.527320	-2.401679	-2.586748
C	-2.005978	-2.362674	-2.170096
C	-1.938287	-1.602767	-0.816974
C	-1.801808	-2.403697	0.483848
C	-2.370079	-1.435062	1.528081
C	-3.100724	-0.690957	-0.500003
C	-1.470198	0.734923	-3.047778
C	-1.684764	2.534796	0.334738
C	-0.515306	2.932862	1.230452
C	-2.648611	3.681509	0.078257
C	-2.781982	-3.697036	-2.273521
C	-4.187802	-3.543643	-1.671424
C	-2.027259	-4.880139	-1.645395
C	-2.968732	-4.009127	-3.768892
O	1.178247	-0.837720	0.255031
O	-0.638193	2.950949	-1.835574
O	-0.580846	-2.985290	0.790102
O	-2.173375	-1.382643	2.700076
O	-3.214045	-0.549608	0.880364
O	-2.996398	0.503217	-1.157092
O	-0.516651	-0.134019	-3.422292
O	-2.072959	1.444353	-3.801012
O	-0.508680	3.710092	2.133859
O	0.606855	2.226550	0.868433
H	-0.544954	0.003997	1.017874
H	1.176405	1.519146	-0.974636
H	1.022678	-0.859219	-2.271164
H	-0.357613	-2.706194	-3.621921
H	0.031140	-3.068389	-1.918537
H	-2.522684	-1.676304	-2.860846
H	-2.527017	-3.223174	0.456612
H	-2.214088	1.725509	0.857711
H	-3.500596	3.334026	-0.512634
H	-2.145727	4.494244	-0.450587
H	-3.014410	4.069539	1.030974
H	-4.190062	-3.410619	-0.585427
H	-4.783451	-4.436571	-1.888282
H	-4.703030	-2.680061	-2.107861
H	-1.692585	-4.704804	-0.620014
H	-1.136424	-5.122860	-2.234783
H	-2.670288	-5.766699	-1.646969
H	-3.542938	-3.222210	-4.270037
H	-3.512761	-4.951954	-3.888406
H	-2.009401	-4.114397	-4.285013
H	0.137820	-2.326147	0.754407
H	1.720999	-0.459648	0.965974
H	-1.415739	3.141081	-2.387852

C14

C	0.088061	0.010200	0.017081
C	0.446012	1.454920	-0.345070
C	-0.843178	2.017013	-0.978567
C	-1.526400	0.780616	-1.622755
C	-0.803020	-0.476412	-1.129103
C	-0.033775	-0.993047	-2.370599
C	-0.545219	-2.407178	-2.579561
C	-2.029871	-2.317097	-2.189391
C	-1.968246	-1.492618	-0.873215
C	-1.878557	-2.228497	0.467241
C	-2.337594	-1.147696	1.453576
C	-3.138362	-0.524699	-0.627952
C	-1.311376	0.787017	-3.140466
C	-1.497238	2.610070	0.232383
C	-0.459363	2.820867	1.240966
C	-2.861242	3.167995	0.352054
C	-2.810375	-3.655614	-2.253602
C	-4.239920	-3.484247	-1.717812
C	-2.094419	-4.807897	-1.530614
C	-2.937442	-4.046213	-3.736952
O	1.194865	-0.853616	0.195418

O	-0.506518	3.020583	-1.912736
O	-0.696422	-2.881512	0.787107
O	-2.089779	-1.048963	2.615914
O	-3.110703	-0.244455	0.779564
O	-2.910867	0.644036	-1.352773
O	-0.433103	-0.166640	-3.483348
O	-1.837408	1.531911	-3.918166
O	-0.529625	3.427736	2.275657
O	0.709792	2.206321	0.827945
H	-0.482249	0.073452	0.953106
H	1.304101	1.522022	-1.020519
H	1.050472	-0.916555	-2.278568
H	-0.367134	-2.751544	-3.600653
H	-0.018447	-3.066837	-1.879863
H	-2.519924	-1.657018	-2.925291
H	-2.662411	-2.993351	0.500733
H	-4.135520	-0.882274	-0.887876
H	-3.575545	2.374417	0.602000
H	-3.188535	3.615011	-0.592633
H	-2.882716	3.919743	1.144632
H	-4.282773	-3.267918	-0.645642
H	-4.808503	-4.405810	-1.879485
H	-4.761906	-2.679256	-2.249199
H	-1.833171	-4.589183	-0.492910
H	-1.165506	-5.070994	-2.047300
H	-2.734601	-5.696537	-1.541775
H	-3.464526	-3.274188	-4.308492
H	-3.502625	-4.979546	-3.829904
H	-1.959437	-4.206726	-4.200339
H	0.061121	-2.267631	0.747421
H	1.743217	-0.493115	0.911376
H	-1.251836	3.148256	-2.524688

C14Me

C	0.004087	-0.036159	0.104974
C	0.312395	1.416369	-0.257756
C	-0.991837	1.952565	-0.865745
C	-1.668629	0.707489	-1.496216
C	-0.878524	-0.527585	-1.050220
C	-0.099954	-0.958117	-2.321003
C	-0.557530	-2.382558	-2.584503
C	-2.040289	-2.367313	-2.176643
C	-1.991272	-1.605904	-0.822763
C	-1.838160	-2.409705	0.472914
C	-2.316580	-1.404906	1.524648
C	-3.197041	-0.704381	-0.502998
C	-1.513708	0.749734	-3.019437
C	-1.695552	2.527274	0.380637
C	-0.502768	2.949669	1.236732
C	-2.668989	3.617358	0.124595
C	-2.770728	-3.730178	-2.297306
C	-4.194436	-3.644464	-1.726430
C	-1.995833	-4.890228	-1.651437
C	-2.913712	-4.046324	-3.796883
O	1.139452	-0.868614	0.254555
O	-0.652776	2.947606	-1.789199
O	-0.621291	-3.024586	0.730886
O	-2.028556	-1.342300	2.680968
O	-3.157665	-0.513350	0.921981
O	-3.030957	0.519551	-1.151083
O	-0.544632	-0.106927	-3.394377
O	-2.136788	1.435654	-3.774415
O	-0.468216	3.767948	2.102018
O	0.598156	2.211121	0.883516
H	-0.557186	-0.008748	1.048604
H	1.148850	1.492650	-0.960268
H	0.981376	-0.843162	-2.234684
H	-0.378598	-2.673916	-3.621776
H	0.000946	-3.051967	-1.919683
H	-2.564776	-1.692992	-2.874879
H	-2.587572	-3.208937	0.480472
H	-4.183295	-1.087979	-0.766388
H	-2.189427	1.698884	0.915430
H	-3.641785	3.375645	-0.283424
H	-2.466736	4.619073	0.478762
H	-4.223589	-3.487899	-0.643381
H	-4.730274	-4.577803	-1.927617
H	-4.757824	-2.833439	-2.203501
H	-1.718665	-4.715971	-0.609428
H	-1.070026	-5.089698	-2.201051
H	-2.602496	-5.801054	-1.695929
H	-3.485512	-3.268645	-4.315173
H	-3.440533	-4.997237	-3.929031
H	-1.940201	-4.138643	-4.287513
H	0.102354	-2.369556	0.727880
H	1.684812	-0.511467	0.974027
H	-1.471664	3.314048	-2.164260

C1OH

C	0.062766	-0.049342	0.041998
C	0.346493	1.458861	-0.272979
C	-0.974792	1.992038	-0.869974
C	-1.636143	0.767142	-1.545041
C	-0.873263	-0.484214	-1.110917
C	-0.115865	-0.932503	-2.382688
C	-0.563036	-2.362843	-2.616280
C	-2.042972	-2.350588	-2.201170
C	-1.999329	-1.542191	-0.872170
C	-1.871845	-2.290963	0.449981
C	-2.411061	-1.280656	1.449927
C	-3.217041	-0.628443	-0.608233
C	-1.461473	0.830825	-3.066368
C	-1.681650	2.519660	0.383828
C	-0.507527	2.898030	1.279517
C	-2.652518	3.669142	0.168792
C	-2.754282	-3.726250	-2.264897
C	-4.179312	-3.638984	-1.696010
C	-1.952747	-4.843975	-1.575776
C	-2.896892	-4.108194	-3.749132
O	1.252858	-0.700569	0.108801
O	-0.639092	3.017666	-1.767308
O	-0.575688	-2.715373	0.749831
O	-2.137538	-1.231443	2.614963
O	-3.248262	-0.426959	0.823001
O	-3.015534	0.586018	-1.250920
O	-0.579248	-0.097488	-3.464139
O	-2.019866	1.593307	-3.803110
O	-0.497309	3.650646	2.203638
O	0.615380	2.213550	0.884221
H	-0.441004	-0.050869	1.020303
H	1.179383	1.564391	-0.972985
H	0.965100	-0.808949	-2.307523
H	-0.388590	-2.676829	-3.647762
H	0.010684	-3.008110	-1.940362
H	-2.577296	-1.705148	-2.918524
H	-2.575054	-3.135383	0.468133
H	-4.193782	-1.009899	-0.906606
H	-2.201182	1.692491	0.886348
H	-3.507889	3.335876	-0.425295
H	-2.156827	4.497920	-0.341383
H	-3.010743	4.028148	1.135716
H	-4.215056	-3.425760	-0.622827
H	-4.698076	-4.590874	-1.849125
H	-4.756977	-2.864532	-2.214903
H	-1.632347	-4.603387	-0.558862
H	-1.049010	-5.077982	-2.147653
H	-2.557023	-5.756395	-1.534720
H	-3.486839	-3.364492	-4.296013
H	-3.404847	-5.074242	-3.838923
H	-1.923791	-4.200815	-4.240214
H	-0.472137	-2.674219	1.717595
H	-1.397757	3.192780	-2.350090

C3OH

C	-0.021398	-0.011821	0.093062
C	0.310799	1.431713	-0.294183
C	-1.041421	1.963435	-0.903699
C	-1.706875	0.694377	-1.508271
C	-0.896354	-0.525371	-1.053103
C	-0.112344	-0.955730	-2.320486
C	-0.571299	-2.380833	-2.584589
C	-2.051982	-2.370549	-2.168097
C	-1.996548	-1.612989	-0.812399
C	-1.823822	-2.418130	0.479840

C	-2.284432	-1.411957	1.537594
C	-3.203346	-0.719674	-0.474951
C	-1.572907	0.706824	-3.037379
C	-1.704731	2.561247	0.347493
C	-0.509344	2.952040	1.209731
C	-2.647791	3.726127	0.084360
C	-2.779534	-3.735156	-2.287445
C	-4.198853	-3.654828	-1.705250
C	-1.997795	-4.895440	-1.650661
C	-2.932265	-4.046539	-3.787002
O	1.114950	-0.833198	0.272785
O	-0.670706	2.903006	-1.808460
O	-0.602466	-3.031826	0.719165
O	-1.968108	-1.342791	2.686433
O	-3.137982	-0.523691	0.949365
O	-3.055509	0.503377	-1.128344
O	-0.547626	-0.102232	-3.391652
O	-2.246761	1.312349	-3.810580
O	-0.471222	3.729584	2.109682
O	0.606005	2.240323	0.818139
H	-0.587398	0.048955	1.031767
H	1.138083	1.480898	-1.008979
H	0.969297	-0.846555	-2.227318
H	-0.398388	-2.667221	-3.624218
H	-0.008888	-3.052910	-1.925906
H	-2.584060	-1.696459	-2.860857
H	-2.572726	-3.217620	0.497189
H	-4.191818	-1.110012	-0.718904
H	-2.240924	1.766391	0.884866
H	-3.526268	3.378248	-0.463712
H	-2.144745	4.498692	-0.501466
H	-2.964779	4.158672	1.035514
H	-4.219219	-3.505597	-0.620863
H	-4.734964	-4.587692	-1.907844
H	-4.767128	-2.841615	-2.172615
H	-1.717780	-4.725633	-0.608639
H	-1.073445	-5.089345	-2.204618
H	-2.601731	-5.807960	-1.697229
H	-3.509453	-3.268463	-4.298604
H	-3.457735	-4.998280	-3.918512
H	-1.961952	-4.134859	-4.284650
H	0.113356	-2.369147	0.734068
H	1.641871	-0.469663	1.003020

C10OH

C	0.085033	-0.012752	0.035984
C	0.336495	1.468226	-0.264574
C	-0.968473	2.014876	-0.875608
C	-1.633264	0.783650	-1.540551
C	-0.862179	-0.462015	-1.104827
C	-0.112775	-0.908507	-2.384177
C	-0.548933	-2.345141	-2.605618
C	-2.025730	-2.345088	-2.181243
C	-1.982639	-1.524407	-0.858347
C	-1.836654	-2.290402	0.457779
C	-2.458124	-1.282258	1.509451
C	-3.202346	-0.619824	-0.600411
C	-1.479161	0.839235	-3.063658
C	-1.686317	2.564069	0.361567
C	-0.518064	2.934670	1.270294
C	-2.641738	3.721851	0.126268
C	-2.732854	-3.724519	-2.244174
C	-4.150907	-3.646401	-1.658153
C	-1.927988	-4.850923	-1.574488
C	-2.887361	-4.095681	-3.730146
O	1.299260	-0.706170	0.078434
O	-0.623959	3.022644	-1.791279
O	-0.609148	-2.671948	0.847800
O	-2.303465	-1.293890	2.684780
O	-3.235638	-0.421412	0.831855
O	-3.012403	0.594012	-1.235783
O	-0.597187	-0.089833	-3.466495
O	-2.055962	1.586561	-3.800424
O	-0.520115	3.698243	2.187894
O	0.592995	2.230831	0.904058
H	-0.424954	-0.058483	1.010193
H	1.181992	1.585235	-0.949380
H	0.966905	-0.772592	-2.317357
H	-0.377804	-2.668205	-3.634858
H	0.037332	-2.983517	-1.932486
H	-2.566452	-1.704382	-2.897575
H	-2.543937	-3.133554	0.531001
H	-4.178217	-1.009535	-0.891597
H	-2.219606	1.746522	0.865529
H	-3.494478	3.396759	-0.476632
H	-2.130106	4.542482	-0.381789
H	-3.007201	4.092550	1.086074
H	-4.174028	-3.432921	-0.583995
H	-4.666121	-4.601404	-1.802926
H	-4.740457	-2.875681	-2.168746
H	-1.668858	-4.660000	-0.529650
H	-0.993879	-5.036535	-2.113971
H	-2.508929	-5.778712	-1.602762
H	-3.473907	-3.342943	-4.267969
H	-3.404063	-5.056494	-3.824186
H	-1.917994	-4.192481	-4.227819
H	1.114814	-1.574979	0.470054
H	-1.401033	3.237693	-2.334278

1C

C	0.012250	-0.016594	0.119105
C	0.333106	1.429579	-0.261900
C	-0.964754	1.965908	-0.890065
C	-1.635894	0.708943	-1.509768
C	-0.852051	-0.523070	-1.042733
C	-0.060000	-0.964852	-2.286792
C	-0.527993	-2.393220	-2.561396
C	-2.028846	-2.355848	-2.184828
C	-1.976426	-1.600337	-0.830756
C	-1.825477	-2.404756	0.465529
C	-2.306923	-1.396412	1.513476
C	-3.176240	-0.691177	-0.516781
C	-1.465890	0.729399	-3.031424
C	-1.674350	2.560035	0.331870
C	-0.502911	2.951777	1.227769
C	-2.619946	3.718659	0.060407
C	-2.784060	-3.708124	-2.304766
C	-4.184730	-3.619877	-1.676695
C	-1.984605	-4.876388	-1.707144
C	-3.005507	-4.009977	-3.799293
O	1.142401	-0.848072	0.300686
O	-0.612221	2.936940	-1.840378
O	-0.605018	-3.011859	0.725242
O	-2.025983	-1.336615	2.671331
O	-3.135712	-0.495186	0.905762
O	-3.000131	0.527288	-1.175267
O	-0.497536	-0.141004	-3.388446
O	-2.070648	1.411104	-3.805291
O	-0.489854	3.738920	2.122603
O	0.611349	2.228695	0.876083
O	-0.205087	-2.823154	-3.851771
H	-0.565870	0.030076	1.051611
H	1.177690	1.491795	-0.955754
H	1.022975	-0.865269	-2.207458
H	-0.009402	-3.056457	-1.858859
H	-2.527932	-1.671867	-2.894381
H	-2.567140	-3.209885	0.473447
H	-4.164122	-1.068389	-0.781105
H	-2.216790	1.761756	0.858142
H	-3.472188	3.380150	-0.535561
H	-2.102195	4.522936	-0.467485
H	-2.988299	4.117455	1.007657
H	-4.183577	-3.464473	-0.593578
H	-4.725043	-4.553808	-1.862306
H	-4.766363	-2.811362	-2.136015
H	-1.666881	-4.713706	-0.673768
H	-1.084190	-5.062522	-2.302105
H	-2.591797	-5.787317	-1.737770
H	-3.591139	-3.216044	-4.278063
H	-3.565810	-4.945351	-3.904036
H	-2.058072	-4.114859	-4.329606
H	0.110653	-2.349068	0.762346
H	1.680099	-0.477260	1.019171
H	-1.399270	3.174648	-2.358995

H	-0.509340	-2.136197	-4.467904

C1

C	0.066861	-0.014806	-0.011077
C	0.323403	1.444802	-0.180445
C	-0.943919	1.965749	-0.877956
C	-1.571564	0.702404	-1.551023
C	-0.779936	-0.546315	-1.119457
C	-0.048965	-1.005002	-2.403901
C	-0.549772	-2.424363	-2.663611
C	-2.030120	-2.371375	-2.217170
C	-1.912205	-1.618525	-0.868659
C	-1.720046	-2.400123	0.434436
C	-2.038330	-1.312332	1.459697
C	-3.081723	-0.692878	-0.510918
C	-1.415681	0.758724	-3.071806
C	-1.744068	2.532142	0.302991
C	-0.672420	2.844073	1.338231
C	-2.630888	3.727077	-0.007600
C	-2.808367	-3.713558	-2.301231
C	-4.186410	-3.594544	-1.630690
C	-2.015015	-4.887522	-1.708734
C	-3.078512	-4.029199	-3.784502
O	0.979668	-0.795307	0.626371
O	-0.552105	2.940925	-1.807920
O	-0.524824	-3.079043	0.619722
O	-1.599204	-1.177863	2.560720
O	-2.943388	-0.453654	0.901212
O	-2.937366	0.493744	-1.228052
O	-0.512922	-0.161141	-3.477098
O	-1.990879	1.496540	-3.817082
O	-0.747070	3.569553	2.281208
O	0.465075	2.125644	1.061962
O	-0.291917	-2.848701	-3.971070
H	1.222354	1.660462	-0.775395
H	1.038945	-0.921964	-2.369019
H	-0.007572	-3.098579	-1.990430
H	-2.551595	-1.679920	-2.903254
H	-2.522358	-3.141269	0.523567
H	-4.088054	-1.066978	-0.698075
H	-2.346523	1.729022	0.743802
H	-3.434865	3.435492	-0.689860
H	-2.049565	4.535096	-0.457875
H	-3.073845	4.100372	0.917885
H	-4.143518	-3.438119	-0.548639
H	-4.751645	-4.517568	-1.795550
H	-4.765903	-2.774659	-2.072382
H	-1.688684	-4.724235	-0.678330
H	-1.122088	-5.087379	-2.310390
H	-2.633079	-5.791467	-1.730555
H	-3.655984	-3.226270	-4.258363
H	-3.666395	-4.950282	-3.860506
H	-2.149608	-4.166192	-4.339468
H	0.152482	-2.445515	0.915782
H	1.354216	-0.281423	1.364266
H	-1.314946	3.171646	-2.364761
H	-0.626983	-2.159766	-4.568615

C2

C	0.039484	-0.117667	0.177959
C	0.155483	1.369415	0.018877
C	-0.971833	1.947958	-0.796339
C	-1.649802	0.698919	-1.424361
C	-0.850954	-0.543368	-1.006508
C	-0.087919	-0.944572	-2.282645
C	-0.544025	-2.373245	-2.575215
C	-2.038743	-2.358037	-2.170154
C	-1.966701	-1.631112	-0.801436
C	-1.784843	-2.467855	0.470230
C	-2.282727	-1.508563	1.559337
C	-3.164536	-0.736509	-0.439999
C	-1.517666	0.760916	-2.948333
C	-1.762298	2.693137	0.290834
C	-0.655685	3.058862	1.272593
C	-2.583862	3.886759	-0.162253
C	-2.785077	-3.713642	-2.305739
C	-4.173169	-3.652240	-1.646976
C	-1.962931	-4.889141	-1.754634
C	-3.035937	-3.981922	-3.802064
O	1.241633	-0.873694	0.159051
O	-0.434191	2.822024	-1.761961
O	-0.548581	-3.058559	0.701095
O	-2.026446	-1.511374	2.724317
O	-3.102697	-0.578156	0.984937
O	-3.004019	0.502481	-1.062000
O	-0.576995	-0.111651	-3.355948
O	-2.130435	1.479705	-3.683068
O	-0.603305	3.935494	2.071016
O	0.377265	2.124731	1.135570
O	-0.243719	-2.775984	-3.879867
H	-0.458214	-0.282219	1.145485
H	0.992878	-0.819511	-2.231380
H	-0.004671	-3.044978	-1.896743
H	-2.555929	-1.663846	-2.855918
H	-2.505673	-3.291243	0.461478
H	-4.154908	-1.110337	-0.700616
H	-2.399993	1.963312	0.810875
H	-3.371248	3.562372	-0.849043
H	-1.949718	4.624792	-0.659266
H	-3.048592	4.367482	0.701051
H	-4.151838	-3.520514	-0.560887
H	-4.709409	-4.586517	-1.842445
H	-4.770827	-2.838880	-2.076244
H	-1.622206	-4.749087	-0.725233
H	-1.075065	-5.052776	-2.374565
H	-2.563202	-5.804348	-1.793281
H	-3.638753	-3.182293	-4.248958
H	-3.589887	-4.919843	-3.917475
H	-2.098996	-4.065066	-4.354408
H	0.173591	-2.403717	0.673195
H	1.839832	-0.516660	0.834417
H	-1.157656	3.134156	-2.332653
H	-0.565664	-2.079477	-4.475952

C6

C	0.022516	-0.025071	0.070107
C	0.339381	1.427385	-0.285460
C	-0.967744	1.983076	-0.878269
C	-1.638812	0.747148	-1.536929
C	-0.873562	-0.502103	-1.082234
C	-0.155335	-0.958856	-2.330999
C	-0.515252	-2.399054	-2.587276
C	-2.030599	-2.376510	-2.195209
C	-2.000320	-1.579934	-0.859708
C	-1.863841	-2.353341	0.457087
C	-2.362085	-1.324089	1.475940
C	-3.203644	-0.659690	-0.582148
C	-1.430879	0.798812	-3.055853
C	-1.662464	2.533061	0.372047
C	-0.476387	2.905156	1.258592
C	-2.617377	3.695553	0.153946
C	-2.772852	-3.737706	-2.283792
C	-4.182904	-3.635876	-1.677492
C	-1.973360	-4.880655	-1.639665
C	-2.974409	-4.088519	-3.770403
O	1.147714	-0.868349	0.203039
O	-0.624453	2.990316	-1.793498
O	-0.649988	-2.959260	0.744427
O	-2.100697	-1.233580	2.635922
O	-3.193990	-0.446429	0.835998
O	-3.012319	0.555829	-1.248441
O	-0.503469	-0.143870	-3.408807
O	-1.966195	1.518562	-3.840116
O	-0.453387	3.666665	2.175167
O	0.635775	2.202143	0.864958
O	-0.211518	-2.843614	-3.874004
H	-0.535250	0.000612	1.016597
H	1.172527	1.497405	-0.992052
H	0.020005	-3.039900	-1.877331
H	-2.527034	-1.715035	-2.928291
H	-2.607694	-3.156495	0.473803
H	-4.187042	-1.034630	-0.866497
H	-2.193387	1.717403	0.882703

H	-3.478427	3.373559	-0.438485
H	-2.111830	4.520321	-0.353888
H	-2.971517	4.060496	1.120062
H	-4.199019	-3.442611	-0.600772
H	-4.715421	-4.578846	-1.838370
H	-4.762024	-2.847206	-2.173176
H	-1.671599	-4.685100	-0.607535
H	-1.062814	-5.076582	-2.215927
H	-2.572495	-5.797304	-1.653199
H	-3.555009	-3.311716	-4.282424
H	-3.532705	-5.027467	-3.850705
H	-2.020807	-4.208442	-4.285803
H	0.083457	-2.315418	0.712153
H	1.709327	-0.515610	0.912357
H	-1.418575	3.262422	-2.282831
H	-0.496875	-2.156078	-4.497866

C7

C	0.021682	-0.023036	0.045772
C	0.328656	1.434435	-0.301539
C	-0.982375	1.985862	-0.885582
C	-1.657484	0.747493	-1.539220
C	-0.881731	-0.502003	-1.100700
C	-0.112889	-0.940386	-2.363531
C	-0.678677	-2.277887	-2.667941
C	-2.112315	-2.330592	-2.217603
C	-2.011110	-1.566374	-0.866375
C	-1.848415	-2.360556	0.434666
C	-2.322128	-1.342544	1.479157
C	-3.203594	-0.648811	-0.549578
C	-1.466870	0.821220	-3.058213
C	-1.669379	2.535207	0.370155
C	-0.479606	2.908798	1.249485
C	-2.629234	3.694140	0.156525
C	-2.819862	-3.712667	-2.296889
C	-4.210244	-3.652218	-1.643429
C	-1.964744	-4.835181	-1.692006
C	-3.065561	-4.042587	-3.781893
O	1.161519	-0.854322	0.161082
O	-0.652084	2.996267	-1.802511
O	-0.633119	-2.972257	0.694891
O	-2.038515	-1.273016	2.635631
O	-3.159998	-0.448737	0.870593
O	-3.023836	0.565170	-1.215171
O	-0.467889	-0.000195	-3.427922
O	-2.074579	1.529154	-3.808359
O	-0.448755	3.670625	2.165638
O	0.630457	2.202306	0.851901
O	-0.282743	-2.962381	-3.765830
H	-0.521915	-0.010698	0.999456
H	1.157059	1.517945	-1.012600
H	0.975470	-0.901235	-2.270180
H	-2.702330	-1.667736	-2.887607
H	-2.595864	-3.159788	0.459048
H	-4.193086	-1.024865	-0.812293
H	-2.193787	1.718242	0.885334
H	-3.493235	3.366305	-0.428149
H	-2.130062	4.516644	-0.360769
H	-2.976646	4.061934	1.124065
H	-4.196078	-3.468794	-0.565215
H	-4.722620	-4.607621	-1.795422
H	-4.825844	-2.874693	-2.112008
H	-1.687296	-4.665239	-0.649469
H	-1.036766	-4.948177	-2.260891
H	-2.512905	-5.781685	-1.751606
H	-3.678442	-3.269151	-4.258979
H	-3.606859	-4.991779	-3.857458
H	-2.133127	-4.135225	-4.339633
H	0.105662	-2.344131	0.590264
H	1.702311	-0.524995	0.897106
H	-1.439352	3.218650	-2.327814
H	0.586846	-2.646671	-4.055535

C8

C	0.024877	0.015646	0.167449
C	0.315112	1.447243	-0.288358
C	-0.997522	1.922533	-0.937308
C	-1.652069	0.620660	-1.477957
C	-0.821243	-0.562526	-0.971049
C	-0.017494	-1.014070	-2.205853
C	-0.425642	-2.471295	-2.436979
C	-1.871516	-2.500434	-1.974215
C	-1.909756	-1.664790	-0.722738
C	-1.734409	-2.445616	0.598712
C	-2.220250	-1.420932	1.625484
C	-3.136211	-0.780968	-0.413717
C	-1.521824	0.563600	-3.001086
C	-1.711242	2.570469	0.254621
C	-0.544250	3.030259	1.122992
C	-2.679205	3.695162	-0.074320
C	-2.791233	-3.647416	-2.336451
C	-4.167340	-3.563656	-1.659671
C	-2.098076	-4.968172	-1.938076
C	-3.047001	-3.661123	-3.858465
O	1.169018	-0.783657	0.396677
O	-0.671460	2.842443	-1.946105
O	-0.497087	-3.019640	0.838600
O	-1.914739	-1.319678	2.774679
O	-3.071204	-0.551316	1.005794
O	-2.999962	0.426956	-1.094391
O	-0.500664	-0.255033	-3.332132
O	-2.181908	1.165356	-3.795374
O	-0.542644	3.864055	1.974778
O	0.581362	2.309830	0.805343
O	-0.140708	-2.908684	-3.734793
H	-0.561409	0.098673	1.092410
H	1.155406	1.491604	-0.988638
H	1.060266	-0.863422	-2.138489
H	0.171869	-3.101711	-1.758148
H	-2.459618	-3.267985	0.613170
H	-4.119130	-1.185042	-0.644369
H	-2.236753	1.791319	0.825058
H	-3.526810	3.309673	-0.648064
H	-2.178344	4.479831	-0.646286
H	-3.052205	4.137256	0.851631
H	-4.117948	-3.526665	-0.566588
H	-4.746634	-4.454204	-1.922440
H	-4.730869	-2.694123	-2.014567
H	-1.875199	-5.003542	-0.865678
H	-1.158533	-5.087096	-2.486498
H	-2.745103	-5.817479	-2.183578
H	-3.438552	-2.693905	-4.193354
H	-3.792114	-4.428715	-4.094846
H	-2.136156	-3.887012	-4.413182
H	0.191841	-2.330152	0.897290
H	1.695668	-0.366366	1.097542
H	-1.471259	3.046748	-2.459397
H	-0.507885	-2.245317	-4.342696

C8tBu

C	0.010198	-0.008676	0.122685
C	0.352037	1.435572	-0.246745
C	-0.932110	1.989406	-0.888950
C	-1.611099	0.743275	-1.521960
C	-0.845850	-0.499483	-1.050535
C	-0.049699	-0.947339	-2.291749
C	-0.526108	-2.372610	-2.566645
C	-2.023840	-2.331408	-2.188384
C	-1.981276	-1.561628	-0.845986
C	-1.874924	-2.372070	0.449883
C	-2.362527	-1.361683	1.490723
C	-3.179428	-0.643421	-0.553573
C	-1.427938	0.767092	-3.041814
C	-1.649862	2.587103	0.326424
C	-0.485510	2.961195	1.238819
C	-2.578197	3.757904	0.047754
C	-2.763367	-3.701653	-2.254973
C	-4.075313	-3.631865	-1.524354
C	-1.897049	-4.852839	-1.719784
C	-3.088862	-4.003677	-3.737514
O	1.125747	-0.857023	0.314667
O	-0.556336	2.959639	-1.831274
O	-0.670575	-2.999230	0.739323
O	-2.102373	-1.306734	2.653790

O	-3.165050	-0.446859	0.868374
O	-2.981485	0.575232	-1.206406
O	-0.475233	-0.121700	-3.396043
O	-2.014474	1.462148	-3.817784
O	-0.474401	3.744929	2.136696
O	0.624709	2.226723	0.898138
O	-0.217314	-2.812001	-3.857637
H	-0.581228	0.039413	1.047198
H	1.205840	1.491903	-0.929794
H	1.033596	-0.855283	-2.205826
H	-0.007973	-3.035599	-1.863837
H	-2.530327	-1.669450	-2.912674
H	-2.640354	-3.158368	0.419575
H	-4.162298	-1.019553	-0.836702
H	-2.208538	1.793206	0.842290
H	-3.426565	3.431781	-0.560526
H	-2.044351	4.558236	-0.470031
H	-2.954106	4.157062	0.991873
H	-4.416149	-4.477785	-0.936457
H	-4.839838	-2.944893	-1.877251
H	-1.519889	-4.678219	-0.708245
H	-1.036061	-5.014849	-2.376062
H	-2.485249	-5.776240	-1.715013
H	-3.734120	-3.226746	-4.162448
H	-3.618649	-4.958642	-3.810936
H	-2.172148	-4.064554	-4.328871
H	0.054274	-2.347538	0.787042
H	1.662868	-0.493580	1.037331
H	-1.334391	3.208733	-2.358051
H	-0.505485	-2.117841	-4.473448

C10

C	0.009858	-0.085400	-0.010739
C	0.409204	1.363478	-0.297317
C	-0.877544	2.029044	-0.838550
C	-1.706340	0.852404	-1.432241
C	-0.930385	-0.427769	-1.156510
C	-0.243333	-0.770230	-2.479795
C	-0.564988	-2.244013	-2.721852
C	-2.020945	-2.385727	-2.228435
C	-2.045649	-1.499666	-0.933943
C	-1.991069	-2.100594	0.425283
C	-3.115932	-1.680562	1.239608
C	-3.353663	-0.647633	-0.798650
C	-1.752799	0.930396	-2.965612
C	-1.459618	2.661034	0.429688
C	-0.223514	2.889269	1.291702
C	-2.286234	3.921342	0.232618
C	-2.619218	-3.814456	-2.180474
C	-4.019186	-3.768028	-1.551334
C	-1.735335	-4.803782	-1.410781
C	-2.779388	-4.334289	-3.619920
O	1.067917	-1.026369	0.021521
O	-0.493741	2.970772	-1.806105
O	-0.951821	-2.703306	1.003251
O	-3.363463	-1.914946	2.390100
O	-3.947205	-0.910002	0.439101
O	-2.980958	0.720988	-0.858390
O	-0.905597	0.014865	-3.492954
O	-2.424725	1.669291	-3.622190
O	-0.096641	3.630438	2.216539
O	0.791626	2.065919	0.872608
O	-0.313723	-2.638492	-4.039532
H	-0.542096	-0.099065	0.942385
H	1.223947	1.423061	-1.025850
H	0.825104	-0.551155	-2.526139
H	0.106812	-2.832640	-2.083310
H	-2.634825	-1.832891	-2.959893
H	-4.091017	-0.852858	-1.579214
H	-2.065729	1.907556	0.950834
H	-3.192600	3.694890	-0.336338
H	-1.706969	4.685313	-0.291609
H	-2.574184	4.323992	1.205621
H	-4.003169	-3.459922	-0.501815
H	-4.473936	-4.763171	-1.590201
H	-4.678154	-3.086199	-2.102625
H	-1.592853	-4.522099	-0.364582
H	-0.753242	-4.910229	-1.884762
H	-2.206783	-5.792094	-1.422323
H	-3.398075	-3.653929	-4.217638
H	-3.281098	-5.307571	-3.600856
H	-1.815673	-4.449196	-4.118785
H	-0.129242	-2.532381	0.501515
H	1.672365	-0.790425	0.743614
H	-1.291922	3.334211	-2.225652
H	-0.788802	-2.021585	-4.620529

C12

C	0.032215	-0.011756	0.117277
C	0.333382	1.440108	-0.256844
C	-0.965029	1.957627	-0.900786
C	-1.615029	0.689105	-1.520371
C	-0.808672	-0.531125	-1.051324
C	-0.025623	-0.973912	-2.299048
C	-0.498738	-2.405294	-2.567972
C	-1.993846	-2.357172	-2.178220
C	-1.924835	-1.617019	-0.817439
C	-1.782711	-2.431748	0.474689
C	-2.355780	-1.474061	1.527313
C	-3.086474	-0.708858	-0.493029
C	-1.456870	0.705326	-3.040382
C	-1.694123	2.549004	0.311942
C	-0.537116	2.956440	1.219630
C	-2.651131	3.694659	0.026052
C	-2.787156	-3.683154	-2.294119
C	-4.171683	-3.535749	-1.640498
C	-2.010101	-4.878207	-1.720525
C	-3.041194	-3.966619	-3.786698
O	1.171538	-0.824550	0.313983
O	-0.616025	2.926840	-1.852921
O	-0.554898	-3.002578	0.773364
O	-2.161777	-1.434652	2.700012
O	-3.194497	-0.576124	0.887625
O	-2.982741	0.488531	-1.146310
O	-0.476196	-0.151597	-3.398058
O	-2.071609	1.380061	-3.812218
O	-0.542963	3.745604	2.112367
O	0.588166	2.242722	0.883655
O	-0.191015	-2.837325	-3.861058
H	-0.567893	0.018995	1.039918
H	1.183924	1.513689	-0.942280
H	1.057973	-0.872056	-2.230760
H	0.025995	-3.070295	-1.870936
H	-2.492035	-1.658016	-2.872093
H	-2.496194	-3.260097	0.440475
H	-2.233281	1.747782	0.837290
H	-3.493348	3.343386	-0.576694
H	-2.137698	4.503213	-0.499480
H	-3.033850	4.092479	0.967955
H	-4.146942	-3.425449	-0.552552
H	-4.775160	-4.422868	-1.859405
H	-4.698072	-2.663501	-2.045600
H	-1.654842	-4.729419	-0.696921
H	-1.133517	-5.090567	-2.342200
H	-2.646356	-5.769493	-1.732529
H	-3.623243	-3.158706	-4.245732
H	-3.617709	-4.892084	-3.890657
H	-2.104943	-4.081476	-4.335444
H	0.151663	-2.329499	0.780957
H	1.702556	-0.439105	1.029840
H	-1.403011	3.158965	-2.374383
H	-0.511055	-2.156310	-4.475759

C14

C	0.080211	0.019883	0.057254
C	0.445192	1.458647	-0.321998
C	-0.832072	2.011563	-0.985143
C	-1.514289	0.765874	-1.612858
C	-0.786595	-0.482112	-1.101753
C	0.003678	-0.992362	-2.319037
C	-0.513508	-2.410443	-2.554330
C	-2.016405	-2.308340	-2.198841
C	-1.955877	-1.507380	-0.871240
C	-1.861123	-2.261428	0.459580

C	-2.323969	-1.192159	1.457520
C	-3.123907	-0.541331	-0.615382
C	-1.298696	0.753033	-3.130070
C	-1.502747	2.631000	0.202561
C	-0.480630	2.855798	1.224543
C	-2.871828	3.181300	0.299835
C	-2.817526	-3.636704	-2.281750
C	-4.223807	-3.474524	-1.681938
C	-2.070258	-4.807813	-1.625245
C	-3.025593	-3.988207	-3.767207
O	1.184991	-0.838143	0.266256
O	-0.471373	2.987204	-1.940055
O	-0.670713	-2.904425	0.769214
O	-2.078003	-1.109041	2.621326
O	-3.088625	-0.273020	0.793537
O	-2.896141	0.632063	-1.334378
O	-0.385178	-0.185312	-3.451359
O	-1.841330	1.459862	-3.928102
O	-0.566720	3.481007	2.246742
O	0.691999	2.229422	0.841794
O	-0.189597	-2.894644	-3.825205
H	-0.508166	0.097130	0.980708
H	1.314076	1.512830	-0.984713
H	1.088399	-0.931091	-2.225764
H	-0.026842	-3.067409	-1.823522
H	-2.481434	-1.631756	-2.938207
H	-2.633428	-3.036862	0.485980
H	-4.122027	-0.895054	-0.874676
H	-3.577429	2.392003	0.586478
H	-3.208585	3.583214	-0.661415
H	-2.899795	3.965389	1.060335
H	-4.233801	-3.278926	-0.605498
H	-4.795141	-4.394571	-1.842234
H	-4.767928	-2.663245	-2.180738
H	-1.763877	-4.615021	-0.593587
H	-1.167484	-5.048976	-2.196422
H	-2.708918	-5.697481	-1.630859
H	-3.573928	-3.192900	-4.286261
H	-3.618214	-4.906040	-3.845867
H	-2.074368	-4.147803	-4.276554
H	0.074225	-2.273915	0.777131
H	1.719332	-0.470156	0.988974
H	-1.234993	3.166877	-2.514716
H	-0.466132	-2.221552	-4.468997

C14Me

C	-0.003859	-0.027679	0.140040
C	0.310292	1.419068	-0.241090
C	-0.985331	1.946329	-0.874193
C	-1.657114	0.691534	-1.491782
C	-0.862843	-0.534755	-1.026996
C	-0.063671	-0.961251	-2.272396
C	-0.527010	-2.388971	-2.561627
C	-2.028333	-2.362193	-2.184696
C	-1.979310	-1.620895	-0.822677
C	-1.820840	-2.437480	0.465107
C	-2.302008	-1.440888	1.524251
C	-3.183329	-0.721437	-0.496132
C	-1.497792	0.712475	-3.014824
C	-1.704380	2.540529	0.353420
C	-0.522651	2.975381	1.218930
C	-2.673847	3.627432	0.068313
C	-2.777295	-3.716646	-2.318827
C	-4.177625	-3.642077	-1.688302
C	-1.972114	-4.887757	-1.734620
C	-2.998585	-4.002829	-3.816366
O	1.129955	-0.854506	0.320208
O	-0.635949	2.924040	-1.811862
O	-0.596442	-3.040661	0.715464
O	-2.014648	-1.389366	2.681072
O	-3.136225	-0.536703	0.928662
O	-3.017418	0.504355	-1.143056
O	-0.496864	-0.128802	-3.367308
O	-2.132942	1.356803	-3.792764
O	-0.500309	3.806754	2.071840
O	0.581808	2.231360	0.891032
O	-0.203498	-2.802828	-3.857043

H	-0.583076	0.013553	1.071980
H	1.156400	1.483189	-0.933170
H	1.018709	-0.861306	-2.185648
H	-0.006099	-3.058393	-1.866742
H	-2.530931	-1.673497	-2.887146
H	-2.558702	-3.246154	0.467564
H	-4.170338	-1.102794	-0.757789
H	-2.205888	1.720058	0.893630
H	-3.639895	3.379599	-0.352320
H	-2.488460	4.627068	0.437828
H	-4.175611	-3.500058	-0.603298
H	-4.714209	-4.575949	-1.884744
H	-4.763225	-2.830421	-2.136929
H	-1.654200	-4.734819	-0.699785
H	-1.071411	-5.063554	-2.332256
H	-2.575371	-5.800956	-1.774511
H	-3.589889	-3.207220	-4.285217
H	-3.552795	-4.940682	-3.931174
H	-2.051014	-4.094904	-4.348785
H	0.114418	-2.373109	0.759384
H	1.662004	-0.488914	1.045397
H	-1.450382	3.337787	-2.145384
H	-0.501353	-2.104090	-4.463186

C1OH

C	0.038410	-0.022949	0.133278
C	0.347070	1.435475	-0.247768
C	-0.952742	1.967376	-0.885443
C	-1.629742	0.716578	-1.503588
C	-0.852043	-0.525081	-1.056990
C	-0.065353	-0.959385	-2.308657
C	-0.529981	-2.390411	-2.576535
C	-2.028699	-2.355329	-2.190296
C	-1.970024	-1.598263	-0.835399
C	-1.821880	-2.409618	0.459785
C	-2.311496	-1.413035	1.511686
C	-3.171019	-0.690678	-0.515254
C	-1.478151	0.739341	-3.028260
C	-1.672486	2.565714	0.326760
C	-0.507248	2.964738	1.225505
C	-2.619832	3.719805	0.042655
C	-2.782716	-3.709167	-2.304356
C	-4.182568	-3.621169	-1.673761
C	-1.979705	-4.876632	-1.709658
C	-3.008532	-4.012802	-3.798174
O	1.115061	-0.857035	0.237978
O	-0.589750	2.931395	-1.838039
O	-0.600143	-3.014581	0.719399
O	-2.029908	-1.365826	2.669702
O	-3.137916	-0.509011	0.908831
O	-2.990855	0.534910	-1.158535
O	-0.522028	-0.137569	-3.401662
O	-2.091413	1.425779	-3.789865
O	-0.499863	3.763057	2.110154
O	0.604773	2.234911	0.889067
O	-0.214190	-2.820559	-3.867571
H	-0.544689	-0.013541	1.067468
H	1.198042	1.497589	-0.932616
H	1.017629	-0.851074	-2.244885
H	-0.004615	-3.050296	-1.875769
H	-2.533784	-1.673150	-2.897115
H	-2.561022	-3.216327	0.450377
H	-4.157069	-1.065893	-0.788820
H	-2.214635	1.768077	0.854309
H	-3.464607	3.378567	-0.562479
H	-2.099150	4.526754	-0.478500
H	-2.999789	4.118256	0.985379
H	-4.182408	-3.462514	-0.591061
H	-4.721211	-4.556633	-1.855837
H	-4.766404	-2.815519	-2.135301
H	-1.662073	-4.718983	-0.675410
H	-1.079175	-5.059389	-2.305348
H	-2.584887	-5.788706	-1.742736
H	-3.593552	-3.218464	-4.276941
H	-3.571588	-4.946797	-3.899270
H	-2.063085	-4.121875	-4.331018
H	0.070023	-2.356407	0.975161

H	-1.379784	3.199604	-2.336886
H	-0.530985	-2.140292	-4.484868

C3OH

C	-0.011151	-0.008320	0.138185
C	0.341728	1.428444	-0.257617
C	-0.991854	1.963988	-0.904399
C	-1.659157	0.692773	-1.502859
C	-0.859712	-0.526682	-1.026737
C	-0.052851	-0.959012	-2.264930
C	-0.526097	-2.384045	-2.557621
C	-2.027882	-2.350414	-2.181201
C	-1.975367	-1.612455	-0.817584
C	-1.813810	-2.431950	0.467904
C	-2.276832	-1.430349	1.529799
C	-3.175009	-0.710723	-0.482185
C	-1.509750	0.686807	-3.029882
C	-1.676689	2.585789	0.323623
C	-0.497223	2.976058	1.207664
C	-2.602570	3.755743	0.024631
C	-2.783191	-3.701268	-2.317224
C	-4.182015	-3.621156	-1.684229
C	-1.983458	-4.877657	-1.736398
C	-3.007364	-3.982363	-3.815272
O	1.115428	-0.833589	0.351395
O	-0.595938	2.884699	-1.817050
O	-0.591949	-3.044061	0.709050
O	-1.968809	-1.374420	2.681370
O	-3.112896	-0.523505	0.942481
O	-3.010641	0.513934	-1.131295
O	-0.467544	-0.122027	-3.360277
O	-2.177847	1.266117	-3.825798
O	-0.472245	3.764575	2.098179
O	0.618852	2.247443	0.851073
O	-0.204647	-2.794947	-3.853950
H	-0.600398	0.066334	1.061189
H	1.185650	1.461655	-0.953590
H	1.029955	-0.871318	-2.167702
H	-0.010173	-3.059354	-1.864767
H	-2.528571	-1.659117	-2.882291
H	-2.558204	-3.234581	0.474139
H	-4.165471	-1.089469	-0.733829
H	-2.231665	1.802305	0.858705
H	-3.473052	3.409250	-0.536898
H	-2.079839	4.515764	-0.560372
H	-2.934951	4.203941	0.963192
H	-4.177013	-3.484473	-0.598449
H	-4.724361	-4.550981	-1.883879
H	-4.763677	-2.804096	-2.128076
H	-1.666209	-4.729866	-0.700579
H	-1.083003	-5.055659	-2.333725
H	-2.590865	-5.787907	-1.779907
H	-3.596572	-3.183476	-4.281035
H	-3.564711	-4.918098	-3.931933
H	-2.060750	-4.075849	-4.349165
H	0.117502	-2.377036	0.768774
H	1.621358	-0.475940	1.099230
H	-0.490911	-2.088399	-4.456891

C7OH

C	0.017074	-0.018901	0.104881
C	0.335150	1.430122	-0.267620
C	-0.965252	1.969457	-0.889853
C	-1.635068	0.715711	-1.516613
C	-0.852776	-0.518561	-1.054922
C	-0.069021	-0.954354	-2.313222
C	-0.501194	-2.411442	-2.533251
C	-2.033872	-2.361815	-2.179810
C	-1.972422	-1.593550	-0.834221
C	-1.826811	-2.395487	0.465117
C	-2.314923	-1.384958	1.507451
C	-3.178216	-0.685851	-0.529168
C	-1.464762	0.741024	-3.038850
C	-1.672880	2.554684	0.338013
C	-0.500131	2.940607	1.234720
C	-2.619191	3.714719	0.075450
C	-2.780623	-3.713350	-2.304774
C	-4.183604	-3.623091	-1.679340
C	-1.981502	-4.879731	-1.704428
C	-2.998201	-4.004041	-3.800623
O	1.146753	-0.853554	0.277104
O	-0.616142	2.948572	-1.832157
O	-0.608566	-3.002835	0.733359
O	-2.038028	-1.321497	2.665846
O	-3.145458	-0.490528	0.893534
O	-3.001342	0.530798	-1.186381
O	-0.523685	-0.148017	-3.405528
O	-2.056945	1.449124	-3.799809
O	-0.485079	3.721287	2.135013
O	0.614630	2.220790	0.875520
O	-0.293749	-2.893172	-3.780274
H	-0.557070	0.022282	1.040334
H	1.177734	1.499004	-0.963251
H	1.013057	-0.839833	-2.233410
H	-0.026930	-3.043268	-1.761103
H	-2.493232	-1.682816	-2.914910
H	-2.569035	-3.200130	0.471003
H	-4.163791	-1.066485	-0.797487
H	-2.213846	1.753254	0.861022
H	-3.473747	3.378807	-0.518547
H	-2.102956	4.519490	-0.452791
H	-2.983282	4.109930	1.025873
H	-4.183276	-3.471461	-0.595909
H	-4.721995	-4.556797	-1.869492
H	-4.764275	-2.814095	-2.138234
H	-1.682827	-4.723294	-0.664425
H	-1.071068	-5.050376	-2.288774
H	-2.580813	-5.794715	-1.753228
H	-3.598180	-3.217949	-4.272190
H	-3.535028	-4.951698	-3.915604
H	-2.050844	-4.085143	-4.339171
H	0.108399	-2.341013	0.771382
H	1.696534	-0.478612	0.984388
H	-1.392042	3.154659	-2.380743

C10OH

C	0.054603	-0.057690	0.068483
C	0.337848	1.421493	-0.231196
C	-0.946608	1.979639	-0.871323
C	-1.611795	0.748092	-1.541247
C	-0.850847	-0.499538	-1.091362
C	-0.077455	-0.949681	-2.344254
C	-0.535346	-2.383709	-2.592748
C	-2.031772	-2.358095	-2.199014
C	-1.983069	-1.561675	-0.870129
C	-1.829922	-2.307125	0.463804
C	-2.430162	-1.326221	1.487031
C	-3.193131	-0.646501	-0.597954
C	-1.437145	0.793532	-3.062535
C	-1.683472	2.541923	0.348925
C	-0.533855	2.899512	1.285230
C	-2.618854	3.711114	0.089065
C	-2.769859	-3.722613	-2.273618
C	-4.154241	-3.646557	-1.608505
C	-1.933581	-4.867764	-1.680508
C	-3.025268	-4.057592	-3.755395
O	1.197851	-0.872685	0.107948
O	-0.572809	2.975114	-1.788830
O	-0.603607	-2.803342	0.777946
O	-2.251060	-1.298870	2.664358
O	-3.225355	-0.444579	0.825185
O	-2.989591	0.567022	-1.246573
O	-0.537030	-0.137043	-3.444592
O	-2.003764	1.524811	-3.820045
O	-0.543239	3.662332	2.202249
O	0.579256	2.177594	0.945751
O	-0.233564	-2.834015	-3.881827
H	-0.480830	-0.096232	1.028014
H	1.198363	1.526080	-0.899470
H	1.005072	-0.843434	-2.278213
H	0.006390	-3.025026	-1.887333
H	-2.544348	-1.696950	-2.920102
H	-2.486482	-3.196692	0.474507
H	-4.168806	-1.026025	-0.902217

H	-2.239394	1.731676	0.841022
H	-3.459901	3.393964	-0.534074
H	-2.086248	4.526494	-0.406002
H	-3.004876	4.086206	1.038990
H	-4.132252	-3.443234	-0.532361
H	-4.674288	-4.601052	-1.738555
H	-4.770444	-2.873600	-2.082919
H	-1.590048	-4.685478	-0.658115
H	-1.045838	-5.047341	-2.295447
H	-2.523982	-5.789892	-1.676156
H	-3.640012	-3.284646	-4.231356
H	-3.567379	-5.006533	-3.829322
H	-2.089312	-4.149905	-4.308051
H	1.616286	-0.784634	0.976489
H	-1.347328	3.222572	-2.321465
H	-0.531840	-2.148428	-4.502538

1J

C	0.082834	-0.035390	0.056742
C	0.354564	1.437053	-0.272906
C	-0.950627	1.975524	-0.895369
C	-1.616227	0.735171	-1.538854
C	-0.832419	-0.505189	-1.086984
C	-0.079386	-0.946954	-2.355183
C	-0.540569	-2.377805	-2.619013
C	-2.032654	-2.348496	-2.211738
C	-1.961282	-1.575463	-0.866658
C	-1.817364	-2.353556	0.434877
C	-2.334804	-1.358175	1.462655
C	-3.171892	-0.665382	-0.567383
C	-1.469658	0.773763	-3.061439
C	-1.669322	2.541786	0.332846
C	-0.503494	2.930719	1.235946
C	-2.628657	3.692781	0.079163
C	-2.774835	-3.709976	-2.296659
C	-4.168935	-3.626940	-1.652234
C	-1.948562	-4.855466	-1.689406
C	-3.014158	-4.045591	-3.781165
O	-0.602680	2.967239	-1.828339
O	-0.518013	-2.787411	0.713788
O	-2.047603	-1.343144	2.625399
O	-3.164860	-0.476874	0.866324
O	-2.987222	0.549510	-1.211126
O	-0.541556	-0.129706	-3.449397
O	-2.066531	1.479372	-3.819681
O	-0.506847	3.709505	2.140827
O	0.610400	2.224679	0.881543
O	-0.243705	-2.808095	-3.916316
H	-0.410068	-0.072686	1.032616
H	1.189540	1.572316	-0.966757
H	1.006287	-0.838901	-2.305855
H	-0.000935	-3.037861	-1.929148
H	-2.550077	-1.676628	-2.919099
H	-2.518872	-3.198545	0.447311
H	-4.155385	-1.046283	-0.842168
H	-2.200034	1.729527	0.847720
H	-3.477953	3.355790	-0.522106
H	-2.119728	4.510972	-0.436076
H	-2.999648	4.073960	1.032683
H	-4.159681	-3.441554	-0.573869
H	-4.697710	-4.572970	-1.807367
H	-4.767355	-2.839598	-2.126239
H	-1.589509	-4.655485	-0.675472
H	-1.071338	-5.060198	-2.312067
H	-2.551499	-5.769189	-1.661191
H	-3.619295	-3.270076	-4.265526
H	-3.561735	-4.991196	-3.858617
H	-2.073713	-4.145162	-4.324453
H	-0.423624	-2.789695	1.683232
H	-1.393234	3.217746	-2.334854
H	-0.552573	-2.115933	-4.524495
H	0.990003	-0.636744	0.137102

C1

C	0.091660	-0.054100	-0.031295
C	0.307697	1.419622	-0.136003
C	-0.941584	1.970498	-0.866122
C	-1.582058	0.731928	-1.560548
C	-0.794787	-0.519938	-1.140396
C	-0.079506	-0.968609	-2.439671
C	-0.565877	-2.392611	-2.696997
C	-2.040288	-2.360330	-2.230711
C	-1.917575	-1.594274	-0.888788
C	-1.718986	-2.351756	0.416081
C	-2.114475	-1.298750	1.441020
C	-3.102555	-0.671782	-0.542599
C	-1.441105	0.807867	-3.080607
C	-1.761496	2.555581	0.288344
C	-0.710672	2.839813	1.352149
C	-2.610661	3.770980	-0.046526
C	-2.792351	-3.717634	-2.287740
C	-4.163808	-3.619388	-1.599537
C	-1.959030	-4.863641	-1.693103
C	-3.077634	-4.060732	-3.761876
O	-0.501076	2.937089	-1.786206
O	-0.429032	-2.849128	0.622817
O	-1.731461	-1.242458	2.572572
O	-2.997073	-0.439510	0.879364
O	-2.952655	0.516366	-1.244013
O	-0.565554	-0.130897	-3.506260
O	-2.008063	1.563641	-3.813593
O	-0.799633	3.576230	2.287914
O	0.402431	2.087206	1.118032
O	-0.323989	-2.811117	-4.009713
H	1.211204	1.675250	-0.706884
H	1.007611	-0.872931	-2.418977
H	-0.002317	-3.062448	-2.036639
H	-2.581197	-1.682422	-2.914522
H	-2.462395	-3.157019	0.499100
H	-4.103671	-1.052243	-0.744446
H	-2.396152	1.768486	0.711689
H	-3.398926	3.502768	-0.756637
H	-1.996052	4.567705	-0.472387
H	-3.073968	4.151652	0.865864
H	-4.113452	-3.438857	-0.521502
H	-4.709154	-4.558362	-1.739434
H	-4.767787	-2.822614	-2.050320
H	-1.581723	-4.659283	-0.686805
H	-1.094478	-5.075791	-2.330864
H	-2.565420	-5.774495	-1.649554
H	-3.687865	-3.281837	-4.234429
H	-3.637704	-5.000554	-3.816646
H	-2.154514	-4.174322	-4.331412
H	-0.277224	-2.834484	1.585168
H	-1.253903	3.217405	-2.333462
H	-0.658464	-2.114688	-4.599019
H	0.734616	-0.709300	0.539083

C2

C	0.151454	-0.127540	0.092932
C	0.098016	1.368438	0.090151
C	-0.980213	1.954941	-0.778570
C	-1.626744	0.711351	-1.443495
C	-0.805712	-0.535552	-1.056729
C	-0.108403	-0.933874	-2.369949
C	-0.565043	-2.362185	-2.654267
C	-2.041770	-2.354809	-2.191735
C	-1.921297	-1.625149	-0.826488
C	-1.731971	-2.447097	0.440709
C	-2.189733	-1.478468	1.523724
C	-3.117913	-0.732648	-0.444495
C	-1.526471	0.805188	-2.966557
C	-1.828499	2.713489	0.251838
C	-0.807111	3.004894	1.345001
C	-2.553500	3.954201	-0.239389
C	-2.784082	-3.715059	-2.295879
C	-4.156747	-3.656825	-1.604327
C	-1.938543	-4.878588	-1.754015
C	-3.071982	-4.001715	-3.782117
O	-0.382901	2.810288	-1.732384
O	-0.431103	-2.917224	0.643521
O	-1.866797	-1.517493	2.676504
O	-3.015625	-0.554581	0.987725
O	-2.983705	0.486687	-1.085542

O	-0.634320	-0.098282	-3.421324
O	-2.132260	1.556174	-3.674446
O	-0.820172	3.850551	2.181747
O	0.207628	2.061995	1.265539
O	-0.313987	-2.755164	-3.973054
H	-0.153305	-0.492180	1.077530
H	0.975444	-0.806120	-2.369167
H	0.004663	-3.036034	-2.003031
H	-2.586827	-1.662491	-2.856839
H	-2.449712	-3.278610	0.454316
H	-4.113911	-1.119673	-0.660138
H	-2.540604	2.005154	0.696371
H	-3.294364	3.684710	-0.998573
H	-1.846650	4.671427	-0.663597
H	-3.066531	4.438326	0.594151
H	-4.112394	-3.518234	-0.519873
H	-4.692868	-4.594707	-1.782187
H	-4.767798	-2.849053	-2.025007
H	-1.555251	-4.715259	-0.742553
H	-1.076489	-5.055509	-2.405736
H	-2.537449	-5.795420	-1.745761
H	-3.692286	-3.210944	-4.220542
H	-3.622452	-4.944477	-3.872503
H	-2.149952	-4.083006	-4.358818
H	-0.305083	-2.996618	1.605919
H	-1.083043	3.155867	-2.312802
H	-0.651756	-2.050030	-4.550139
H	1.154379	-0.536283	-0.073331

C6

C	0.103514	-0.044310	-0.006252
C	0.365084	1.435791	-0.305024
C	-0.952515	1.990562	-0.886539
C	-1.615305	0.771565	-1.570508
C	-0.849647	-0.486973	-1.133425
C	-0.175388	-0.943492	-2.405825
C	-0.533916	-2.383843	-2.656895
C	-2.037385	-2.369639	-2.226239
C	-1.983127	-1.556322	-0.899419
C	-1.856057	-2.303286	0.422300
C	-2.379437	-1.281578	1.418689
C	-3.195541	-0.634235	-0.636513
C	-1.428381	0.843757	-3.090385
C	-1.654889	2.505432	0.373081
C	-0.473686	2.875682	1.265405
C	-2.628493	3.656358	0.179642
C	-2.766138	-3.739377	-2.275859
C	-4.169859	-3.641775	-1.652984
C	-1.938392	-4.856790	-1.620575
C	-2.985820	-4.127577	-3.750564
O	-0.620491	3.022768	-1.780276
O	-0.565065	-2.744538	0.721857
O	-2.097596	-1.227130	2.581276
O	-3.216642	-0.426665	0.792547
O	-2.995794	0.576379	-1.289920
O	-0.541896	-0.128012	-3.474986
O	-1.953449	1.588109	-3.858486
O	-0.467692	3.624481	2.195338
O	0.639065	2.194520	0.865104
O	-0.262819	-2.823905	-3.953336
H	-0.360279	-0.111534	0.982334
H	1.187081	1.587277	-1.010285
H	0.024154	-3.024969	-1.964640
H	-2.556412	-1.720625	-2.954274
H	-2.565995	-3.140985	0.446760
H	-4.175190	-1.012600	-0.928711
H	-2.169288	1.672963	0.871142
H	-3.487963	3.334256	-0.415233
H	-2.136893	4.497525	-0.315096
H	-2.982188	3.999760	1.153853
H	-4.178516	-3.417187	-0.582284
H	-4.691073	-4.595974	-1.781637
H	-4.765458	-2.875613	-2.163980
H	-1.592332	-4.621339	-0.610069
H	-1.052169	-5.074270	-2.226038
H	-2.533880	-5.774455	-1.570513
H	-3.586625	-3.371254	-4.269592
H	-3.530881	-5.076449	-3.800358
H	-2.039267	-4.242798	-4.279456
H	-0.473464	-2.717317	1.691146
H	-1.418899	3.300938	-2.258433
H	-0.549107	-2.126135	-4.565405
H	1.011914	-0.647173	0.020961

C7

C	0.077975	-0.042444	-0.009610
C	0.352179	1.433608	-0.316965
C	-0.964179	1.989514	-0.896342
C	-1.639553	0.767021	-1.566987
C	-0.863862	-0.490209	-1.142248
C	-0.131712	-0.925589	-2.428284
C	-0.703130	-2.258823	-2.738099
C	-2.122610	-2.322573	-2.248234
C	-1.996946	-1.547278	-0.903488
C	-1.837657	-2.320405	0.399764
C	-2.349367	-1.321781	1.428015
C	-3.199466	-0.629767	-0.596122
C	-1.471533	0.858403	-3.085938
C	-1.656716	2.519446	0.363198
C	-0.469784	2.889016	1.247251
C	-2.624430	3.674036	0.163256
C	-2.809356	-3.716139	-2.292666
C	-4.187342	-3.671312	-1.610637
C	-1.912057	-4.810179	-1.694886
C	-3.085024	-4.076795	-3.765209
O	-0.635147	3.014110	-1.800287
O	-0.537460	-2.752085	0.672436
O	-2.053302	-1.301570	2.588623
O	-3.187337	-0.444143	0.836927
O	-3.010978	0.583798	-1.241874
O	-0.496246	0.018386	-3.483297
O	-2.077811	1.581543	-3.822339
O	-0.452164	3.644853	2.171373
O	0.637409	2.196063	0.847771
O	-0.342478	-2.920044	-3.863413
H	-0.397659	-0.092241	0.974094
H	1.171553	1.579741	-1.027116
H	0.960396	-0.890679	-2.360876
H	-2.738370	-1.673459	-2.907947
H	-2.538259	-3.164946	0.424121
H	-4.185364	-1.007774	-0.869111
H	-2.173678	1.693815	0.870094
H	-3.487337	3.349968	-0.425418
H	-2.130042	4.506154	-0.343508
H	-2.972443	4.029171	1.135358
H	-4.160450	-3.458555	-0.538196
H	-4.681699	-4.640819	-1.729571
H	-4.828221	-2.919606	-2.087169
H	-1.576604	-4.600123	-0.676102
H	-1.015817	-4.934593	-2.310336
H	-2.450456	-5.764016	-1.687397
H	-3.723340	-3.322633	-4.239525
H	-3.611062	-5.036521	-3.810857
H	-2.165371	-4.162112	-4.344446
H	-0.440690	-2.747705	1.641634
H	-1.428557	3.258521	-2.305491
H	0.490124	-2.559888	-4.205399
H	0.980163	-0.653405	0.046191

C8

C	0.104737	0.004321	0.107033
C	0.336264	1.466170	-0.294763
C	-0.983967	1.938275	-0.940617
C	-1.625306	0.649721	-1.510016
C	-0.794091	-0.541105	-1.013864
C	-0.029024	-0.999125	-2.271224
C	-0.428452	-2.459724	-2.486666
C	-1.862832	-2.495873	-1.992269
C	-1.885877	-1.637431	-0.752139
C	-1.721142	-2.381253	0.578362
C	-2.263263	-1.372875	1.578352
C	-3.128517	-0.754593	-0.471326
C	-1.512474	0.611815	-3.034140
C	-1.712540	2.552976	0.258219

C	-0.555366	3.015536	1.136748
C	-2.699445	3.665680	-0.053525
C	-2.781778	-3.652870	-2.326285
C	-4.147669	-3.576763	-1.627184
C	-2.071268	-4.964993	-1.929960
C	-3.064852	-3.684369	-3.843452
O	-0.664150	2.883282	-1.930366
O	-0.407633	-2.765112	0.854517
O	-1.967778	-1.312992	2.737820
O	-3.111243	-0.527009	0.958664
O	-2.981682	0.447681	-1.141855
O	-0.536359	-0.251500	-3.392267
O	-2.159501	1.244412	-3.815110
O	-0.575552	3.840316	1.999743
O	0.573673	2.316803	0.817982
O	-0.170536	-2.903573	-3.788621
H	-0.391410	-0.000389	1.082386
H	1.166282	1.588358	-0.996980
H	1.050581	-0.839545	-2.233968
H	0.191158	-3.083327	-1.821155
H	-2.394537	-3.251838	0.595182
H	-4.103643	-1.165926	-0.720271
H	-2.222950	1.755978	0.816016
H	-3.540920	3.277188	-0.634439
H	-2.210880	4.467611	-0.612303
H	-3.078705	4.087845	0.879227
H	-4.082562	-3.510456	-0.536243
H	-4.714742	-4.483793	-1.858359
H	-4.733647	-2.727728	-1.994614
H	-1.836650	-4.993316	-0.859481
H	-1.137485	-5.078781	-2.489166
H	-2.712969	-5.822015	-2.161946
H	-3.465861	-2.722176	-4.181220
H	-3.811709	-4.456819	-4.057496
H	-2.163393	-3.912420	-4.412240
H	-0.298963	-2.726233	1.821528
H	-1.465808	3.094022	-2.437584
H	-0.545966	-2.239489	-4.390933
H	1.028276	-0.566853	0.216934

C8tBu

C	0.082849	-0.030728	0.056558
C	0.374394	1.441142	-0.258077
C	-0.917149	1.999374	-0.892417
C	-1.590740	0.771892	-1.551768
C	-0.826932	-0.481459	-1.098213
C	-0.072524	-0.927535	-2.366190
C	-0.542021	-2.355091	-2.630078
C	-2.030410	-2.323845	-2.218307
C	-1.968582	-1.535353	-0.885336
C	-1.872607	-2.320603	0.415584
C	-2.391440	-1.320710	1.435398
C	-3.175974	-0.614150	-0.607491
C	-1.431815	0.817087	-3.072783
C	-1.643509	2.566745	0.330720
C	-0.484257	2.934809	1.250720
C	-2.584956	3.731520	0.072888
C	-2.751659	-3.704093	-2.245249
C	-4.058198	-3.635963	-1.504617
C	-1.859702	-4.826690	-1.690325
C	-3.088745	-4.051357	-3.715508
O	-0.545756	2.992355	-1.815083
O	-0.591515	-2.785451	0.728473
O	-2.122992	-1.310077	2.602933
O	-3.194626	-0.424672	0.825078
O	-2.968310	0.600815	-1.244535
O	-0.525139	-0.107487	-3.461434
O	-2.007598	1.540496	-3.830532
O	-0.488978	3.707498	2.160832
O	0.625123	2.217301	0.905199
O	-0.261004	-2.794540	-3.928165
H	-0.422189	-0.071946	1.026266
H	1.219019	1.572473	-0.940945
H	1.013821	-0.826701	-2.312495
H	-0.001136	-3.014588	-1.941135
H	-2.558475	-1.677214	-2.940384
H	-2.605872	-3.142269	0.390062
H	-4.155251	-0.991615	-0.900405
H	-2.191047	1.758073	0.833507
H	-3.430897	3.409180	-0.541009
H	-2.059407	4.546499	-0.430637
H	-2.962954	4.111117	1.024283
H	-4.398816	-4.489710	-0.927720
H	-4.825737	-2.950830	-1.854541
H	-1.451013	-4.608112	-0.698855
H	-1.017967	-5.009521	-2.365622
H	-2.437743	-5.754235	-1.626353
H	-3.751066	-3.295979	-4.152513
H	-3.604583	-5.015905	-3.757008
H	-2.178344	-4.112638	-4.316124
H	-0.520531	-2.780351	1.699980
H	-1.327069	3.254948	-2.329758
H	-0.556639	-2.096462	-4.536152
H	0.982176	-0.643378	0.142215

C10

C	0.098657	-0.034970	-0.004444
C	0.387608	1.444821	-0.282077
C	-0.909606	2.011825	-0.900912
C	-1.590787	0.795635	-1.574379
C	-0.811201	-0.456325	-1.164524
C	-0.090901	-0.894937	-2.448934
C	-0.531887	-2.339183	-2.685119
C	-2.009318	-2.347101	-2.235839
C	-1.943476	-1.526303	-0.917067
C	-1.805762	-2.173672	0.404143
C	-2.566482	-1.448687	1.394329
C	-3.184946	-0.628947	-0.660139
C	-1.468137	0.858791	-3.099750
C	-1.632694	2.551447	0.336965
C	-0.473459	2.898148	1.264579
C	-2.575918	3.720578	0.107171
C	-2.719132	-3.728569	-2.249971
C	-4.043432	-3.673955	-1.475522
C	-1.831880	-4.840203	-1.674942
C	-3.054783	-4.088498	-3.707624
O	-0.545159	3.019578	-1.808834
O	-0.782966	-2.941264	0.783148
O	-2.504236	-1.597897	2.592684
O	-3.339305	-0.516239	0.761869
O	-2.957079	0.618834	-1.233943
O	-0.601501	-0.089267	-3.527620
O	-2.043610	1.607407	-3.832263
O	-0.476970	3.646337	2.194508
O	0.637719	2.190818	0.899519
O	-0.269956	-2.782363	-3.984975
H	-0.423923	-0.100397	0.956989
H	1.230302	1.594536	-0.963235
H	0.994552	-0.769159	-2.432543
H	0.046879	-2.979282	-2.007394
H	-2.562414	-1.707057	-2.945677
H	-4.123894	-1.013754	-1.058479
H	-2.181043	1.729957	0.819086
H	-3.422867	3.410090	-0.511262
H	-2.052247	4.546587	-0.380079
H	-2.952266	4.078669	1.067474
H	-3.903040	-3.437498	-0.414611
H	-4.538626	-4.648945	-1.529160
H	-4.730245	-2.938727	-1.911181
H	-1.514888	-4.638067	-0.648513
H	-0.939104	-4.986770	-2.291889
H	-2.388123	-5.783486	-1.675355
H	-3.710401	-3.333837	-4.158040
H	-3.582287	-5.047921	-3.737717
H	-2.150865	-4.172759	-4.313769
H	-0.836586	-3.016517	1.758349
H	-1.329210	3.286666	-2.317197
H	-0.624575	-2.112442	-4.593045
H	1.000146	-0.648085	0.075465

C12

C	0.102742	-0.018441	0.062968
C	0.355483	1.454976	-0.274152
C	-0.952320	1.969211	-0.912844

C	-1.595645	0.712528	-1.547878
C	-0.785378	-0.511993	-1.088063
C	-0.039015	-0.956772	-2.358342
C	-0.506050	-2.390424	-2.616489
C	-1.992622	-2.347903	-2.198399
C	-1.904119	-1.592734	-0.845232
C	-1.766792	-2.383185	0.451197
C	-2.383066	-1.445806	1.482524
C	-3.076461	-0.686165	-0.533402
C	-1.463383	0.739388	-3.068649
C	-1.688615	2.537090	0.305175
C	-0.535448	2.950474	1.213975
C	-2.663669	3.670581	0.033686
C	-2.777589	-3.680601	-2.287124
C	-4.151250	-3.541350	-1.608539
C	-1.975226	-4.862667	-1.720155
C	-3.058793	-3.981501	-3.771381
O	-0.612645	2.954898	-1.853740
O	-0.456743	-2.757111	0.764889
O	-2.186926	-1.457500	2.660521
O	-3.214794	-0.562740	0.855071
O	-2.968930	0.507622	-1.173316
O	-0.512214	-0.142167	-3.451840
O	-2.077912	1.427678	-3.828480
O	-0.557446	3.737233	2.111413
O	0.590150	2.255685	0.875576
O	-0.223710	-2.824404	-3.915535
H	-0.416488	-0.060511	1.027130
H	1.193997	1.595829	-0.962534
H	1.046792	-0.847185	-2.319014
H	0.037405	-3.052587	-1.930986
H	-2.506265	-1.656928	-2.888458
H	-2.425271	-3.260166	0.419763
H	-2.211563	1.722741	0.825120
H	-3.503474	3.315352	-0.570353
H	-2.163250	4.492098	-0.484610
H	-3.047480	4.054472	0.980976
H	-4.109179	-3.419262	-0.522312
H	-4.749968	-4.436274	-1.808032
H	-4.693134	-2.678454	-2.012881
H	-1.582387	-4.692399	-0.713013
H	-1.120849	-5.083508	-2.368981
H	-2.606440	-5.756980	-1.688974
H	-3.662382	-3.185963	-4.223794
H	-3.623591	-4.916240	-3.855539
H	-2.132699	-4.085772	-4.338910
H	-0.410737	-2.864225	1.730912
H	-1.406175	3.199287	-2.358762
H	-0.543493	-2.136462	-4.522844
H	1.016203	-0.605384	0.170395

C14

C	0.171858	-0.011648	-0.015942
C	0.462545	1.467328	-0.308745
C	-0.818384	2.019434	-0.978761
C	-1.486074	0.791219	-1.640833
C	-0.755836	-0.467575	-1.153283
C	-0.016667	-0.975742	-2.403400
C	-0.530136	-2.395230	-2.627029
C	-2.021133	-2.300879	-2.226534
C	-1.929737	-1.485143	-0.907868
C	-1.836272	-2.212429	0.426722
C	-2.316695	-1.148765	1.405868
C	-3.105240	-0.517309	-0.659299
C	-1.297146	0.806527	-3.160232
C	-1.520993	2.603072	0.206890
C	-0.527884	2.796921	1.263429
C	-2.886984	3.167047	0.278501
C	-2.811061	-3.637305	-2.268851
C	-4.208229	-3.478204	-1.646108
C	-2.034342	-4.786068	-1.605375
C	-3.045745	-4.022927	-3.741790
O	-0.453279	3.018515	-1.910133
O	-0.563328	-2.697708	0.739843
O	-2.049550	-1.105556	2.572197
O	-3.088786	-0.249237	0.759813
O	-2.874812	0.648949	-1.371131
O	-0.438863	-0.170510	-3.522905
O	-1.825445	1.548524	-3.935673
O	-0.654683	3.388611	2.303296
O	0.653459	2.200905	0.892631
O	-0.243958	-2.875031	-3.909475
H	-0.312390	-0.052427	0.962744
H	1.331747	1.626909	-0.952208
H	1.072098	-0.908351	-2.350245
H	-0.016117	-3.052693	-1.915409
H	-2.511194	-1.634413	-2.958237
H	-2.578888	-3.020881	0.466507
H	-4.101942	-0.875348	-0.917714
H	-3.609626	2.387770	0.549107
H	-3.200152	3.578400	-0.686732
H	-2.920506	3.947759	1.042538
H	-4.203795	-3.254097	-0.575263
H	-4.770888	-4.408732	-1.773426
H	-4.770742	-2.686783	-2.156043
H	-1.682195	-4.558248	-0.594898
H	-1.156769	-5.046143	-2.206535
H	-2.669631	-5.676360	-1.550379
H	-3.614480	-3.244828	-4.264717
H	-3.629112	-4.948916	-3.788605
H	-2.103866	-4.180418	-4.268410
H	-0.479791	-2.665364	1.709789
H	-1.214600	3.210831	-2.483223
H	-0.531149	-2.194569	-4.540893
H	1.073224	-0.623316	0.054276

C14Me

C	0.065152	-0.045114	0.077779
C	0.332844	1.426307	-0.254750
C	-0.970266	1.956746	-0.880791
C	-1.636701	0.718510	-1.522861
C	-0.843824	-0.516346	-1.071685
C	-0.081923	-0.944693	-2.339578
C	-0.539071	-2.374936	-2.617153
C	-2.032097	-2.354999	-2.211569
C	-1.965529	-1.594590	-0.859132
C	-1.815452	-2.383586	0.435085
C	-2.335506	-1.399970	1.472860
C	-3.180483	-0.693328	-0.549528
C	-1.499216	0.756522	-3.046521
C	-1.696465	2.520292	0.354901
C	-0.519442	2.955406	1.225341
C	-2.682350	3.597072	0.089336
C	-2.768493	-3.718677	-2.310212
C	-4.162540	-3.648565	-1.664240
C	-1.937360	-4.866800	-1.714717
C	-3.006627	-4.039865	-3.798053
O	-0.627399	2.956785	-1.799276
O	-0.512736	-2.811588	0.708034
O	-2.045717	-1.393826	2.635160
O	-3.169459	-0.515836	0.886405
O	-3.004192	0.528550	-1.181905
O	-0.537547	-0.119849	-3.428811
O	-2.126031	1.425918	-3.809828
O	-0.512460	3.779799	2.087984
O	0.584728	2.228092	0.892813
O	-0.240378	-2.791073	-3.918553
H	-0.430645	-0.084449	1.052133
H	1.168410	1.561152	-0.948229
H	1.003342	-0.836791	-2.281028
H	0.001534	-3.040428	-1.933285
H	-2.551585	-1.678769	-2.913262
H	-2.511868	-3.232890	0.441264
H	-4.162862	-1.077797	-0.823350
H	-2.181647	1.684336	0.884805
H	-3.637609	3.346431	-0.354139
H	-2.524136	4.584640	0.501816
H	-4.153085	-3.475720	-0.583729
H	-4.687675	-4.594846	-1.829926
H	-4.764367	-2.858178	-2.128752
H	-1.578997	-4.675414	-0.698839
H	-1.059409	-5.061731	-2.339465
H	-2.536590	-5.783205	-1.695443
H	-3.617013	-3.263002	-4.273536

H	-3.548044	-4.988176	-3.885400
H	-2.065805	-4.126970	-4.342839
H	-0.418098	-2.825756	1.677311
H	-1.443978	3.381201	-2.112878
H	-0.541582	-2.087986	-4.518189
H	0.973774	-0.643886	0.160067

C3OH

C	0.055232	-0.029543	0.075439
C	0.365964	1.431896	-0.263304
C	-0.977731	1.976955	-0.909040
C	-1.639128	0.724126	-1.536536
C	-0.843765	-0.505476	-1.074231
C	-0.072777	-0.940916	-2.333864
C	-0.538021	-2.369548	-2.611502
C	-2.031907	-2.343045	-2.208338
C	-1.964585	-1.582198	-0.856270
C	-1.810485	-2.369045	0.438590
C	-2.329479	-1.384875	1.476867
C	-3.177246	-0.678532	-0.543893
C	-1.509202	0.737251	-3.064365
C	-1.671260	2.563736	0.329310
C	-0.498285	2.948389	1.222897
C	-2.611933	3.727362	0.054200
C	-2.772708	-3.704448	-2.306175
C	-4.164662	-3.630547	-1.656327
C	-1.943865	-4.855528	-1.713276
C	-3.014722	-4.023218	-3.793831
O	-0.581758	2.917525	-1.798497
O	-0.504591	-2.790086	0.709266
O	-2.036108	-1.378305	2.638343
O	-3.161745	-0.499043	0.891604
O	-2.998982	0.542764	-1.177060
O	-0.510223	-0.114495	-3.425542
O	-2.165108	1.349718	-3.845528
O	-0.489378	3.726401	2.125313
O	0.618283	2.237737	0.862647
O	-0.239719	-2.786325	-3.912253
H	-0.450488	-0.041366	1.045269
H	1.201354	1.539428	-0.960660
H	1.013096	-0.844131	-2.263550
H	-0.002283	-3.038202	-1.926878
H	-2.548832	-1.665952	-2.911116
H	-2.503412	-3.221052	0.446906
H	-4.161168	-1.060629	-0.815038
H	-2.213990	1.762609	0.849158
H	-3.479658	3.382987	-0.513082
H	-2.098476	4.504164	-0.516958
H	-2.946920	4.154599	1.001635
H	-4.151242	-3.458815	-0.575580
H	-4.693090	-4.575121	-1.821142
H	-4.765577	-2.838083	-2.118368
H	-1.582737	-4.665816	-0.697976
H	-1.068102	-5.053484	-2.340074
H	-2.546292	-5.769777	-1.692733
H	-3.625405	-3.245081	-4.266686
H	-3.557098	-4.970950	-3.881255
H	-2.075173	-4.110188	-4.340894
H	-0.413888	-2.820263	1.678558
H	-0.530007	-2.077891	-4.511260
H	0.958314	-0.634321	0.177499

C7OH

C	0.091624	-0.036468	0.038729
C	0.357355	1.439331	-0.282135
C	-0.951697	1.979031	-0.896676
C	-1.615630	0.741170	-1.545584
C	-0.832064	-0.501088	-1.099974
C	-0.091386	-0.938023	-2.384624
C	-0.512985	-2.399806	-2.585955
C	-2.037041	-2.354734	-2.205389
C	-1.956210	-1.569189	-0.868928
C	-1.815781	-2.345884	0.434110
C	-2.335472	-1.347435	1.457432
C	-3.172110	-0.660952	-0.576535
C	-1.472238	0.782859	-3.069070
C	-1.667255	2.535876	0.338179
C	-0.499228	2.920366	1.240240
C	-2.628940	3.687032	0.094511
C	-2.772681	-3.714576	-2.297486
C	-4.169354	-3.628884	-1.656644
C	-1.948816	-4.860393	-1.689543
C	-3.006821	-4.035810	-3.784553
O	-0.609514	2.978631	-1.822391
O	-0.519771	-2.783874	0.718793
O	-2.046566	-1.326962	2.619408
O	-3.169318	-0.473100	0.857318
O	-2.988198	0.551844	-1.219902
O	-0.575889	-0.141074	-3.468030
O	-2.056242	1.514364	-3.813901
O	-0.500438	3.691835	2.151238
O	0.615008	2.218149	0.877472
O	-0.329373	-2.888295	-3.835294
H	-0.394199	-0.081364	1.017922
H	1.189297	1.582242	-0.977983
H	0.993335	-0.813482	-2.338843
H	-0.015426	-3.019870	-1.818537
H	-2.513437	-1.687004	-2.939424
H	-2.520450	-3.188376	0.445392
H	-4.153905	-1.045320	-0.852844
H	-2.194810	1.720114	0.850726
H	-3.481229	3.351473	-0.503208
H	-2.123212	4.506080	-0.422135
H	-2.994495	4.064620	1.051581
H	-4.160548	-3.449534	-0.577484
H	-4.697622	-4.573710	-1.818204
H	-4.765483	-2.839193	-2.128845
H	-1.615739	-4.672267	-0.664629
H	-1.057082	-5.043920	-2.298191
H	-2.543467	-5.779621	-1.689204
H	-3.621517	-3.265025	-4.262060
H	-3.534449	-4.991323	-3.874940
H	-2.066068	-4.116049	-4.334835
H	-0.423843	-2.770574	1.688233
H	-1.391126	3.199423	-2.356292
H	1.000875	-0.636230	0.109047

C10OH

C	0.067719	-0.051726	0.051631
C	0.355667	1.423969	-0.255984
C	-0.937044	1.979635	-0.892587
C	-1.608742	0.751739	-1.553653
C	-0.838105	-0.496552	-1.109006
C	-0.085381	-0.940580	-2.376243
C	-0.543615	-2.374295	-2.632202
C	-2.033984	-2.351271	-2.219831
C	-1.970646	-1.559458	-0.887973
C	-1.783450	-2.307463	0.432783
C	-2.424915	-1.362320	1.477847
C	-3.180077	-0.651410	-0.603662
C	-1.456673	0.798508	-3.075872
C	-1.666375	2.545437	0.329202
C	-0.509562	2.914459	1.251849
C	-2.608674	3.709251	0.069867
C	-2.767167	-3.719432	-2.287951
C	-4.132961	-3.656525	-1.584723
C	-1.911688	-4.867175	-1.727578
C	-3.057762	-4.042266	-3.765728
O	-0.563850	2.971785	-1.814768
O	-0.531823	-2.721850	0.755316
O	-2.272850	-1.378009	2.657328
O	-3.209916	-0.471023	0.823723
O	-2.982241	0.569729	-1.236826
O	-0.551523	-0.127140	-3.469718
O	-2.035998	1.521491	-3.830623
O	-0.515663	3.687858	2.160640
O	0.601457	2.196092	0.908824
O	-0.254922	-2.814597	-3.927386
H	-0.438937	-0.095567	1.021936
H	1.201291	1.557464	-0.937068
H	1.000409	-0.830831	-2.327741
H	0.003114	-3.026785	-1.940987
H	-2.556961	-1.691148	-2.933945
H	-2.395679	-3.227189	0.474082

H	-4.156938	-1.028955	-0.906672
H	-2.214537	1.735727	0.829848
H	-3.452168	3.386697	-0.547296
H	-2.082645	4.526050	-0.430225
H	-2.990517	4.086669	1.020551
H	-4.081206	-3.454134	-0.509299
H	-4.648839	-4.614928	-1.701834
H	-4.768669	-2.887999	-2.039866
H	-1.549905	-4.698330	-0.708702
H	-1.036428	-5.035726	-2.363195
H	-2.497321	-5.792235	-1.721428
H	-3.690277	-3.269510	-4.217897
H	-3.593803	-4.994865	-3.836245
H	-2.135194	-4.121312	-4.342768
H	-1.346030	3.242043	-2.324165
H	-0.561117	-2.124600	-4.539510
H	0.972545	-0.658606	0.138064

1M

C	0.081266	0.015474	0.034455
C	0.347519	1.486069	-0.387005
C	-1.010984	1.997405	-0.908765
C	-1.666512	0.758725	-1.532001
C	-0.867680	-0.477364	-1.079289
C	-0.105688	-0.918193	-2.339605
C	-0.536547	-2.360136	-2.585127
C	-2.029002	-2.349955	-2.188811
C	-1.987305	-1.554695	-0.848327
C	-1.908763	-2.331889	0.458667
C	-2.629383	-1.434304	1.460323
C	-3.241541	-0.673276	-0.617417
C	-1.517254	0.782694	-3.059503
C	-1.647472	2.561517	0.363310
C	-0.426452	2.907685	1.214389
C	-2.566615	3.758097	0.165537
C	-2.748798	-3.724541	-2.276429
C	-4.152887	-3.673259	-1.647745
C	-1.901345	-4.857323	-1.672178
C	-2.972437	-4.065073	-3.763194
O	-0.603412	-2.665546	0.868841
O	-2.557113	-1.536248	2.650963
O	-3.411989	-0.555174	0.802781
O	-3.030290	0.571698	-1.195850
O	-0.571684	-0.119572	-3.439025
O	-2.095452	1.481289	-3.833744
O	-0.380779	3.619680	2.171228
O	0.694844	2.304700	0.706450
O	-0.219610	-2.802364	-3.873873
O	-0.534915	0.059396	1.293881
H	1.150863	1.541947	-1.127645
H	0.979678	-0.797550	-2.286209
H	0.010510	-3.000141	-1.881610
H	-2.548962	-1.692348	-2.907472
H	-2.514911	-3.240316	0.380418
H	-4.173686	-1.073433	-1.018434
H	-2.183356	1.768689	0.897751
H	-3.455908	3.458553	-0.394619
H	-2.058680	4.555918	-0.385437
H	-2.871885	4.155141	1.136251
H	-4.172261	-3.452417	-0.576312
H	-4.644449	-4.642300	-1.782009
H	-4.772658	-2.924129	-2.154231
H	-1.542208	-4.653743	-0.658870
H	-1.021657	-5.043881	-2.297033
H	-2.486839	-5.782189	-1.643488
H	-3.583643	-3.297778	-4.252592
H	-3.508126	-5.017301	-3.843322
H	-2.027217	-4.154118	-4.299722
H	-0.687337	-3.122336	1.722891
H	-0.533028	-2.121411	-4.492631
H	1.001785	-0.576906	0.067678
H	-0.893362	2.796369	-1.648369
H	-0.291127	-0.742811	1.778289

C1

C	0.112159	0.088303	-0.025327
C	0.351273	1.548850	-0.380332
C	-1.016967	2.023396	-0.930192
C	-1.620529	0.776958	-1.588268
C	-0.834445	-0.461707	-1.083600
C	-0.069089	-0.944047	-2.335114
C	-0.524576	-2.380924	-2.559755
C	-2.021222	-2.334925	-2.179095
C	-1.973070	-1.526580	-0.848240
C	-1.935031	-2.286167	0.469669
C	-2.550718	-1.303631	1.463271
C	-3.200879	-0.612525	-0.632492
C	-1.415552	0.791365	-3.107467
C	-1.691083	2.499180	0.358498
C	-0.503202	2.936458	1.210436
C	-2.735317	3.593332	0.205720
C	-2.774071	-3.692323	-2.260375
C	-4.189991	-3.596852	-1.664578
C	-1.972451	-4.836437	-1.618046
C	-2.974413	-4.049973	-3.746552
O	-0.657925	-2.730511	0.870872
O	-2.439886	-1.357575	2.652066
O	-3.281513	-0.389316	0.790437
O	-3.000350	0.575217	-1.311399
O	-0.509350	-0.159204	-3.455949
O	-1.934020	1.517754	-3.898828
O	-0.506370	3.676580	2.147064
O	0.642013	2.357565	0.741361
O	-0.205540	-2.851317	-3.837852
O	-0.025742	-0.142019	1.297634
H	1.170844	1.664649	-1.098092
H	1.014693	-0.836433	-2.260489
H	0.003515	-3.019043	-1.840506
H	-2.518805	-1.671758	-2.907954
H	-2.618939	-3.139259	0.416246
H	-4.160956	-1.020945	-0.949952
H	-2.133656	1.642637	0.883931
H	-3.599777	3.213794	-0.344080
H	-2.323693	4.450326	-0.336243
H	-3.057733	3.937129	1.191078
H	-4.226854	-3.387720	-0.591221
H	-4.711000	-4.547266	-1.818904
H	-4.772041	-2.822231	-2.177705
H	-1.647486	-4.631287	-0.593650
H	-1.074420	-5.046394	-2.208234
H	-2.577852	-5.748739	-1.603694
H	-3.553316	-3.273994	-4.260970
H	-3.534823	-4.988056	-3.823371
H	-2.021003	-4.173331	-4.260831
H	-0.767174	-3.178189	1.727877
H	-0.488461	-2.170115	-4.470852
H	-0.930786	2.850408	-1.642893
H	-0.040953	-1.109176	1.435476

C2

C	0.079229	-0.027327	0.095421
C	0.261443	1.440939	-0.245679
C	-0.993626	1.983981	-0.879659
C	-1.678555	0.742475	-1.467867
C	-0.868989	-0.494441	-1.034285
C	-0.109822	-0.905695	-2.305099
C	-0.533543	-2.346934	-2.570465
C	-2.027947	-2.345301	-2.175835
C	-1.984449	-1.577962	-0.818686
C	-1.897805	-2.394166	0.464258
C	-2.618076	-1.533503	1.497379
C	-3.238762	-0.704785	-0.551619
C	-1.572599	0.760508	-2.998014
C	-1.645123	2.666229	0.336062
C	-0.413854	3.069223	1.146103
C	-2.572729	3.833034	0.045313
C	-2.748372	-3.717416	-2.293845
C	-4.150639	-3.680725	-1.659855
C	-1.899220	-4.866081	-1.722840
C	-2.977312	-4.020337	-3.788043
O	-0.590452	-2.742942	0.855587
O	-2.545045	-1.674705	2.683810
O	-3.410294	-0.640435	0.869942
O	-3.025640	0.567141	-1.074296

O	-0.597788	-0.100506	-3.391864
O	-2.200901	1.428723	-3.760469
O	-0.306302	3.915409	1.975432
O	0.657307	2.267961	0.771200
O	-0.215888	-2.771512	-3.864801
O	-0.526207	-0.049146	1.368990
H	0.972835	-0.766153	-2.257022
H	0.013831	-2.995457	-1.875022
H	-2.548576	-1.674920	-2.881707
H	-2.503500	-3.299811	0.358285
H	-4.170382	-1.088438	-0.969922
H	-2.163762	1.900797	0.930190
H	-3.457540	3.481331	-0.490580
H	-2.069339	4.587617	-0.566781
H	-2.885280	4.305450	0.979428
H	-4.167563	-3.484301	-0.583597
H	-4.642849	-4.646318	-1.815064
H	-4.771544	-2.919978	-2.147308
H	-1.539277	-4.692205	-0.704383
H	-1.019874	-5.032924	-2.353708
H	-2.483767	-5.792010	-1.720730
H	-3.590576	-3.241258	-4.255712
H	-3.512686	-4.970624	-3.890337
H	-2.033718	-4.095293	-4.329577
H	-0.670119	-3.234629	1.690412
H	-0.526989	-2.080682	-4.473756
H	1.012434	-0.601468	0.097155
H	-0.796735	2.738513	-1.652166
H	-0.270670	-0.873863	1.809484

C3

C	0.019327	-0.039505	0.117989
C	0.285877	1.438010	-0.296762
C	-1.086736	1.873145	-0.733922
C	-1.754642	0.714835	-1.400546
C	-0.910990	-0.522765	-1.017155
C	-0.156305	-0.883602	-2.303490
C	-0.562927	-2.321327	-2.612222
C	-2.046017	-2.369533	-2.183971
C	-2.003039	-1.628102	-0.812204
C	-1.879139	-2.447229	0.466272
C	-2.626972	-1.617274	1.504007
C	-3.282048	-0.792152	-0.531502
C	-1.638551	0.800138	-2.934123
C	-1.656139	2.722339	0.346023
C	-0.428144	2.988483	1.223658
C	-2.335874	4.023207	-0.088479
C	-2.728194	-3.760062	-2.311437
C	-4.119216	-3.772617	-1.652637
C	-1.835931	-4.888919	-1.766970
C	-2.972775	-4.051092	-3.805211
O	-0.558039	-2.731733	0.856783
O	-2.523527	-1.750045	2.690439
O	-3.467041	-0.764229	0.888953
O	-3.101757	0.493720	-1.031241
O	-0.650036	-0.037664	-3.355587
O	-2.277478	1.478230	-3.676445
O	-0.349391	3.737558	2.148875
O	0.648764	2.262194	0.786482
O	-0.266293	-2.691659	-3.928012
O	-0.670432	0.033402	1.338980
H	1.066008	1.502621	-1.065357
H	0.927236	-0.746023	-2.256625
H	0.015482	-2.981385	-1.953780
H	-2.599224	-1.699192	-2.865591
H	-2.446062	-3.378640	0.363058
H	-4.200187	-1.192485	-0.963417
H	-2.359802	2.126620	0.948091
H	-3.236176	3.798279	-0.664890
H	-1.664834	4.621786	-0.710028
H	-2.607220	4.604729	0.795446
H	-4.122082	-3.583591	-0.574947
H	-4.583031	-4.752446	-1.806030
H	-4.773043	-3.029171	-2.123356
H	-1.457907	-4.710708	-0.755685
H	-0.966946	-5.029485	-2.418295
H	-2.395187	-5.830259	-1.757325
H	-3.616382	-3.284718	-4.252709
H	-3.481836	-5.015386	-3.910803
H	-2.036783	-4.090758	-4.363439
H	-0.615378	-3.164671	1.725561
H	-0.603470	-1.985267	-4.504367
H	0.929248	-0.641283	0.196548
H	-0.372140	-0.683664	1.915972

C6

C	0.102485	0.019873	-0.025678
C	0.344359	1.501576	-0.421040
C	-1.030728	2.015384	-0.896052
C	-1.667120	0.794110	-1.563528
C	-0.883446	-0.457237	-1.120852
C	-0.201613	-0.917552	-2.384024
C	-0.527993	-2.366625	-2.616719
C	-2.032439	-2.369552	-2.196598
C	-2.004502	-1.537774	-0.873142
C	-1.947010	-2.287338	0.450333
C	-2.648595	-1.351636	1.429712
C	-3.253352	-0.640926	-0.671455
C	-1.449394	0.868628	-3.084279
C	-1.648204	2.525754	0.407164
C	-0.409986	2.866483	1.237566
C	-2.587990	3.714827	0.269105
C	-2.742616	-3.749982	-2.253531
C	-4.158185	-3.681843	-1.651140
C	-1.899925	-4.858308	-1.600206
C	-2.942568	-4.139093	-3.731801
O	-0.653600	-2.655836	0.868815
O	-2.590195	-1.426948	2.622058
O	-3.409197	-0.470049	0.744865
O	-3.043744	0.582483	-1.297680
O	-0.567812	-0.121339	-3.461140
O	-1.911372	1.641940	-3.860774
O	-0.352265	3.551808	2.213032
O	0.707564	2.299920	0.682432
O	-0.236606	-2.818780	-3.904827
O	-0.470680	0.034071	1.254107
H	1.129028	1.576080	-1.179616
H	0.036438	-2.988551	-1.911777
H	-2.552168	-1.731165	-2.933210
H	-2.578551	-3.179233	0.385663
H	-4.190595	-1.048069	-1.053893
H	-2.159844	1.710494	0.931060
H	-3.487660	3.420584	-0.277185
H	-2.104984	4.536369	-0.269268
H	-2.873429	4.077436	1.259128
H	-4.198412	-3.437130	-0.585753
H	-4.649132	-4.652905	-1.772784
H	-4.766335	-2.943141	-2.186468
H	-1.556248	-4.620472	-0.589366
H	-1.010029	-5.060317	-2.205596
H	-2.481114	-5.785055	-1.551423
H	-3.545202	-3.388006	-4.255930
H	-3.478652	-5.092774	-3.787869
H	-1.989975	-4.245511	-4.251258
H	-0.753465	-3.115352	1.719611
H	-0.525729	-2.131629	-4.527661
H	1.026119	-0.567087	-0.044029
H	-0.935919	2.844068	-1.605701
H	-0.243838	-0.800096	1.691023

C7

C	0.072425	0.011464	-0.016788
C	0.335568	1.485933	-0.424113
C	-1.031720	2.007953	-0.907383
C	-1.696233	0.782308	-1.549567
C	-0.900253	-0.466302	-1.119908
C	-0.152324	-0.896148	-2.396573
C	-0.696234	-2.241696	-2.701304
C	-2.116230	-2.324489	-2.220048
C	-2.021240	-1.534052	-0.873744
C	-1.928678	-2.312857	0.431752
C	-2.634690	-1.412359	1.442464
C	-3.267025	-0.645795	-0.625927
C	-1.523973	0.857715	-3.073215

C	-1.641776	2.550521	0.386598
C	-0.401536	2.881734	1.216419
C	-2.561237	3.752621	0.226774
C	-2.782018	-3.728433	-2.275501
C	-4.170632	-3.715568	-1.611200
C	-1.866083	-4.811654	-1.683708
C	-3.040069	-4.087341	-3.752564
O	-0.622628	-2.654003	0.832738
O	-2.553133	-1.519249	2.632100
O	-3.414882	-0.522498	0.796170
O	-3.059572	0.594137	-1.215278
O	-0.512908	0.036806	-3.453581
O	-2.120833	1.560391	-3.831246
O	-0.333892	3.577873	2.183680
O	0.708718	2.288610	0.672962
O	-0.315835	-2.898443	-3.823676
O	-0.515746	0.045743	1.255526
H	1.121453	1.550109	-1.182802
H	0.939916	-0.851312	-2.316985
H	-2.733894	-1.685941	-2.888314
H	-2.538390	-3.218308	0.361038
H	-4.206343	-1.046498	-1.011653
H	-2.169060	1.751157	0.919565
H	-3.461914	3.464362	-0.320977
H	-2.062058	4.558268	-0.320835
H	-2.846575	4.134241	1.209681
H	-4.174998	-3.482649	-0.542725
H	-4.632738	-4.701671	-1.723444
H	-4.825907	-2.992472	-2.111069
H	-1.514400	-4.590975	-0.672528
H	-0.978072	-4.930182	-2.312062
H	-2.393464	-5.771241	-1.660479
H	-3.687713	-3.342012	-4.227835
H	-3.549868	-5.055457	-3.805985
H	-2.115251	-4.154564	-4.325625
H	-0.707119	-3.115386	1.684263
H	0.486371	-2.493205	-4.187338
H	0.989209	-0.587420	-0.008176
H	-0.926163	2.822293	-1.631917
H	-0.285606	-0.776890	1.712164

C8

C	0.082934	0.044459	0.101346
C	0.330906	1.492191	-0.395861
C	-1.030884	1.952329	-0.958876
C	-1.682347	0.664821	-1.488291
C	-0.832820	-0.521221	-0.999654
C	-0.058348	-0.967764	-2.251671
C	-0.423907	-2.438410	-2.457150
C	-1.855074	-2.502171	-1.963552
C	-1.911460	-1.626089	-0.731817
C	-1.804308	-2.373989	0.602322
C	-2.592081	-1.502203	1.574178
C	-3.207797	-0.786044	-0.524602
C	-1.598681	0.590872	-3.017575
C	-1.679531	2.599716	0.266987
C	-0.470197	3.006040	1.106404
C	-2.603790	3.774449	-0.017597
C	-2.752965	-3.674462	-2.309591
C	-4.119997	-3.655962	-1.604827
C	-2.008142	-4.978998	-1.951096
C	-3.045690	-3.682210	-3.826139
O	-0.483687	-2.603721	1.028011
O	-2.518305	-1.564747	2.767777
O	-3.418646	-0.688406	0.890724
O	-3.019427	0.476284	-1.072616
O	-0.575034	-0.233832	-3.371854
O	-2.271123	1.170300	-3.813419
O	-0.438633	3.777576	2.016708
O	0.658263	2.374855	0.653229
O	-0.154262	-2.883113	-3.756624
O	-0.578796	0.141156	1.336562
H	1.139615	1.519583	-1.132152
H	1.019917	-0.793117	-2.212898
H	0.211376	-3.043854	-1.788661
H	-2.341039	-3.328563	0.519352
H	-4.115678	-1.207129	-0.950521
H	-2.214751	1.839650	0.847322
H	-3.486928	3.432563	-0.563118
H	-2.096190	4.536866	-0.616919
H	-2.919245	4.233866	0.921852
H	-4.064901	-3.542735	-0.517243
H	-4.629287	-4.604398	-1.802146
H	-4.760313	-2.861498	-2.000749
H	-1.785927	-5.040671	-0.879233
H	-1.066063	-5.047742	-2.503452
H	-2.623055	-5.844827	-2.219596
H	-3.458997	-2.718370	-4.143604
H	-3.785783	-4.458709	-4.049346
H	-2.145560	-3.890428	-4.404683
H	-0.540148	-3.057156	1.885727
H	-0.536215	-2.224218	-4.360951
H	1.012405	-0.526545	0.193046
H	-0.915911	2.696396	-1.754039
H	-0.311275	-0.611294	1.884255

C8tBu

C	0.089172	0.020577	0.028849
C	0.372390	1.493311	-0.374478
C	-0.975602	2.023268	-0.905198
C	-1.634584	0.799040	-1.550956
C	-0.858894	-0.452036	-1.094484
C	-0.096425	-0.900264	-2.353923
C	-0.537475	-2.338226	-2.598488
C	-2.026087	-2.324133	-2.196718
C	-1.994647	-1.511538	-0.869208
C	-1.972354	-2.291829	0.437899
C	-2.686275	-1.376104	1.424552
C	-3.242222	-0.613063	-0.664730
C	-1.464752	0.833880	-3.075998
C	-1.620656	2.578004	0.366590
C	-0.405325	2.906247	1.232760
C	-2.529463	3.783064	0.173105
C	-2.724092	-3.717020	-2.225076
C	-4.039303	-3.680110	-1.496869
C	-1.811394	-4.821514	-1.665911
C	-3.045073	-4.073510	-3.697356
O	-0.691364	-2.671315	0.884925
O	-2.635224	-1.475001	2.616655
O	-3.433143	-0.478936	0.750855
O	-3.007800	0.625532	-1.246561
O	-0.550588	-0.099540	-3.455830
O	-2.010183	1.559632	-3.848977
O	-0.362916	3.609555	2.196126
O	0.715766	2.297952	0.730536
O	-0.238139	-2.790007	-3.888487
O	-0.531147	0.055674	1.285906
H	1.184024	1.549053	-1.106133
H	0.989845	-0.788434	-2.295474
H	0.011050	-2.978525	-1.897037
H	-2.555238	-1.692506	-2.931028
H	-2.621112	-3.171583	0.328361
H	-4.170907	-1.009595	-1.076648
H	-2.167198	1.783384	0.887454
H	-3.415293	3.495790	-0.398809
H	-2.010648	4.583211	-0.364197
H	-2.841345	4.171658	1.145135
H	-4.367794	-4.545361	-0.930083
H	-4.818680	-3.011046	-1.850519
H	-1.403773	-4.587436	-0.677137
H	-0.967109	-4.992345	-2.341038
H	-2.371177	-5.759409	-1.592874
H	-3.720449	-3.332426	-4.138488
H	-3.541224	-5.048311	-3.739975
H	-2.130656	-4.117425	-4.293198
H	-0.816225	-3.105862	1.745946
H	-0.538105	-2.102426	-4.506571
H	1.004486	-0.580865	0.059335
H	-0.842470	2.829804	-1.633905
H	-0.324495	-0.771610	1.745594

C10

C	0.092157	-0.049062	0.007164
C	0.374160	1.440792	-0.327361

C	-0.958894	1.993892	-0.879269
C	-1.651969	0.777288	-1.515365
C	-0.858139	-0.473874	-1.119389
C	-0.121655	-0.889597	-2.401646
C	-0.538119	-2.338265	-2.652370
C	-2.020978	-2.360320	-2.230372
C	-1.984230	-1.554578	-0.895180
C	-1.897869	-2.251383	0.405373
C	-2.756538	-1.609455	1.376891
C	-3.252071	-0.683743	-0.660376
C	-1.549919	0.828877	-3.046861
C	-1.629719	2.574152	0.365701
C	-0.450769	2.854004	1.285905
C	-2.497354	3.803491	0.138878
C	-2.721950	-3.745922	-2.273859
C	-4.075267	-3.702662	-1.549751
C	-1.849476	-4.855438	-1.672429
C	-3.003813	-4.103229	-3.743969
O	-0.848636	-2.968087	0.815399
O	-2.788624	-1.834317	2.561620
O	-3.524813	-0.676364	0.738336
O	-3.000134	0.610396	-1.127122
O	-0.633772	-0.082288	-3.473635
O	-2.140204	1.553049	-3.787750
O	-0.418872	3.534526	2.265131
O	0.665900	2.172476	0.852393
O	-0.242882	-2.765553	-3.950826
O	-0.573768	-0.118315	1.244236
H	1.217375	1.550615	-1.014895
H	0.963302	-0.753821	-2.377879
H	0.035798	-2.975509	-1.967876
H	-2.566104	-1.718332	-2.944762
H	-4.147576	-1.051027	-1.163417
H	-2.220614	1.792212	0.858852
H	-3.363894	3.537513	-0.471233
H	-1.936009	4.590371	-0.374382
H	-2.842871	4.198137	1.096943
H	-3.978033	-3.464987	-0.484967
H	-4.558648	-4.682399	-1.622769
H	-4.751413	-2.973866	-2.011991
H	-1.572096	-4.658493	-0.633902
H	-0.933457	-4.992769	-2.256849
H	-2.399409	-5.802115	-1.700397
H	-3.638614	-3.345379	-4.218450
H	-3.535261	-5.059722	-3.793609
H	-2.078973	-4.192850	-4.316824
H	-0.923204	-3.022909	1.789991
H	-0.617620	-2.107219	-4.559489
H	1.005049	-0.660027	0.020611
H	-0.797904	2.787021	-1.616645
H	-0.013444	0.312408	1.910233

C12

C	0.088215	0.029185	0.058596
C	0.342542	1.496419	-0.369504
C	-1.012768	1.983364	-0.925394
C	-1.648949	0.723215	-1.530074
C	-0.816201	-0.492469	-1.071082
C	-0.059675	-0.931809	-2.333271
C	-0.497433	-2.376294	-2.578556
C	-1.983833	-2.352980	-2.167941
C	-1.920625	-1.582113	-0.814169
C	-1.838657	-2.380437	0.484283
C	-2.660471	-1.554880	1.475197
C	-3.135397	-0.710477	-0.559200
C	-1.525458	0.727418	-3.056334
C	-1.675531	2.575526	0.321311
C	-0.474306	2.940781	1.191824
C	-2.592603	3.765317	0.078309
C	-2.753285	-3.694932	-2.270703
C	-4.129814	-3.588856	-1.590457
C	-1.932492	-4.878533	-1.732822
C	-3.035091	-3.976154	-3.759501
O	-0.530667	-2.637426	0.940308
O	-2.656714	-1.679110	2.662794
O	-3.454281	-0.681126	0.793900
O	-3.009477	0.514847	-1.132795
O	-0.538396	-0.132576	-3.429723
O	-2.141502	1.388654	-3.833960
O	-0.453018	3.668243	2.137682
O	0.658903	2.330980	0.721390
O	-0.199070	-2.816914	-3.872014
O	-0.594370	0.070234	1.288299
H	1.158532	1.554420	-1.096032
H	1.025655	-0.809022	-2.290303
H	0.055303	-3.022163	-1.884364
H	-2.500806	-1.670157	-2.863104
H	-2.379457	-3.322922	0.363516
H	-2.219875	1.794407	0.863583
H	-3.471175	3.451749	-0.491028
H	-2.075447	4.551074	-0.481287
H	-2.916497	4.184017	1.033736
H	-4.096112	-3.475776	-0.502919
H	-4.710233	-4.493470	-1.800229
H	-4.686861	-2.732702	-1.987430
H	-1.543923	-4.730083	-0.720366
H	-1.073433	-5.067883	-2.385379
H	-2.548870	-5.783585	-1.724831
H	-3.651200	-3.181900	-4.196533
H	-3.587122	-4.917199	-3.857245
H	-2.109508	-4.058872	-4.331388
H	-0.610022	-3.165476	1.751793
H	-0.523342	-2.136415	-4.485672
H	1.015981	-0.546176	0.140485
H	-0.889863	2.764309	-1.683076
H	-0.334359	-0.712814	1.796324

C14

C	0.124015	0.056157	-0.011133
C	0.479171	1.499579	-0.470916
C	-0.855654	2.048374	-1.034301
C	-1.555058	0.813882	-1.631080
C	-0.811825	-0.436082	-1.131596
C	-0.043543	-0.941161	-2.362126
C	-0.518364	-2.374950	-2.570636
C	-2.016561	-2.303188	-2.203379
C	-1.974131	-1.463962	-0.889371
C	-1.952006	-2.196857	0.445505
C	-2.667180	-1.243160	1.397698
C	-3.202704	-0.533591	-0.717797
C	-1.371501	0.793396	-3.156157
C	-1.413509	2.686457	0.193222
C	-0.309513	2.911542	1.122361
C	-2.793223	3.150842	0.452210
C	-2.779706	-3.655823	-2.258227
C	-4.194013	-3.535555	-1.662650
C	-1.982385	-4.792782	-1.596132
C	-2.984327	-4.042588	-3.736390
O	-0.670452	-2.561767	0.898982
O	-2.627961	-1.306297	2.592418
O	-3.413511	-0.370356	0.690273
O	-2.930307	0.687672	-1.323154
O	-0.463679	-0.161393	-3.493737
O	-1.899407	1.500937	-3.957921
O	-0.302900	3.536566	2.149124
O	0.835038	2.320290	0.615482
O	-0.193001	-2.868661	-3.838524
O	-0.549527	0.177241	1.213423
H	1.298696	1.499383	-1.194829
H	1.044204	-0.854749	-2.292632
H	-0.005488	-3.009600	-1.837219
H	-2.500822	-1.654115	-2.954114
H	-2.588184	-3.085921	0.382369
H	-4.135228	-0.911918	-1.138864
H	-3.426494	2.308041	0.756812
H	-3.244758	3.583334	-0.446660
H	-2.783981	3.887165	1.259716
H	-4.228526	-3.272744	-0.601214
H	-4.714221	-4.492850	-1.770855
H	-4.778274	-2.787151	-2.210783
H	-1.635309	-4.563840	-0.584066
H	-1.098062	-5.030917	-2.196469
H	-2.598963	-5.696265	-1.545741
H	-3.559169	-3.273858	-4.266020

H	-3.550233	-4.978822	-3.793939
H	-2.031993	-4.182282	-4.248925
H	-0.790721	-2.960936	1.777344
H	-0.475541	-2.200260	-4.485135
H	1.013011	-0.575368	0.083833
H	-0.708968	2.809771	-1.814947
H	-0.306640	-0.572522	1.775327

C14Me

C	0.067274	0.011394	0.043858
C	0.322353	1.485864	-0.370227
C	-1.038744	1.991107	-0.890853
C	-1.689069	0.749832	-1.514500
C	-0.879888	-0.482561	-1.070720
C	-0.118036	-0.910716	-2.335457
C	-0.539717	-2.354311	-2.588042
C	-2.031213	-2.356360	-2.188034
C	-1.991400	-1.568958	-0.842888
C	-1.903114	-2.353756	0.458883
C	-2.628282	-1.468468	1.468183
C	-3.251252	-0.697715	-0.602685
C	-1.546877	0.781141	-3.042578
C	-1.676535	2.551695	0.387746
C	-0.452695	2.897898	1.241066
C	-2.577926	3.719081	0.201187
C	-2.741987	-3.735220	-2.282113
C	-4.144246	-3.697580	-1.648435
C	-1.884758	-4.866043	-1.688093
C	-2.968151	-4.067550	-3.770345
O	-0.593645	-2.678235	0.863744
O	-2.551015	-1.576926	2.657925
O	-3.419396	-0.591196	0.818539
O	-3.049457	0.553362	-1.171892
O	-0.592896	-0.109273	-3.429092
O	-2.136866	1.475501	-3.811550
O	-0.410264	3.604429	2.201227
O	0.666177	2.299797	0.728325
O	-0.223115	-2.787085	-3.879981
O	-0.547900	0.045611	1.304188
H	1.124544	1.552221	-1.111083
H	0.966606	-0.782831	-2.284489
H	0.013461	-2.994491	-1.889509
H	-2.557381	-1.698095	-2.901566
H	-2.501310	-3.266974	0.376424
H	-4.181428	-1.101230	-1.004808
H	-2.196577	1.738998	0.921295
H	-3.370164	3.661040	-0.534552
H	-2.619651	4.478013	0.971928
H	-4.161596	-3.483242	-0.575630
H	-4.629409	-4.669249	-1.786832
H	-4.771049	-2.949902	-2.148364
H	-1.523794	-4.666789	-0.674556
H	-1.005810	-5.042377	-2.316946
H	-2.463754	-5.795095	-1.663666
H	-3.586188	-3.301297	-4.252725
H	-3.497551	-5.022889	-3.854947
H	-2.024069	-4.146673	-4.310447
H	-0.671343	-3.141600	1.714840
H	-0.542830	-2.104940	-4.494200
H	0.991972	-0.574499	0.073436
H	-0.929240	2.795450	-1.624425
H	-0.298862	-0.757195	1.785085

C1OH

C	0.058928	0.010081	0.038734
C	0.365424	1.494161	-0.394169
C	-0.995091	1.990645	-0.914856
C	-1.641464	0.756296	-1.558921
C	-0.864495	-0.487341	-1.091553
C	-0.088747	-0.943072	-2.337195
C	-0.540048	-2.378846	-2.585270
C	-2.035959	-2.347316	-2.202928
C	-1.994493	-1.551426	-0.864432
C	-1.905977	-2.322954	0.447705
C	-2.607455	-1.410928	1.447550
C	-3.230781	-0.651623	-0.632889
C	-1.456556	0.784500	-3.082246
C	-1.630574	2.507904	0.376567
C	-0.408378	2.919861	1.196249
C	-2.636927	3.638726	0.223380
C	-2.768553	-3.715118	-2.286971
C	-4.172652	-3.644161	-1.661337
C	-1.934868	-4.852354	-1.671826
C	-2.990261	-4.059488	-3.772836
O	-0.603851	-2.663105	0.837707
O	-2.555280	-1.514943	2.636557
O	-3.359602	-0.496935	0.786404
O	-3.012015	0.578767	-1.247351
O	-0.524760	-0.137026	-3.444399
O	-2.000098	1.503440	-3.862923
O	-0.361234	3.659778	2.129216
O	0.718286	2.318037	0.686830
O	-0.215423	-2.827142	-3.869706
O	-0.493182	0.013988	1.281924
H	1.171981	1.516060	-1.131633
H	0.997941	-0.840302	-2.268584
H	-0.009348	-3.025198	-1.875338
H	-2.542855	-1.686960	-2.928821
H	-2.525751	-3.225189	0.387486
H	-4.177992	-1.042224	-1.006607
H	-2.106000	1.675876	0.917811
H	-3.518290	3.282558	-0.314659
H	-2.199691	4.474948	-0.330577
H	-2.938482	4.001524	1.208315
H	-4.186588	-3.426569	-0.589326
H	-4.678194	-4.605634	-1.797856
H	-4.780944	-2.884822	-2.166981
H	-1.575026	-4.645532	-0.659493
H	-1.057526	-5.055403	-2.295015
H	-2.532067	-5.769524	-1.637144
H	-3.594625	-3.289988	-4.267531
H	-3.532176	-5.008302	-3.850847
H	-2.044001	-4.158308	-4.306100
H	-0.598100	-2.659571	1.812023
H	-0.528082	-2.152281	-4.495225
H	0.994640	-0.567151	0.121163
H	-0.888354	2.809422	-1.634152

C7OH

C	0.087965	0.016818	0.015334
C	0.346898	1.490606	-0.400835
C	-1.016853	1.999778	-0.911188
C	-1.668302	0.762886	-1.539461
C	-0.868344	-0.474358	-1.092595
C	-0.119852	-0.911653	-2.369995
C	-0.508902	-2.385195	-2.548342
C	-2.032921	-2.357290	-2.180333
C	-1.981738	-1.549745	-0.848250
C	-1.907039	-2.326001	0.459523
C	-2.624939	-1.423817	1.458327
C	-3.240762	-0.670788	-0.623125
C	-1.518729	0.793190	-3.067582
C	-1.647245	2.556550	0.367066
C	-0.422364	2.899562	1.214030
C	-2.567500	3.754068	0.179390
C	-2.746624	-3.729742	-2.276705
C	-4.155463	-3.674865	-1.656995
C	-1.905692	-4.864307	-1.668586
C	-2.958061	-4.055242	-3.766918
O	-0.605963	-2.669445	0.872893
O	-2.551049	-1.521958	2.648843
O	-3.408600	-0.548328	0.797359
O	-3.033872	0.569735	-1.207099
O	-0.609152	-0.134833	-3.458393
O	-2.077981	1.522227	-3.828036
O	-0.372947	3.604361	2.175875
O	0.698114	2.303266	0.695426
O	-0.303027	-2.886774	-3.788653
O	-0.523118	0.053780	1.277883
H	1.145426	1.553320	-1.146165
H	0.964515	-0.774699	-2.321323
H	-0.003705	-2.979190	-1.764497
H	-2.509991	-1.702888	-2.925680
H	-2.520321	-3.229769	0.379772

H	-4.172373	-1.076614	-1.020099
H	-2.180718	1.761681	0.900873
H	-3.458799	3.457442	-0.379114
H	-2.061885	4.554745	-0.369465
H	-2.869123	4.145881	1.153361
H	-4.177476	-3.463318	-0.583930
H	-4.648033	-4.641462	-1.801886
H	-4.769752	-2.920288	-2.161367
H	-1.580383	-4.676833	-0.641128
H	-1.007067	-5.027612	-2.272840
H	-2.483040	-5.794376	-1.675528
H	-3.576498	-3.292898	-4.252608
H	-3.471915	-5.017714	-3.862949
H	-2.009938	-4.123864	-4.306297
H	-0.694923	-3.109734	1.735361
H	1.010378	-0.573197	0.041643
H	-0.906864	2.801806	-1.648454
H	-0.267168	-0.744472	1.762303

C10OH

C	0.077761	-0.032544	0.024806
C	0.353947	1.450793	-0.336021
C	-0.983638	1.983487	-0.895459
C	-1.653554	0.753821	-1.529827
C	-0.866892	-0.492061	-1.093460
C	-0.109544	-0.929286	-2.358293
C	-0.544577	-2.371068	-2.601985
C	-2.034929	-2.359532	-2.200939
C	-1.991001	-1.564020	-0.863604
C	-1.858539	-2.338845	0.452882
C	-2.683277	-1.507943	1.470173
C	-3.234269	-0.683499	-0.625263
C	-1.512497	0.788376	-3.057854
C	-1.662815	2.563823	0.345700
C	-0.487882	2.851188	1.271577
C	-2.528796	3.793195	0.110379
C	-2.755995	-3.734769	-2.286110
C	-4.129305	-3.698528	-1.593542
C	-1.889052	-4.883552	-1.743705
C	-3.036923	-4.041955	-3.769936
O	-0.610964	-2.650759	0.887681
O	-2.734183	-1.659550	2.647474
O	-3.411264	-0.587345	0.787685
O	-3.014375	0.575788	-1.184155
O	-0.584753	-0.126625	-3.450287
O	-2.083326	1.503739	-3.822034
O	-0.472009	3.524938	2.256272
O	0.641885	2.203057	0.828934
O	-0.235286	-2.818245	-3.890518
O	-0.613182	-0.079067	1.249061
H	1.195306	1.552945	-1.027434
H	0.976164	-0.809334	-2.312107
H	0.005883	-3.008818	-1.899214
H	-2.556644	-1.703576	-2.919926
H	-2.386378	-3.306083	0.404359
H	-4.165799	-1.068218	-1.043368
H	-2.255808	1.785322	0.840450
H	-3.393307	3.526768	-0.502597
H	-1.964966	4.578301	-0.402990
H	-2.877648	4.191083	1.065920
H	-4.097453	-3.478465	-0.521389
H	-4.617787	-4.672040	-1.704070
H	-4.780763	-2.954693	-2.066569
H	-1.532787	-4.733451	-0.720203
H	-1.010270	-5.028906	-2.380385
H	-2.464317	-5.815019	-1.759095
H	-3.668070	-3.265553	-4.217666
H	-3.571423	-4.994429	-3.853527
H	-2.111157	-4.115142	-4.342648
H	-0.561295	-2.145111	-4.511231
H	0.990101	-0.639569	0.066027
H	-0.831299	2.774333	-1.637117
H	-0.010458	0.166002	1.968463

1B$^-$

C	-0.022536	-0.200355	-1.723361
C	-1.340529	0.482136	-1.351667
C	-1.925107	-0.440275	-0.243163
C	-0.664585	-0.991746	0.487164
C	0.572559	-0.609650	-0.350939
C	1.358450	-1.939134	-0.377041
C	2.423750	-1.797574	0.710952
C	2.817785	-0.310140	0.713394
C	1.416423	0.374759	0.528066
C	1.488174	1.892710	0.243662
C	0.418467	2.540693	1.111109
C	0.611277	0.372708	1.878115
C	-0.645391	-2.525233	0.585014
C	-2.931464	0.285501	0.654820
C	-3.906413	0.915161	-0.377382
C	-3.607489	-0.667589	1.634273
C	3.989747	0.024984	-0.275102
C	4.466256	1.488461	-0.175443
C	3.649947	-0.243660	-1.745590
C	5.200869	-0.841794	0.120159
O	-0.263925	-1.350707	-2.508609
O	-2.539446	-1.538548	-0.912912
O	1.502543	2.344843	-1.070694
O	0.019075	3.665430	1.058995
O	0.078431	1.675055	2.094909
O	-0.430902	-0.539627	1.815711
O	0.452059	-2.997588	-0.057383
O	-1.401162	-3.233079	1.180216
O	-3.678450	2.109819	-0.706377
O	-1.084294	1.782399	-0.877266
H	0.638091	0.452045	-2.295851
H	-2.028049	0.505819	-2.206500
H	1.747867	-2.166663	-1.367230
H	1.940517	-2.042342	1.666096
H	3.251145	-2.497398	0.587476
H	3.201435	-0.029230	1.705968
H	2.411406	2.241883	0.720620
H	1.214045	0.132067	2.760394
H	-2.401338	1.076917	1.190984
H	-4.282350	-0.107778	2.289349
H	-2.872052	-1.193222	2.250096
H	-4.199040	-1.407056	1.091479
H	3.798619	2.179875	-0.693845
H	5.448259	1.573134	-0.654852
H	4.584087	1.808239	0.867476
H	3.485488	-1.306159	-1.945649
H	4.487360	0.077689	-2.376420
H	2.767682	0.325387	-2.052956
H	5.457533	-0.704704	1.177896
H	6.071917	-0.549328	-0.476038
H	5.034935	-1.907599	-0.054566
H	0.554355	2.425976	-1.315781
H	-1.118971	-1.723774	-2.215716
H	-3.466803	-1.228515	-1.085895
H	-2.000872	2.182234	-0.723305
O	-4.728243	0.118473	-0.889408

C1

C	-0.014173	-0.198299	-1.624798
C	-1.311041	0.504165	-1.319174
C	-1.908769	-0.438582	-0.226886
C	-0.658547	-0.967912	0.542264
C	0.594089	-0.605160	-0.294792
C	1.366238	-1.941195	-0.303689
C	2.425996	-1.784845	0.787192
C	2.829553	-0.300804	0.747985
C	1.431515	0.386924	0.574409
C	1.507457	1.898346	0.255234
C	0.451310	2.570844	1.121595
C	0.620490	0.410280	1.919645
C	-0.645165	-2.499682	0.658392
C	-2.962394	0.254625	0.640235
C	-3.915787	0.863096	-0.422594
C	-3.642471	-0.718096	1.597137
C	3.962019	0.012943	-0.294132
C	4.478219	1.463865	-0.192884
C	3.547151	-0.221800	-1.754170
C	5.167711	-0.889899	0.029264
O	-0.109254	-1.223011	-2.530286

O	-2.456170	-1.538861	-0.946573
O	1.521609	2.317825	-1.066877
O	0.066193	3.699932	1.063714
O	0.110704	1.722122	2.122729
O	-0.443457	-0.482725	1.859160
O	0.448552	-2.987346	0.021493
O	-1.406450	-3.195851	1.260546
O	-3.708178	2.063739	-0.738708
O	-1.081014	1.802771	-0.832045
H	-1.984392	0.522783	-2.185262
H	1.763116	-2.183653	-1.286590
H	1.933802	-1.997475	1.745837
H	3.247918	-2.494837	0.689976
H	3.244804	-0.001387	1.722369
H	2.436702	2.251658	0.717823
H	1.213489	0.164316	2.807193
H	-2.466144	1.056274	1.194102
H	-4.372356	-0.182698	2.212455
H	-2.914518	-1.204307	2.252964
H	-4.173595	-1.489569	1.036254
H	3.815539	2.174901	-0.690263
H	5.449690	1.529222	-0.696099
H	4.629006	1.771951	0.849399
H	3.407746	-1.283203	-1.978018
H	4.343423	0.141092	-2.415261
H	2.632154	0.325598	-2.003730
H	5.476026	-0.782149	1.076814
H	6.017617	-0.606725	-0.600897
H	4.966289	-1.947209	-0.160236
H	0.578419	2.346179	-1.337589
H	-1.035024	-1.543001	-2.506852
H	-3.395589	-1.264627	-1.118707
H	-2.002877	2.187697	-0.697634
O	-4.694128	0.043646	-0.968129

C2

C	0.036795	-0.199670	-1.744266
C	-1.213762	0.543161	-1.310033
C	-1.883422	-0.369913	-0.279565
C	-0.658020	-1.039842	0.434112
C	0.600180	-0.663402	-0.373745
C	1.387162	-1.987274	-0.377780
C	2.454543	-1.829189	0.707970
C	2.821922	-0.330884	0.717779
C	1.411742	0.331536	0.520451
C	1.435854	1.857056	0.234663
C	0.348183	2.468944	1.110484
C	0.601324	0.298310	1.862861
C	-0.669596	-2.573512	0.490431
C	-2.859053	0.337214	0.671809
C	-3.947470	1.009440	-0.182147
C	-3.492545	-0.635298	1.662633
C	4.002649	0.014697	-0.254072
C	4.396994	1.503814	-0.237541
C	3.720449	-0.368064	-1.710988
C	5.245821	-0.756341	0.229679
O	-0.226048	-1.345559	-2.543491
O	-2.548223	-1.451252	-0.965990
O	1.423505	2.313125	-1.072173
O	-0.076843	3.584751	1.090865
O	0.024103	1.578669	2.084881
O	-0.413614	-0.644775	1.781407
O	0.478987	-3.045534	-0.051621
O	-1.486747	-3.287392	0.993456
O	-3.625746	2.151939	-0.764725
O	-1.124740	1.838648	-1.075909
H	0.728369	0.435215	-2.296058
H	1.778020	-2.223521	-1.366158
H	1.981655	-2.092737	1.662741
H	3.299059	-2.507369	0.572881
H	3.189453	-0.044248	1.714925
H	2.347715	2.233200	0.715774
H	1.199910	0.064399	2.749625
H	-2.298724	1.120269	1.193901
H	-4.204681	-0.107828	2.303482
H	-2.726507	-1.103544	2.283260
H	-4.031066	-1.417142	1.125356
H	3.687712	2.127046	-0.786297
H	5.372221	1.613589	-0.726400
H	4.502209	1.884099	0.786256
H	3.625488	-1.450215	-1.841542
H	4.552410	-0.036544	-2.343527
H	2.811641	0.119766	-2.075696
H	5.487230	-0.500867	1.268781
H	6.108738	-0.487352	-0.389466
H	5.127201	-1.840866	0.165512
H	0.450097	2.326045	-1.312293
H	-1.125322	-1.637736	-2.309359
H	-3.325914	-1.056832	-1.397490
H	-2.609485	2.236099	-0.798364
O	-5.042622	0.501784	-0.325326

C3

C	0.032742	-0.243905	-1.700794
C	-1.290352	0.456419	-1.368615
C	-1.912079	-0.441507	-0.260331
C	-0.673401	-1.007587	0.491694
C	0.598054	-0.624423	-0.305179
C	1.373213	-1.917465	-0.197221
C	2.493061	-1.777676	0.792949
C	2.837748	-0.279625	0.752566
C	1.417260	0.372060	0.578593
C	1.474334	1.893330	0.309649
C	0.381232	2.513657	1.168205
C	0.594582	0.338755	1.917447
C	-0.660267	-2.546918	0.557334
C	-2.918807	0.308163	0.617522
C	-3.876672	0.937596	-0.431511
C	-3.617041	-0.629022	1.597082
C	3.961097	0.043924	-0.294368
C	4.459634	1.500194	-0.209243
C	3.526108	-0.220444	-1.740815
C	5.180691	-0.843056	0.029339
O	-0.201442	-1.412136	-2.457764
O	-2.538056	-1.533112	-0.929769
O	1.504825	2.360084	-0.997995
O	-0.043200	3.628969	1.122575
O	0.041846	1.630170	2.139498
O	-0.440602	-0.588079	1.831482
O	0.529566	-2.991381	0.028564
O	-1.450401	-3.285280	1.054388
O	-3.622985	2.121492	-0.781989
O	-1.046353	1.762864	-0.907307
H	0.707977	0.393538	-2.271826
H	-1.955878	0.468662	-2.241251
H	2.089823	-2.044227	1.784434
H	3.330695	-2.452808	0.611212
H	3.241464	0.040769	1.724774
H	2.389740	2.245071	0.803292
H	1.184242	0.090972	2.806301
H	-2.386086	1.098389	1.153038
H	-4.291559	-0.056492	2.241211
H	-2.894302	-1.157421	2.225885
H	-4.211809	-1.365420	1.053913
H	3.758578	2.208891	-0.653462
H	5.397446	1.587992	-0.769643
H	4.669787	1.795255	0.826936
H	3.121894	-1.229378	-1.878836
H	4.389020	-0.107191	-2.408512
H	2.769667	0.506724	-2.046322
H	5.445007	-0.788372	1.092954
H	6.045509	-0.500492	-0.548309
H	5.020131	-1.892465	-0.229896
H	0.559967	2.408681	-1.267823
H	-1.082401	-1.750644	-2.205187
H	-3.458799	-1.211759	-1.113693
H	-1.968460	2.166672	-0.778789
O	-4.712713	0.149584	-0.931709

C7

C	-0.031411	-0.233408	-1.726051
C	-1.347356	0.455513	-1.359571
C	-1.931475	-0.455967	-0.241087
C	-0.670912	-0.990205	0.500362

C	0.564908	-0.626101	-0.349909
C	1.347339	-1.964398	-0.362969
C	2.450347	-1.755470	0.612084
C	2.810876	-0.308790	0.714926
C	1.410537	0.374149	0.516482
C	1.488026	1.889341	0.210979
C	0.430593	2.552159	1.082765
C	0.613184	0.390911	1.869312
C	-0.646914	-2.517953	0.655324
C	-2.941484	0.280432	0.644439
C	-3.912813	0.895696	-0.399136
C	-3.620049	-0.659010	1.635100
C	4.001120	0.052488	-0.260675
C	4.536801	1.484194	-0.073431
C	3.625204	-0.121511	-1.735579
C	5.156010	-0.906072	0.077266
O	-0.281406	-1.394373	-2.493332
O	-2.541986	-1.565078	-0.896036
O	1.493381	2.335188	-1.105337
O	0.031059	3.676112	1.015757
O	0.104417	1.703841	2.085195
O	-0.447361	-0.498538	1.814592
O	0.462113	-3.012798	0.052981
O	-1.410829	-3.205364	1.263773
O	-3.686027	2.087052	-0.740881
O	-1.089677	1.759552	-0.895938
H	0.632082	0.407214	-2.308778
H	-2.035637	0.472659	-2.213951
H	1.664792	-2.238063	-1.373854
H	2.977254	-2.574248	1.085009
H	3.185823	-0.062693	1.720992
H	2.417516	2.236608	0.676483
H	1.216575	0.141615	2.747797
H	-2.413153	1.078906	1.171753
H	-4.300290	-0.090597	2.277094
H	-2.886341	-1.172278	2.263092
H	-4.206284	-1.409265	1.101333
H	3.916001	2.228449	-0.578005
H	5.537216	1.550480	-0.517032
H	4.630566	1.749984	0.986894
H	3.354780	-1.154952	-1.971935
H	4.485046	0.141878	-2.363456
H	2.799273	0.541996	-2.006967
H	5.444075	-0.824159	1.132991
H	6.033802	-0.660894	-0.530518
H	4.890667	-1.947752	-0.124676
H	0.543492	2.408592	-1.346498
H	-1.140251	-1.755256	-2.194714
H	-3.469588	-1.258576	-1.075199
H	-2.006295	2.161675	-0.749095
O	-4.729996	0.090945	-0.906223

C8

C	-0.141028	-0.160577	-1.782437
C	-1.437145	0.521651	-1.337671
C	-1.976666	-0.421353	-0.220337
C	-0.689311	-1.003068	0.438110
C	0.501774	-0.608235	-0.448061
C	1.281674	-1.937855	-0.548715
C	2.409113	-1.826679	0.486975
C	2.754313	-0.359287	0.454293
C	1.403739	0.357633	0.408992
C	1.462014	1.879987	0.157375
C	0.429643	2.504694	1.083697
C	0.663406	0.321480	1.801824
C	-0.676438	-2.540055	0.486509
C	-2.933235	0.292269	0.738991
C	-3.953734	0.947445	-0.231909
C	-3.566595	-0.676963	1.731139
C	4.061792	0.029284	-0.236401
C	4.425152	1.523743	-0.255136
C	4.000737	-0.438261	-1.707025
C	5.223746	-0.689576	0.478001
O	-0.426736	-1.288270	-2.586252
O	-2.630514	-1.499909	-0.884620
O	1.431379	2.357547	-1.146897
O	0.026884	3.629304	1.076245
O	0.138290	1.617389	2.065208
O	-0.378848	-0.593898	1.766130
O	0.382640	-2.998858	-0.231274
O	-1.403083	-3.263389	1.098124
O	-3.735187	2.147071	-0.548061
O	-1.153277	1.811381	-0.852976
H	0.512035	0.496128	-2.360425
H	-2.164002	0.563100	-2.158624
H	1.647106	-2.120601	-1.560146
H	1.990665	-2.111659	1.464398
H	3.235477	-2.507437	0.276063
H	2.399446	2.217637	0.609285
H	1.312447	0.063609	2.643980
H	-2.373055	1.070601	1.263913
H	-4.203593	-0.127063	2.430958
H	-2.805222	-1.220167	2.298524
H	-4.190119	-1.400681	1.203096
H	3.750124	2.111203	-0.882056
H	5.432632	1.624325	-0.675065
H	4.450680	1.948772	0.754543
H	3.869638	-1.522833	-1.782929
H	4.931309	-0.176910	-2.225371
H	3.170392	0.053409	-2.226812
H	5.292899	-0.362015	1.521021
H	6.169888	-0.448470	-0.019555
H	5.116103	-1.777043	0.471377
H	0.476128	2.452139	-1.355039
H	-1.269590	-1.664001	-2.263308
H	-3.563285	-1.180848	-1.005154
H	-2.058538	2.212356	-0.646570
O	-4.804359	0.164962	-0.718464

C8tBu

C	0.035619	-0.222630	-1.694920
C	-1.294927	0.454095	-1.358782
C	-1.895915	-0.458834	-0.251371
C	-0.646242	-0.988768	0.512945
C	0.602409	-0.608781	-0.306686
C	1.403693	-1.931421	-0.289165
C	2.419142	-1.754564	0.839518
C	2.814123	-0.273607	0.771801
C	1.416780	0.399935	0.572178
C	1.493454	1.915637	0.271861
C	0.408019	2.568927	1.116780
C	0.591541	0.409088	1.907576
C	-0.615494	-2.521268	0.631570
C	-2.927480	0.268899	0.615921
C	-3.883849	0.879297	-0.444510
C	-3.617010	-0.678935	1.590967
C	3.929929	0.021247	-0.308283
C	4.515552	1.444910	-0.155284
C	3.444232	-0.121452	-1.720402
C	5.094665	-0.960451	-0.073814
O	-0.177102	-1.383011	-2.473632
O	-2.485945	-1.570970	-0.919882
O	1.523647	2.363354	-1.043819
O	0.006059	3.691689	1.045165
O	0.048870	1.711246	2.100243
O	-0.444559	-0.510696	1.838402
O	0.495151	-2.993470	0.010632
O	-1.375713	-3.227872	1.222660
O	-3.657463	2.072001	-0.781797
O	-1.056505	1.761793	-0.892562
H	0.707298	0.427362	-2.255795
H	-1.965346	0.464772	-2.227282
H	1.845750	-2.161601	-1.257359
H	1.890122	-1.940490	1.783906
H	3.245949	-2.463980	0.797216
H	3.239288	0.055358	1.732172
H	2.413165	2.266872	0.757060
H	1.181402	0.182554	2.802514
H	-2.415571	1.070203	1.155125
H	-4.312508	-0.118225	2.223310
H	-2.890767	-1.189470	2.230017
H	-4.188232	-1.431081	1.043823
H	3.859527	2.209622	-0.575228
H	5.461122	1.501626	-0.704830

H	4.724362	1.682911	0.895484
H	3.655731	-1.011775	-2.301865
H	2.919104	0.700147	-2.190650
H	5.405949	-0.963670	0.977931
H	5.953989	-0.662575	-0.682679
H	4.835412	-1.983960	-0.357042
H	0.578342	2.421999	-1.307246
H	-1.032148	-1.764071	-2.192070
H	-3.411313	-1.270729	-1.118089
H	-1.978647	2.156758	-0.762872
O	-4.688236	0.070639	-0.965437

C10

C	0.024773	-0.247606	-1.679845
C	-1.322817	0.428794	-1.402325
C	-1.936878	-0.445065	-0.270216
C	-0.710231	-0.840467	0.594671
C	0.557018	-0.651831	-0.271425
C	1.259840	-2.020486	-0.222528
C	2.494859	-1.851456	0.673735
C	2.863512	-0.350509	0.678016
C	1.450125	0.309880	0.551915
C	1.328514	1.765680	0.255728
C	0.956181	2.515254	1.443062
C	0.751618	0.315901	1.952690
C	-0.688190	-2.325940	0.992162
C	-3.059137	0.286084	0.473888
C	-3.974255	0.755125	-0.687765
C	-3.760438	-0.605607	1.491273
C	3.966754	0.068946	-0.331470
C	4.394946	1.523617	-0.074792
C	3.521393	-0.073228	-1.790020
C	5.208530	-0.809760	-0.099108
O	-0.171214	-1.391632	-2.485924
O	-2.418742	-1.639052	-0.881045
O	1.373484	2.330690	-0.957703
O	0.820150	3.702447	1.588623
O	0.819538	1.615157	2.481826
O	-0.591827	-0.018702	1.739531
O	0.335824	-2.953915	0.357616
O	-1.400881	-2.889306	1.769684
O	-3.798587	1.934531	-1.095704
O	-1.167385	1.777775	-1.009309
H	0.710269	0.407311	-2.216645
H	-1.961195	0.364723	-2.292214
H	1.487553	-2.392144	-1.220875
H	2.206584	-2.161382	1.684938
H	3.315931	-2.504796	0.373544
H	3.263356	-0.080709	1.666896
H	1.213385	-0.354248	2.687763
H	-2.621783	1.157004	0.965378
H	-4.552766	-0.041173	1.992867
H	-3.057558	-0.975597	2.242946
H	-4.219458	-1.462686	0.994198
H	3.610297	2.242987	-0.315317
H	5.260834	1.766887	-0.701682
H	4.689988	1.666026	0.971852
H	3.130668	-1.075133	-2.004848
H	4.374058	0.097611	-2.458026
H	2.753414	0.664330	-2.034992
H	5.485309	-0.835828	0.961970
H	6.057932	-0.402209	-0.658006
H	5.061627	-1.838958	-0.438979
H	0.424250	2.505765	-1.198382
H	-0.996868	-1.811872	-2.171528
H	-3.349481	-1.411971	-1.147240
H	-2.114391	2.122979	-0.973260
O	-4.688695	-0.140189	-1.198765

C12

C	-0.028042	-0.205354	-1.730653
C	-1.343848	0.474304	-1.345469
C	-1.927169	-0.459533	-0.247053
C	-0.662232	-0.995206	0.487107
C	0.571200	-0.614500	-0.363486
C	1.360947	-1.940253	-0.368533
C	2.409161	-1.778679	0.733298
C	2.807593	-0.293811	0.716455
C	1.406188	0.393732	0.504646
C	1.505184	1.916981	0.218289
C	0.491401	2.588752	1.136648
C	0.572568	0.422369	1.794854
C	-0.629671	-2.521927	0.619633
C	-2.942654	0.256516	0.649699
C	-3.910929	0.888370	-0.386625
C	-3.620227	-0.703032	1.621208
C	3.982770	0.026713	-0.274986
C	4.495105	1.478237	-0.162083
C	3.631428	-0.211661	-1.748457
C	5.175791	-0.873136	0.101472
O	-0.267281	-1.349292	-2.524369
O	-2.528481	-1.562394	-0.918164
O	1.485743	2.381352	-1.090271
O	0.127456	3.724316	1.130555
O	0.144837	1.701089	2.117955
O	-0.450272	-0.469507	1.803296
O	0.452653	-2.998341	-0.042110
O	-1.374366	-3.221187	1.239027
O	-3.683197	2.086196	-0.705307
O	-1.085443	1.766690	-0.843087
H	0.629860	0.453266	-2.299686
H	-2.033701	0.516434	-2.197291
H	1.759824	-2.183193	-1.351142
H	1.908682	-1.993273	1.686300
H	3.234452	-2.485373	0.641353
H	3.173530	0.000048	1.710542
H	2.452298	2.237900	0.662930
H	-2.419795	1.047750	1.192821
H	-4.301666	-0.148369	2.273687
H	-2.886574	-1.227498	2.240198
H	-4.205020	-1.443717	1.072695
H	3.852816	2.188428	-0.687936
H	5.484684	1.541795	-0.628847
H	4.606111	1.792599	0.883072
H	3.435940	-1.265296	-1.965326
H	4.476532	0.094732	-2.376724
H	2.765818	0.387647	-2.045797
H	5.428698	-0.772382	1.164128
H	6.055566	-0.580971	-0.481899
H	4.989638	-1.929700	-0.105775
H	0.532494	2.431793	-1.322361
H	-1.113490	-1.735579	-2.223540
H	-3.457106	-1.258819	-1.096402
H	-2.005018	2.164312	-0.699564
O	-4.723686	0.091527	-0.911866

C14

C	0.003787	-0.256233	-1.787950
C	-1.324238	0.420893	-1.446724
C	-1.921835	-0.491226	-0.336305
C	-0.673353	-1.065978	0.399211
C	0.575674	-0.653087	-0.403346
C	1.390400	-1.965470	-0.412921
C	2.421421	-1.808991	0.705198
C	2.781698	-0.312941	0.728630
C	1.372698	0.343712	0.507397
C	1.416776	1.864366	0.239846
C	0.289610	2.474029	1.064275
C	0.525851	0.311778	1.830643
C	-0.639759	-2.601751	0.461834
C	-2.925370	0.203198	0.520737
C	-3.929502	0.929438	-0.361174
C	-3.380224	-0.446524	1.791993
C	3.972321	0.057903	-0.223857
C	4.419768	1.527217	-0.083482
C	3.673096	-0.187148	-1.707221
C	5.187560	-0.796733	0.184762
O	-0.213734	-1.417981	-2.566750
O	-2.549406	-1.602625	-1.017557
O	1.475177	2.324785	-1.070320
O	-0.122294	3.594263	1.016892
O	-0.086450	1.582222	2.011251
O	-0.456465	-0.662132	1.745366
O	0.497253	-3.045283	-0.125666

O	-1.415170	-3.332911	1.002846
O	-3.594613	2.057001	-0.828316
O	-1.086812	1.719258	-0.976797
H	0.669438	0.397018	-2.354019
H	-1.997708	0.429720	-2.313929
H	1.812865	-2.179860	-1.392681
H	1.917128	-2.070879	1.644405
H	3.267601	-2.488964	0.599566
H	3.131101	-0.032437	1.733796
H	2.309526	2.233349	0.759072
H	1.111311	0.109887	2.734244
H	-3.003883	0.097338	2.667450
H	-3.019970	-1.478976	1.869807
H	-4.473943	-0.451173	1.822031
H	3.752752	2.216536	-0.605241
H	5.412094	1.639193	-0.535313
H	4.504294	1.827849	0.968328
H	3.521334	-1.247037	-1.930251
H	4.524648	0.150694	-2.316
H	2.795601	0.382701	-2.026162
H	5.410027	-0.684272	1.253137
H	6.070254	-0.472313	-0.376742
H	5.047174	-1.860203	-0.023759
H	0.533294	2.378347	-1.350998
H	-1.084557	-1.780318	-2.314225
H	-3.490664	-1.345416	-1.111779
H	-2.033751	2.094541	-0.815495
O	-4.947351	0.258643	-0.627262

C14Me

C	-0.020036	-0.205441	-1.720801
C	-1.340610	0.479283	-1.359245
C	-1.921672	-0.435143	-0.245095
C	-0.666319	-0.977537	0.495400
C	0.571906	-0.603612	-0.343056
C	1.356730	-1.934057	-0.354315
C	2.419259	-1.781852	0.735257
C	2.813879	-0.294530	0.725117
C	1.413400	0.389147	0.528545
C	1.485859	1.904082	0.228502
C	0.414352	2.561408	1.086470
C	0.604105	0.401146	1.876320
C	-0.650056	-2.509565	0.610396
C	-2.929284	0.296287	0.670188
C	-3.922850	0.897354	-0.365788
C	-3.556401	-0.636014	1.641723
C	3.989554	0.029963	-0.262474
C	4.463897	1.495175	-0.180314
C	3.656355	-0.259119	-1.730575
C	5.200265	-0.829563	0.149579
O	-0.259724	-1.362137	-2.497195
O	-2.537467	-1.536461	-0.901578
O	1.502476	2.342705	-1.090577
O	0.015609	3.685733	1.021168
O	0.071588	1.706512	2.078468
O	-0.439134	-0.510242	1.820061
O	0.449145	-2.988772	-0.026636
O	-1.407388	-3.210050	1.211045
O	-3.694890	2.080202	-0.729881
O	-1.089468	1.784820	-0.895816
H	0.642301	0.441813	-2.297191
H	-2.026116	0.493018	-2.215621
H	1.748846	-2.171237	-1.341189
H	1.933372	-2.017868	1.691262
H	3.246833	-2.482978	0.620507
H	3.194269	-0.004125	1.716222
H	2.408124	2.258400	0.703430
H	1.204262	0.169372	2.762733
H	-2.387514	1.096108	1.183259
H	-3.029966	-0.910470	2.547342
H	-4.403880	-1.227999	1.322213
H	3.797243	2.179192	-0.709705
H	5.447410	1.574705	-0.657542
H	4.578135	1.828498	0.858749
H	3.496050	-1.324764	-1.916890
H	4.495112	0.056521	-2.362475
H	2.773305	0.302604	-2.049137
H	5.453715	-0.675640	1.205764
H	6.072815	-0.545588	-0.448547
H	5.035846	-1.898134	-0.008843
H	0.555266	2.425665	-1.337686
H	-1.115669	-1.733106	-2.204512
H	-3.477134	-1.249819	-1.040902
H	-2.006879	2.180005	-0.747059
O	-4.751475	0.085371	-0.838775

C1OH

C	0.003464	-0.217510	-1.721871
C	-1.349899	0.462996	-1.372439
C	-1.924977	-0.453424	-0.254622
C	-0.664763	-1.008423	0.475317
C	0.581368	-0.627176	-0.346654
C	1.366843	-1.957231	-0.366627
C	2.436687	-1.805701	0.716631
C	2.821206	-0.315553	0.718743
C	1.415976	0.359610	0.533891
C	1.471113	1.877235	0.246300
C	0.393969	2.514660	1.112810
C	0.606225	0.348300	1.881247
C	-0.649894	-2.543029	0.567658
C	-2.908009	0.303854	0.649508
C	-3.893424	0.951714	-0.361534
C	-3.590293	-0.631329	1.641421
C	3.988696	0.028247	-0.271584
C	4.453923	1.495354	-0.173051
C	3.647236	-0.242498	-1.741314
C	5.206991	-0.829846	0.120099
O	-0.216471	-1.289397	-2.532215
O	-2.545145	-1.543215	-0.900569
O	1.475585	2.325435	-1.070624
O	-0.018446	3.634202	1.056615
O	0.066743	1.648015	2.099877
O	-0.429530	-0.569296	1.810028
O	0.467933	-3.014773	-0.041188
O	-1.413712	-3.250132	1.151563
O	-3.602594	2.123913	-0.748293
O	-1.098774	1.766366	-0.924045
H	0.619174	0.507308	-2.276111
H	-2.021406	0.453111	-2.238269
H	1.760496	-2.188908	-1.355651
H	1.957579	-2.054786	1.672466
H	3.268365	-2.499856	0.589969
H	3.203969	-0.032203	1.710873
H	2.390516	2.238794	0.721490
H	1.206630	0.108181	2.765103
H	-2.356071	1.087658	1.174745
H	-4.242434	-0.054915	2.305043
H	-2.857991	-1.172892	2.246827
H	-4.207102	-1.356598	1.108219
H	3.780028	2.181767	-0.690119
H	5.434423	1.587948	-0.654014
H	4.570659	1.815984	0.869645
H	3.484911	-1.305652	-1.940222
H	4.482359	0.080318	-2.374300
H	2.764036	0.326264	-2.047257
H	5.465203	-0.690723	1.177109
H	6.074429	-0.531186	-0.478255
H	5.048139	-1.896846	-0.053864
H	0.522883	2.421366	-1.300926
H	-3.465211	-1.242430	-1.080362
H	-2.047231	2.155485	-0.775520
O	-4.794268	0.209144	-0.798532

C2OH

C	-0.001618	-0.268795	-1.726985
C	-1.324555	0.441912	-1.366399
C	-1.912637	-0.478881	-0.253944
C	-0.660498	-1.051845	0.469894
C	0.582660	-0.658494	-0.351264
C	1.390842	-1.975001	-0.354472
C	2.454787	-1.799282	0.729549
C	2.814171	-0.302219	0.716595
C	1.397061	0.346724	0.529103
C	1.424912	1.866719	0.242756

C	0.354185	2.489451	1.129148
C	0.592567	0.317732	1.878481
C	-0.633691	-2.585419	0.558404
C	-2.852980	0.310892	0.654333
C	-3.762965	1.038793	-0.345861
C	-3.614473	-0.590113	1.618054
C	3.974554	0.055311	-0.277507
C	4.425343	1.526474	-0.170880
C	3.630657	-0.204512	-1.748335
C	5.201257	-0.795627	0.103286
O	-0.260951	-1.438735	-2.481828
O	-2.589207	-1.569234	-0.884824
O	1.407772	2.314730	-1.071914
O	-0.046019	3.612919	1.114401
O	0.038257	1.602923	2.110862
O	-0.431567	-0.620109	1.806327
O	0.501755	-3.045122	-0.020234
O	-1.415511	-3.302231	1.109014
O	-3.283691	2.109353	-0.859073
O	-1.112208	1.701974	-0.883107
H	0.662341	0.361676	-2.318356
H	-1.989366	0.433577	-2.247703
H	1.785163	-2.211971	-1.340884
H	1.978165	-2.045018	1.687540
H	3.298289	-2.480751	0.612074
H	3.193979	-0.004987	1.705920
H	2.345750	2.239819	0.706314
H	1.199826	0.073964	2.757086
H	-2.256694	1.053592	1.185321
H	-4.220651	0.016041	2.297592
H	-2.920105	-1.198576	2.204508
H	-4.287114	-1.255719	1.074210
H	3.742001	2.207267	-0.683201
H	5.403770	1.632589	-0.653495
H	4.539549	1.843390	0.873244
H	3.457060	-1.264313	-1.955006
H	4.469958	0.113841	-2.378429
H	2.753186	0.374837	-2.049006
H	5.454543	-0.674443	1.163847
H	6.067392	-0.476413	-0.486327
H	5.054343	-1.860782	-0.092164
H	0.446823	2.363408	-1.304442
H	-1.133744	-1.777136	-2.205140
H	-3.483562	-1.235311	-1.098280
O	-4.827096	0.502828	-0.688045

C3OH

C	0.040542	-0.234348	-1.778540
C	-1.204394	0.619321	-1.530732
C	-1.993007	-0.575944	-0.248190
C	-0.658229	-1.016083	0.443612
C	0.581956	-0.624130	-0.385087
C	1.382294	-1.948985	-0.395637
C	2.432325	-1.800856	0.705375
C	2.818179	-0.312165	0.713843
C	1.416994	0.366728	0.501153
C	1.494164	1.882834	0.213263
C	0.398056	2.530758	1.047251
C	0.593641	0.369596	1.836800
C	-0.628752	-2.552108	0.529033
C	-2.929757	0.257370	0.639929
C	-4.027221	0.958401	-0.169358
C	-3.566762	-0.673946	1.672569
C	4.014051	0.027335	-0.244664
C	4.485346	1.491428	-0.130883
C	3.713883	-0.239106	-1.723617
C	5.216159	-0.838146	0.180702
O	-0.263886	-1.382087	-2.531493
O	-2.501129	-1.473172	-1.009087
O	1.539412	2.325321	-1.102261
O	0.001955	3.656297	0.992412
O	0.027978	1.662823	2.021735
O	-0.426486	-0.566780	1.774353
O	0.484819	-3.016678	-0.086273
O	-1.404660	-3.266132	1.091376
O	-3.647641	2.014915	-0.895307
O	-1.033226	1.773964	-0.968500
H	0.774033	0.341031	-2.351206
H	-1.970643	0.535691	-2.316831
H	1.786948	-2.172169	-1.381293
H	1.937434	-2.048389	1.653711
H	3.264429	-2.496898	0.592481
H	3.178276	-0.028772	1.714512
H	2.403985	2.236016	0.712844
H	1.187374	0.155321	2.732096
H	-2.324935	1.028466	1.131997
H	-4.228937	-0.110314	2.335352
H	-2.797420	-1.173862	2.265240
H	-4.159165	-1.429573	1.153304
H	3.828017	2.182305	-0.662935
H	5.478140	1.579161	-0.587071
H	4.578001	1.809033	0.915271
H	3.533262	-1.298503	-1.926576
H	4.576296	0.063680	-2.329658
H	2.853390	0.348241	-2.056385
H	5.440194	-0.708595	1.246798
H	6.103760	-0.537967	-0.386549
H	5.059727	-1.903146	-0.007212
H	0.587845	2.371143	-1.359100
H	-1.112685	-1.728570	-2.172758
H	-2.660673	2.143456	-0.844374
O	-5.185316	0.619234	-0.132085

C10OH

C	-0.046100	-0.148185	-1.719791
C	-1.360444	0.517589	-1.310273
C	-1.933086	-0.441179	-0.230045
C	-0.663655	-0.973649	0.494151
C	0.562292	-0.591547	-0.363186
C	1.339927	-1.922735	-0.406827
C	2.401310	-1.796899	0.688684
C	2.812255	-0.313744	0.706409
C	1.418590	0.385388	0.505644
C	1.489343	1.887447	0.172677
C	0.436368	2.596256	1.041008
C	0.605802	0.426668	1.847430
C	-0.637456	-2.505542	0.599179
C	-2.960434	0.242803	0.676154
C	-3.941705	0.869601	-0.352116
C	-3.620074	-0.741495	1.635576
C	4.001390	0.006564	-0.266580
C	4.493084	1.466362	-0.172624
C	3.679748	-0.281800	-1.737826
C	5.202412	-0.861289	0.157837
O	-0.273118	-1.274987	-2.540721
O	-2.515264	-1.543060	-0.921510
O	1.549455	2.315346	-1.108077
O	0.116124	3.742586	1.000693
O	0.064699	1.737879	2.014417
O	-0.421640	-0.497337	1.814494
O	0.429183	-2.979150	-0.095122
O	-1.365727	-3.213551	1.227290
O	-3.774049	2.083748	-0.619153
O	-1.078697	1.788430	-0.786284
H	0.595785	0.544004	-2.267538
H	-2.056303	0.577131	-2.156775
H	1.729124	-2.142373	-1.398960
H	1.909905	-2.042517	1.639409
H	3.222011	-2.504692	0.566772
H	3.187316	-0.043785	1.705257
H	2.412153	2.281975	0.640528
H	1.206292	0.228421	2.741840
H	-2.452709	1.037382	1.228409
H	-4.322133	-0.210543	2.286417
H	-2.878223	-1.251410	2.257530
H	-4.179172	-1.494025	1.076202
H	3.875391	2.157826	-0.749300
H	5.504044	1.529263	-0.589993
H	4.548050	1.815318	0.866545
H	3.542509	-1.350432	-1.925218
H	4.512827	0.053049	-2.367168
H	2.782455	0.255609	-2.060857
H	5.459512	-0.692046	1.210701
H	6.077057	-0.598527	-0.446785

H	5.024817	-1.929852	0.018347
H	-1.118121	-1.670413	-2.246540
H	-3.448413	-1.238707	-1.098725
H	-1.972500	2.201315	-0.629192
O	-4.705420	0.045464	-0.919182

1B radical (SET)

C	-0.062093	-0.170339	-1.661434
C	-1.392762	0.471085	-1.232412
C	-1.911920	-0.458109	-0.100184
C	-0.596589	-1.002656	0.568126
C	0.589458	-0.597946	-0.319163
C	1.375930	-1.928345	-0.397555
C	2.454342	-1.815033	0.678143
C	2.862559	-0.331459	0.699642
C	1.466349	0.374137	0.547778
C	1.542238	1.892047	0.267495
C	0.443315	2.527720	1.101556
C	0.685141	0.382934	1.911540
C	-0.577222	-2.538199	0.639285
C	-2.820615	0.165128	0.902888
C	-4.521825	1.238909	-1.213160
C	-3.664083	-0.725450	1.751208
C	4.021401	0.010283	-0.300782
C	4.505601	1.469834	-0.183136
C	3.659252	-0.244750	-1.769033
C	5.232601	-0.867253	0.069560
O	-0.278727	-1.274667	-2.506243
O	-2.490374	-1.590323	-0.750095
O	1.575277	2.340587	-1.050257
O	-0.022223	3.623562	0.986759
O	0.098119	1.672517	2.087799
O	-0.333944	-0.561612	1.885954
O	0.462501	-2.992064	-0.094043
O	-1.311761	-3.237726	1.268379
O	-4.097618	2.307484	-1.037465
O	-1.096453	1.773950	-0.791576
H	0.551270	0.536969	-2.218942
H	-2.097905	0.482185	-2.076794
H	1.748665	-2.130934	-1.398651
H	1.988047	-2.078183	1.637002
H	3.274387	-2.515946	0.524373
H	3.261717	-0.072809	1.691040
H	2.458010	2.240261	0.760477
H	1.300289	0.187177	2.793737
H	-2.492594	1.114208	1.317934
H	-4.331662	-0.148457	2.394386
H	-3.021004	-1.350447	2.389530
H	-4.258779	-1.418423	1.148161
H	3.843140	2.176804	-0.686781
H	5.483628	1.556268	-0.668004
H	4.636499	1.771725	0.863489
H	3.517803	-1.308537	-1.981059
H	4.477504	0.101643	-2.410026
H	2.760757	0.308396	-2.060615
H	5.510064	-0.736923	1.122372
H	6.093725	-0.576560	-0.540328
H	5.059843	-1.931199	-0.109042
H	0.660349	2.582759	-1.279771
H	-0.987783	-1.820572	-2.122318
H	-3.364093	-1.335624	-1.089762
H	-1.903615	2.243113	-0.524306
O	-4.942994	0.172790	-1.418220

2

O	-2.770398	2.342411	8.238563
O	-1.815365	2.377401	10.266783
O	-3.098140	5.575500	12.241438
O	-0.763349	6.650293	7.171026
O	-0.703296	7.395675	9.302163
O	-1.523430	6.944077	11.407447
O	0.090103	4.129977	8.221862
O	0.297694	5.223170	12.616188
C	-2.498961	4.458404	9.422774
C	-2.387469	2.962606	9.184001
C	-2.155359	5.741658	11.530256
C	-1.526466	4.734556	10.578398
C	-1.313077	3.330474	11.198145
C	0.201759	3.175329	11.341577
C	0.751062	4.607700	11.406687
C	-0.118674	5.336630	10.336169
C	0.331618	5.334280	8.886665
C	-0.442106	6.512831	8.316181
C	-0.396945	6.839099	10.590578
C	2.304107	4.696693	11.317274
C	2.765486	6.153467	11.158956
C	2.878283	3.807639	10.198326
C	2.903591	4.190380	12.643705
H	-0.122498	4.339128	7.294241
H	0.552272	4.663418	13.364481
H	-2.334229	5.001721	8.493762
H	-3.524257	4.658432	9.754851
H	-1.866219	3.220683	12.134480
H	0.560502	2.670106	10.440066
H	0.491554	2.576080	12.210424
H	1.391957	5.616133	8.816666
H	0.401936	7.424768	11.039521
H	3.856249	6.203073	11.238213
H	2.496387	6.594704	10.193967
H	2.344332	6.775466	11.955074
H	3.928931	4.067466	10.033242
H	2.846034	2.752269	10.487450
H	2.354173	3.890307	9.243173
H	3.992798	4.139129	12.550811
H	2.681529	4.866208	13.475573
H	2.554696	3.183135	12.896925

C1

O	-3.108848	2.341353	8.439658
O	-1.891075	2.376768	10.339642
O	-3.126478	5.576561	12.181521
O	-0.823434	6.603948	7.177257
O	-0.718139	7.390904	9.292378
O	-1.527719	6.946006	11.394841
O	0.157153	4.147063	8.209072
O	0.289561	5.185169	12.613818
C	-2.370659	4.416860	9.395519
C	-2.533217	2.969866	9.286208
C	-2.174284	5.749655	11.488181
C	-1.507486	4.733776	10.558037
C	-1.298913	3.337893	11.204196
C	0.217242	3.168185	11.281383
C	0.763929	4.601664	11.396883
C	-0.092562	5.351736	10.335530
C	0.355994	5.352560	8.882872
C	-0.462733	6.497671	8.313409
C	-0.396170	6.849852	10.584098
C	2.316797	4.700500	11.328995
C	2.768617	6.162563	11.198445
C	2.908260	3.834365	10.202191
C	2.902083	4.175386	12.654316
H	-0.145855	4.363854	7.308433
H	0.543384	4.613249	13.353283
H	-2.844775	5.113986	8.718045
H	-1.799073	3.279456	12.174073
H	0.544837	2.711607	10.342451
H	0.535994	2.527141	12.109251
H	1.406521	5.670472	8.815180
H	0.391712	7.453144	11.029604
H	3.857132	6.221402	11.299201
H	2.513779	6.611587	10.233008
H	2.325993	6.771243	11.993406
H	3.966119	4.084997	10.072172
H	2.854304	2.772043	10.461174
H	2.411431	3.951684	9.236220
H	3.992410	4.128566	12.573373
H	2.668182	4.837668	13.493753
H	2.553263	3.163291	12.888001

C6

O	-2.998943	2.318545	8.339513
O	-1.797550	2.383598	10.238468
O	-3.093058	5.530719	12.234419
O	-0.751447	6.669023	7.172402

O	-0.710945	7.396829	9.310016
O	-1.528498	6.918097	11.411903
O	0.092280	4.142676	8.214686
O	0.314318	5.241476	12.609017
C	-2.512260	4.469373	9.397596
C	-2.492704	2.962582	9.203326
C	-2.150041	5.706114	11.529397
C	-1.516090	4.721481	10.545897
C	-1.238698	3.330165	11.060635
C	0.217165	3.162399	11.377961
C	0.760129	4.607755	11.408920
C	-0.106124	5.339915	10.328340
C	0.344234	5.345901	8.881724
C	-0.435053	6.525571	8.318472
C	-0.400519	6.834855	10.596525
C	2.312988	4.696331	11.314747
C	2.767109	6.157015	11.170710
C	2.890381	3.823046	10.183983
C	2.912384	4.176904	12.635314
H	-0.058666	4.354851	7.276157
H	0.491686	4.656244	13.359607
H	-2.307259	4.963440	8.448905
H	-3.524487	4.745134	9.711645
H	0.676135	2.579855	10.570350
H	0.412660	2.639136	12.321294
H	1.405248	5.623926	8.807203
H	0.391253	7.427022	11.050062
H	3.857539	6.211329	11.251119
H	2.496299	6.605174	10.209179
H	2.341588	6.769158	11.971952
H	3.926656	4.119146	9.990857
H	2.905183	2.768424	10.476875
H	2.339166	3.881425	9.242400
H	4.002707	4.140026	12.547034
H	2.674951	4.835690	13.476084
H	2.573320	3.161442	12.868906

C7

O	-2.531523	2.366976	8.095540
O	-1.613274	2.369692	10.141686
O	-3.124400	5.508402	12.242868
O	-0.788009	6.705860	7.196727
O	-0.745232	7.423888	9.336871
O	-1.584037	6.923682	11.421569
O	0.095456	4.186844	8.212422
O	0.371880	5.366645	12.627413
C	-2.475134	4.426361	9.404740
C	-2.222204	2.962772	9.082425
C	-2.185848	5.706367	11.533519
C	-1.529276	4.722494	10.576421
C	-1.291717	3.313352	11.168548
C	0.162074	3.283654	11.481143
C	0.757457	4.657835	11.428428
C	-0.132297	5.358650	10.347827
C	0.320415	5.381324	8.897380
C	-0.467733	6.557215	8.340533
C	-0.443388	6.852684	10.617140
C	2.304852	4.680878	11.289193
C	2.817576	6.116773	11.103667
C	2.790110	3.750629	10.164153
C	2.916112	4.162916	12.605494
H	-0.129828	4.408555	7.290795
H	0.561920	4.798810	13.389176
H	-2.366497	5.037774	8.509915
H	-3.511278	4.515025	9.752805
H	-1.949916	3.118284	12.024575
H	0.683794	2.382352	11.774972
H	1.376617	5.677198	8.832884
H	0.341769	7.441537	11.087491
H	3.911360	6.124322	11.150219
H	2.537293	6.564189	10.145316
H	2.442998	6.759194	11.906784
H	3.861085	3.910061	10.000028
H	2.654580	2.701171	10.444451
H	2.274391	3.887671	9.211838
H	3.998622	4.056546	12.484861
H	2.746565	4.859479	13.432166
H	2.523593	3.178599	12.885035

C8tBu

O	-2.795181	2.328409	8.272061
O	-1.830538	2.382927	10.295268
O	-3.095525	5.602702	12.239154
O	-0.799817	6.641432	7.154369
O	-0.716829	7.397871	9.281188
O	-1.514197	6.958253	11.396796
O	0.075080	4.128715	8.205866
O	0.320160	5.247067	12.591376
C	-2.512155	4.456235	9.431779
C	-2.405853	2.957582	9.209088
C	-2.153345	5.759883	11.525463
C	-1.531935	4.743164	10.578006
C	-1.316498	3.344329	11.212298
C	0.198623	3.186564	11.342636
C	0.748997	4.617542	11.383774
C	-0.125397	5.342491	10.318182
C	0.313976	5.335617	8.865716
C	-0.466628	6.510207	8.296817
C	-0.397147	6.846208	10.568905
C	2.312341	4.682298	11.293068
C	2.786760	6.138260	11.152611
C	2.878985	3.801440	10.160952
C	2.864340	4.152180	12.592938
H	-0.151252	4.334781	7.280936
H	0.722211	4.767246	13.333359
H	-2.352001	4.989191	8.495876
H	-3.534954	4.662048	9.768226
H	-1.860666	3.247176	12.154938
H	0.545522	2.668079	10.443701
H	0.503565	2.607619	12.220110
H	1.372769	5.624129	8.789516
H	0.409314	7.430603	11.006460
H	3.878055	6.176917	11.220186
H	2.505296	6.588276	10.195322
H	2.374093	6.751663	11.959661
H	3.942498	4.024065	10.031265
H	2.795695	2.740531	10.414220
H	2.379155	3.937954	9.199129
H	3.191793	4.826816	13.376394
H	3.040484	3.091070	12.728956

C10

O	-2.856341	2.100277	8.466611
O	-1.899961	2.302027	10.483519
O	-3.227956	5.585617	12.106506
O	-0.069436	6.947287	7.198994
O	-0.434911	7.489983	9.387535
O	-1.622855	6.929174	11.295185
O	0.365511	4.291370	8.128884
O	0.281964	5.204619	12.615616
C	-2.454819	4.317160	9.402708
C	-2.438484	2.797132	9.340859
C	-2.245401	5.730413	11.444928
C	-1.557692	4.675405	10.593030
C	-1.331505	3.315359	11.305161
C	0.188306	3.147250	11.373989
C	0.739196	4.576762	11.412885
C	-0.150291	5.296612	10.346379
C	0.229008	5.353501	8.924156
C	-0.087307	6.649011	8.370095
C	-0.391363	6.809639	10.636486
C	2.286926	4.695338	11.297719
C	2.710368	6.153545	11.077943
C	2.857832	3.823618	10.170185
C	2.918070	4.220413	12.619048
H	0.377992	4.624489	7.207846
H	0.563287	4.669448	13.372169
H	-2.155164	4.734638	8.440712
H	-3.489752	4.619721	9.594381
H	-1.828387	3.283903	12.277278
H	0.503797	2.642516	10.455098
H	0.516089	2.541022	12.224448
H	0.360502	7.286060	11.259504

H	3.802996	6.220162	11.066427
H	2.351030	6.547076	10.119916
H	2.347554	6.795827	11.886289
H	3.934893	4.004200	10.093244
H	2.720320	2.757304	10.375493
H	2.413981	4.041273	9.196942
H	4.007670	4.215715	12.518950
H	2.674557	4.889187	13.450723
H	2.614024	3.199973	12.878998

C12

O	-2.861032	2.397035	8.242591
O	-1.837351	2.374186	10.237336
O	-2.992770	5.592465	12.358617
O	-0.614833	6.733161	7.156977
O	-0.596470	7.404808	9.318467
O	-1.462910	6.944889	11.389085
O	0.021341	4.109347	8.192042
O	0.271279	5.157704	12.605899
C	-2.528875	4.483340	9.463838
C	-2.440433	2.990389	9.188804
C	-2.098384	5.731945	11.587495
C	-1.530264	4.714814	10.603046
C	-1.311729	3.297459	11.185787
C	0.207980	3.135957	11.300929
C	0.747620	4.571014	11.392215
C	-0.118718	5.302033	10.320437
C	0.332704	5.290556	8.869204
C	-0.347203	6.529709	8.302891
C	-0.401305	6.775736	10.549450
C	2.294613	4.705689	11.316022
C	2.692072	6.179027	11.132503
C	2.906661	3.808410	10.224557
C	2.895085	4.246763	12.658834
H	-0.110318	4.323793	7.251743
H	0.526896	4.587960	13.346169
H	-2.362696	5.044979	8.544649
H	-3.546656	4.694186	9.812126
H	-1.847277	3.165553	12.129714
H	0.554881	2.651345	10.383252
H	0.511622	2.518920	12.152610
H	1.407819	5.506858	8.811936
H	3.771197	6.290845	11.280900
H	2.462316	6.572854	10.137585
H	2.176385	6.804556	11.867754
H	3.951462	4.093794	10.064235
H	2.898542	2.759175	10.538036
H	2.392460	3.853291	9.261175
H	3.985505	4.211561	12.573460
H	2.653945	4.941602	13.468849
H	2.561719	3.240000	12.937343

C8OH

O	-2.717056	2.337445	8.224457
O	-1.782208	2.383160	10.262803
O	-3.107417	5.595638	12.226084
O	-0.793449	6.634046	7.156522
O	-0.739970	7.388477	9.284764
O	-1.507017	6.950575	11.415887
O	0.094668	4.127895	8.219858
O	0.320631	5.115211	12.631124
C	-2.500474	4.453027	9.421796
C	-2.358150	2.960971	9.176165
C	-2.158534	5.753875	11.523340
C	-1.529600	4.741904	10.575185
C	-1.321151	3.342910	11.207114
C	0.185634	3.188714	11.399178
C	0.759340	4.643805	11.416260
C	-0.124672	5.343229	10.323697
C	0.324860	5.339074	8.874461
C	-0.466536	6.505194	8.300344
C	-0.403641	6.846611	10.569767
C	2.318039	4.694978	11.318970
C	2.781334	6.153709	11.173186
C	2.875487	3.821878	10.180360
C	2.905766	4.165127	12.637548
H	-0.118231	4.324461	7.289713
H	-2.351738	5.004578	8.494847
H	-3.527248	4.631258	9.762171
H	-1.897447	3.229926	12.129105
H	0.591365	2.684355	10.516850
H	0.460166	2.635273	12.297882
H	1.381869	5.634343	8.803031
H	0.404433	7.441113	10.993708
H	3.873275	6.199135	11.232086
H	2.493290	6.611986	10.221138
H	2.381487	6.767460	11.988679
H	3.925449	4.081536	10.010116
H	2.846186	2.762982	10.456102
H	2.345738	3.917286	9.229690
H	3.998333	4.165230	12.566039
H	2.611692	4.784339	13.486970
H	2.590038	3.136343	12.836602

C10OH

O	-2.910007	2.218670	8.397411
O	-1.876712	2.349399	10.381770
O	-3.184389	5.549987	12.155024
O	-0.614683	6.898816	7.198439
O	-0.656725	7.449431	9.380955
O	-1.563013	6.921796	11.425590
O	0.074221	4.240416	8.148869
O	0.298383	5.195805	12.621608
C	-2.455876	4.403368	9.383295
C	-2.452772	2.884180	9.274798
C	-2.207842	5.724969	11.492906
C	-1.533024	4.720101	10.570260
C	-1.314184	3.333816	11.237257
C	0.202655	3.167416	11.319971
C	0.750297	4.598090	11.402652
C	-0.129226	5.341507	10.351088
C	0.294001	5.357022	8.886967
C	-0.382555	6.636833	8.334458
C	-0.406968	6.832778	10.646560
C	2.303488	4.695853	11.307524
C	2.756225	6.150879	11.114696
C	2.885930	3.787430	10.209313
C	2.909753	4.218903	12.641668
H	0.566607	4.633271	13.363144
H	-2.188175	4.839863	8.418444
H	-3.483913	4.707628	9.607933
H	-1.820871	3.275058	12.203375
H	0.526914	2.688512	10.390963
H	0.522805	2.541128	12.158768
H	1.373307	5.568424	8.771144
H	0.379240	7.388334	11.152294
H	3.848729	6.205265	11.156452
H	2.453858	6.582285	10.153836
H	2.360409	6.783953	11.914893
H	3.948349	4.015980	10.077748
H	2.811665	2.733482	10.494677
H	2.403309	3.887539	9.234251
H	3.998945	4.170892	12.547852
H	2.685062	4.909510	13.460064
H	2.566093	3.214757	12.914923

Table S12 Coordinates and imaginary frequencies of the transition state of the HAT from C10 site of **1B** to each ROS. Level of theory: M06-2X/6-31G(d).

$^{\bullet}$OH

ν_{im}= -1525.7306 cm^{-1}

C	-0.014177	-0.059857	0.088581
C	0.313644	1.394372	-0.251846
C	-0.980502	1.952586	-0.872526
C	-1.652441	0.714855	-1.529755
C	-0.885280	-0.531975	-1.080122
C	-0.097608	-0.974958	-2.340006
C	-0.562557	-2.395932	-2.603827
C	-2.049874	-2.368568	-2.216495
C	-2.017649	-1.601375	-0.864694
C	-1.867738	-2.355319	0.447522
C	-2.415101	-1.380921	1.482143
C	-3.219901	-0.682310	-0.573448
C	-1.460084	0.767821	-3.048528
C	-1.692775	2.518810	0.361508
C	-0.523878	2.881885	1.272343
C	-2.631493	3.688339	0.114024
C	-2.797330	-3.719371	-2.351934
C	-4.236817	-3.595313	-1.831425
C	-2.071500	-4.885906	-1.665215
C	-2.885925	-4.044060	-3.853891
O	1.109043	-0.909751	0.242776
O	-0.619192	2.949653	-1.791529
O	-0.691251	-2.994066	0.760476
O	-2.213814	-1.374488	2.660123
O	-3.214600	-0.479837	0.851342
O	-3.024979	0.533991	-1.226315
O	-0.525881	-0.122495	-3.420193
O	-2.032554	1.500982	-3.802464
O	-0.509810	3.644889	2.187726
O	0.589552	2.161967	0.907694
H	-0.587949	-0.038278	1.025744
H	1.160484	1.472039	-0.941367
H	0.983479	-0.865143	-2.243863
H	-0.373913	-2.693220	-3.637515
H	-0.015592	-3.068616	-1.932558
H	-2.555363	-1.685061	-2.919681
H	-2.693039	-3.225297	0.532133
H	-4.202867	-1.059741	-0.855040
H	-2.241991	1.711609	0.866423
H	-3.483463	3.367022	-0.491554
H	-2.107758	4.499595	-0.396487
H	-3.000214	4.067775	1.069087
H	-4.297528	-3.422947	-0.753522
H	-4.780815	-4.524201	-2.031271
H	-4.767471	-2.786625	-2.349808
H	-1.917555	-4.741982	-0.595012
H	-1.096651	-5.067605	-2.130053
H	-2.666422	-5.797216	-1.788223
H	-3.395311	-3.246238	-4.406111
H	-3.453517	-4.968939	-3.999299
H	-1.897545	-4.192380	-4.299708
H	0.072647	-2.385893	0.677826
H	1.654733	-0.564950	0.968307
H	-1.384934	3.156841	-2.353981
O	-3.393121	-4.108578	1.291104
H	-2.852496	-4.047230	2.106573

$^{\bullet}$OOH

ν_{im}= -1970.2663 cm^{-1}

C	0.074760	-0.059889	0.018004
C	0.331867	1.419619	-0.273966
C	-1.000563	1.952759	-0.836146
C	-1.655695	0.715920	-1.510428
C	-0.839981	-0.517826	-1.121112
C	-0.099105	-0.920080	-2.422359
C	-0.524336	-2.354793	-2.681475
C	-1.989255	-2.388639	-2.220558
C	-1.929975	-1.624076	-0.864134
C	-1.711554	-2.351559	0.445546
C	-2.334376	-1.460847	1.492261
C	-3.160985	-0.749107	-0.544703
C	-1.522144	0.811017	-3.033957
C	-1.691416	2.455166	0.436793
C	-0.507770	2.827101	1.324132
C	-2.675811	3.599147	0.256959
C	-2.690664	-3.764325	-2.325221
C	-4.123973	-3.678388	-1.779948
C	-1.900883	-4.891545	-1.641380
C	-2.802923	-4.116164	-3.819562
O	1.233232	-0.876255	0.057173
O	-0.701666	2.988959	-1.733898
O	-0.518989	-2.936374	0.759256
O	-2.183102	-1.500816	2.681457
O	-3.164159	-0.576146	0.880159
O	-3.013306	0.486194	-1.174356
O	-0.607932	-0.071930	-3.469155
O	-2.126743	1.561105	-3.744745
O	-0.490390	3.560131	2.263739
O	0.615669	2.155781	0.903797
H	-0.444995	-0.111822	0.986129
H	1.152631	1.555132	-0.985617
H	0.980740	-0.772906	-2.375906
H	-0.378935	-2.630547	-3.727980
H	0.083993	-3.018177	-2.054097
H	-2.551985	-1.720752	-2.894752
H	-2.553996	-3.385430	0.648727
H	-4.129836	-1.157733	-0.831771
H	-2.197838	1.614018	0.930849
H	-3.535699	3.268282	-0.331989
H	-2.195684	4.443305	-0.242925
H	-3.025859	3.935458	1.234927
H	-4.172422	-3.476962	-0.707611
H	-4.637291	-4.631537	-1.942959
H	-4.691089	-2.904262	-2.312094
H	-0.977390	-5.103471	-2.191396
H	-2.499763	-5.807915	-1.641587
H	-1.622557	-4.680589	-0.607307
H	-3.356629	-3.347215	-4.370084
H	-3.338481	-5.064055	-3.936015
H	-1.821424	-4.234526	-4.288673
H	0.238908	-2.407482	0.432306
H	1.789708	-0.586690	0.798189
H	-1.489163	3.182175	-2.270750
O	-4.085095	-3.534956	1.957492
H	-3.633496	-3.317001	2.796439
O	-3.131586	-4.237199	1.258103

$^{\bullet}$OCH$_3$

ν_{im}= -1864.0418 cm^{-1}

C	0.152888	-0.189558	0.164608
C	0.566617	1.241066	-0.181422
C	-0.670099	1.846125	-0.871541
C	-1.362438	0.629111	-1.546815
C	-0.672612	-0.641916	-1.043780
C	0.163098	-1.132516	-2.252844
C	-0.347756	-2.535078	-2.529306
C	-1.851506	-2.437313	-2.226204
C	-1.860989	-1.661847	-0.877641
C	-1.802352	-2.393698	0.451833
C	-2.340513	-1.382953	1.442835
C	-3.035157	-0.689671	-0.656028
C	-1.088166	0.653221	-3.053773
C	-1.415813	2.459096	0.319735
C	-0.277189	2.785166	1.281600
C	-2.290397	3.663778	0.013076
C	-2.652722	-3.750695	-2.403390
C	-4.116510	-3.546773	-1.987816
C	-2.032538	-4.939958	-1.655960
C	-2.652630	-4.090905	-3.904586
O	1.222461	-1.088327	0.402649
O	-0.221533	2.814646	-1.782801
O	-0.685221	-3.103382	0.812165

O	-2.167998	-1.337832	2.626063
O	-3.093270	-0.472145	0.764089
O	-2.754526	0.511022	-1.310549
O	-0.168522	-0.275513	-3.363417
O	-1.590474	1.396525	-3.846933
O	-0.274129	3.559959	2.187278
O	0.820905	2.014618	0.979510
H	-0.470693	-0.119499	1.066097
H	1.448503	1.269629	-0.829489
H	1.241075	-1.067428	-2.098173
H	-0.115956	-2.850352	-3.548791
H	0.130841	-3.223757	-1.823030
H	-2.283587	-1.735159	-2.959425
H	-2.728623	-3.243339	0.551316
H	-4.017558	-1.027381	-0.985525
H	-2.023254	1.682957	0.806409
H	-3.125682	3.369987	-0.628636
H	-1.708740	4.444678	-0.481835
H	-2.687366	4.072237	0.944547
H	-4.240757	-3.357620	-0.919069
H	-4.692840	-4.449420	-2.216047
H	-4.565464	-2.719331	-2.551807
H	-1.925654	-4.772696	-0.583806
H	-1.045585	-5.184959	-2.062619
H	-2.670436	-5.820206	-1.789355
H	-3.078344	-3.274649	-4.499113
H	-3.258271	-4.985970	-4.080439
H	-1.645253	-4.297973	-4.278547
H	0.108275	-2.527133	0.823877
H	1.747869	-0.743988	1.143218
H	-0.948735	3.044974	-2.385858
O	-3.533271	-4.044545	1.118774
C	-2.873785	-4.592175	2.226240
H	-3.630906	-5.208747	2.731367
H	-2.030929	-5.230687	1.937161
H	-2.541121	-3.817789	2.928107

•OOCH$_3$

ν_{im}= -1958.2931 cm^{-1}

C	0.105015	-0.019461	-0.042849
C	0.341091	1.458263	-0.360422
C	-1.020043	1.973610	-0.868808
C	-1.684729	0.727767	-1.516401
C	-0.842451	-0.494155	-1.147395
C	-0.132685	-0.889209	-2.467291
C	-0.544789	-2.329667	-2.709385
C	-1.997256	-2.378363	-2.212402
C	-1.913202	-1.614115	-0.855167
C	-1.644859	-2.324929	0.454804
C	-2.185656	-1.390895	1.506938
C	-3.144143	-0.752404	-0.499863
C	-1.596997	0.818674	-3.043743
C	-1.664175	2.467366	0.432084
C	-0.448792	2.862249	1.265363
C	-2.674293	3.594379	0.292909
C	-2.691020	-3.757410	-2.321770
C	-4.123770	-3.678993	-1.776496
C	-1.901702	-4.883801	-1.639001
C	-2.798779	-4.104304	-3.817858
O	1.267491	-0.832464	-0.029222
O	-0.772259	3.014419	-1.776866
O	-0.448417	-2.930357	0.714928
O	-1.880309	-1.309930	2.661935
O	-3.104412	-0.571800	0.921699
O	-3.028435	0.484093	-1.139140
O	-0.681506	-0.051446	-3.502131
O	-2.233312	1.556163	-3.739925
O	-0.401534	3.600847	2.199631
O	0.666172	2.207322	0.798638
H	-0.378423	-0.058948	0.942776
H	1.130322	1.595514	-1.106565
H	0.945856	-0.726930	-2.450685
H	-0.422663	-2.608201	-3.758013
H	0.087258	-2.981900	-2.094039
H	-2.579662	-1.710227	-2.869470
H	-2.535407	-3.355200	0.707730
H	-4.116196	-1.171395	-0.759561
H	-2.134968	1.619671	0.950107
H	-3.554331	3.246783	-0.255136
H	-2.231271	4.444102	-0.231252
H	-2.985148	3.928974	1.284612
H	-4.166196	-3.491037	-0.701419
H	-4.637276	-4.630316	-1.950303
H	-4.691933	-2.898428	-2.298111
H	-1.689211	-4.703551	-0.583906
H	-0.945241	-5.050851	-2.146319
H	-2.472674	-5.815982	-1.704395
H	-3.332269	-3.323386	-4.371652
H	-3.353504	-5.040414	-3.939300
H	-1.816900	-4.243431	-4.280260
H	0.302344	-2.384853	0.395336
H	1.846769	-0.526331	0.687183
H	-1.581519	3.192086	-2.285903
O	-3.412516	-3.799558	2.461705
O	-3.141187	-4.232709	1.190697
C	-2.360123	-4.229765	3.321369
H	-2.636347	-3.857529	4.308693
H	-2.302000	-5.320982	3.315392
H	-1.413310	-3.784562	3.004205

•OOCH=CH$_2$

ν_{im}= -1950.3458 cm^{-1}

C	-0.002035	-0.286027	0.045370
C	0.305363	1.201318	-0.137323
C	-1.007010	1.810815	-0.668970
C	-1.682262	0.649191	-1.448755
C	-0.905458	-0.631757	-1.140968
C	-0.144291	-0.948921	-2.453109
C	-0.598015	-2.346771	-2.832604
C	-2.074518	-2.375377	-2.410295
C	-2.030153	-1.726784	-0.992975
C	-1.853087	-2.550815	0.264624
C	-2.417843	-1.688106	1.365620
C	-3.245340	-0.847466	-0.628451
C	-1.512498	0.858800	-2.957345
C	-1.700732	2.224802	0.634114
C	-0.517752	2.502062	1.556518
C	-2.660520	3.399234	0.537371
C	-2.809725	-3.714288	-2.660127
C	-4.251104	-3.641872	-2.137299
C	-2.067614	-4.921264	-2.068182
C	-2.896042	-3.919707	-4.183241
O	1.127066	-1.144783	0.054014
O	-0.672898	2.909565	-1.475441
O	-0.697391	-3.228775	0.525164
O	-2.184862	-1.716508	2.537919
O	-3.275264	-0.785599	0.801325
O	-3.051381	0.431052	-1.158087
O	-0.606509	-0.008131	-3.440544
O	-2.085138	1.674134	-3.621123
O	-0.493202	3.161298	2.549211
O	0.595569	1.841264	1.094196
H	-0.540591	-0.383628	0.997815
H	1.137850	1.365617	-0.828969
H	0.937420	-0.834404	-2.371048
H	-0.432367	-2.546092	-3.893418
H	-0.023630	-3.071269	-2.242294
H	-2.599628	-1.634457	-3.036883
H	-2.778334	-3.572176	0.397167
H	-4.216598	-1.203539	-0.972117
H	-2.228852	1.358172	1.056900
H	-3.521884	3.132550	-0.081192
H	-2.159735	4.269898	0.108102
H	-3.011791	3.665409	1.536267
H	-4.316454	-3.535759	-1.051706
H	-4.785034	-4.561718	-2.397233
H	-4.788514	-2.806934	-2.604217
H	-1.855953	-4.829056	-1.001691
H	-1.115246	-5.082078	-2.585023
H	-2.672088	-5.823589	-2.208488
H	-3.408017	-3.082371	-4.670672
H	-3.461335	-4.831792	-4.401006

H	-1.907704	-4.029963	-4.639597
H	0.091167	-2.683598	0.313026
H	1.676168	-0.920171	0.822567
H	-1.449340	3.165803	-2.001704
O	-3.388899	-4.497862	0.782674
C	-2.279966	-4.956613	2.694397
C	-1.915193	-4.648202	3.930435
H	-1.801463	-5.671299	2.033889
H	-2.439306	-3.883986	4.491760
H	-1.076897	-5.158099	4.386810
O	-3.393945	-4.359754	2.146628

Table S13 Coordinates and imaginary frequencies of the transition state of the HAT from C10OH site of **1B** to each ROS. Level of theory: M06-2X/6-31G(d).

$^{\bullet}$OH

ν_{im}= -1932.2254 cm^{-1}

C	0.249242	-0.113370	-0.192584
C	0.437105	1.388800	-0.446263
C	-0.926453	1.937768	-0.898257
C	-1.631894	0.723713	-1.552230
C	-0.808422	-0.530343	-1.250549
C	-0.180453	-0.909210	-2.613628
C	-0.634682	-2.334053	-2.870797
C	-2.067401	-2.360853	-2.314651
C	-1.901808	-1.615531	-0.959523
C	-1.634683	-2.439840	0.297486
C	-2.045801	-1.456197	1.406760
C	-3.089971	-0.737905	-0.530644
C	-1.622299	0.850379	-3.078840
C	-1.527574	2.414208	0.428871
C	-0.282174	2.758206	1.240039
C	-2.515715	3.565459	0.345199
C	-2.785458	-3.733788	-2.387926
C	-4.157467	-3.676054	-1.699095
C	-1.946330	-4.891386	-1.823798
C	-3.046885	-4.037270	-3.874175
O	1.466641	-0.788848	-0.306855
O	-0.693449	2.995197	-1.793402
O	-0.365458	-2.974263	0.351952
O	-1.667300	-1.436464	2.546828
O	-2.956224	-0.610355	0.914161
O	-2.974769	0.504225	-1.122911
O	-0.762964	-0.035390	-3.603869
O	-2.278383	1.619556	-3.722069
O	-0.207100	3.468453	2.197424
O	0.797543	2.097987	0.730897
H	-0.150807	-0.213456	0.825196
H	1.210469	1.555117	-1.202136
H	0.900711	-0.772955	-2.640936
H	-0.561375	-2.596607	-3.928495
H	0.009469	-3.001616	-2.286759
H	-2.669715	-1.682920	-2.942484
H	-2.381751	-3.244673	0.393566
H	-4.092152	-1.121395	-0.721516
H	-1.999789	1.565183	0.941350
H	-3.419997	3.253961	-0.185399
H	-2.067494	4.416768	-0.172008
H	-2.788223	3.885039	1.353105
H	-4.098202	-3.523559	-0.616016
H	-4.692985	-4.617646	-1.857931
H	-4.773202	-2.873182	-2.122142
H	-1.627308	-4.750791	-0.788221
H	-1.042911	-5.045874	-2.422016
H	-2.529985	-5.817048	-1.869747
H	-3.654833	-3.253291	-4.338897
H	-3.585671	-4.985438	-3.973111
H	-2.114935	-4.127070	-4.439738
H	0.159116	-2.977095	1.351909
H	1.529296	-1.416549	0.432100
H	-1.521018	3.206667	-2.257529
O	0.853140	-2.339910	2.117661
H	0.208284	-1.915848	2.724711

$^{\bullet}$OOH

ν_{im}= -1526.3843 cm^{-1}

C	-0.034505	-0.251583	0.175708
C	0.308859	1.226997	-0.059729
C	-0.923664	1.851058	-0.736557
C	-1.590315	0.665302	-1.480380
C	-0.904782	-0.628539	-1.033097
C	-0.088021	-1.075089	-2.272620
C	-0.578959	-2.478802	-2.571403
C	-2.081853	-2.406757	-2.257879
C	-2.089398	-1.644384	-0.900670
C	-2.112334	-2.469567	0.387898
C	-2.679950	-1.469769	1.424208
C	-3.273538	-0.687952	-0.682751
C	-1.327495	0.755103	-2.986727
C	-1.698934	2.394964	0.468817
C	-0.583567	2.676574	1.470859
C	-2.579672	3.606000	0.210108
C	-2.863820	-3.734765	-2.442469
C	-4.329096	-3.578693	-2.007505
C	-2.216486	-4.936749	-1.735953
C	-2.881154	-4.047273	-3.950166
O	1.087766	-1.092412	0.245821
O	-0.478522	2.869024	-1.595653
O	-1.012571	-3.184715	0.745367
O	-2.589827	-1.497236	2.617606
O	-3.371297	-0.530589	0.757756
O	-2.992370	0.532832	-1.268598
O	-0.454909	-0.194855	-3.355303
O	-1.819515	1.549110	-3.737018
O	-0.615322	3.401623	2.418331
O	0.520722	1.935131	1.153077
H	-0.610319	-0.304184	1.111036
H	1.202844	1.324981	-0.683235
H	0.990298	-0.998246	-2.134108
H	-0.348628	-2.777474	-3.596338
H	-0.080929	-3.161731	-1.873164
H	-2.528633	-1.706784	-2.983690
H	-2.941934	-3.199554	0.323742
H	-4.251365	-1.019027	-1.034086
H	-2.304772	1.589156	0.905090
H	-3.402947	3.341620	-0.459584
H	-1.996248	4.415063	-0.235005
H	-2.992931	3.964158	1.155186
H	-4.454520	-3.412440	-0.931862
H	-4.888129	-4.487472	-2.252818
H	-4.807175	-2.745745	-2.536918
H	-2.115993	-4.824463	-0.653728
H	-1.217083	-5.134066	-2.135921
H	-2.822973	-5.830320	-1.918626
H	-3.330083	-3.227268	-4.521364
H	-3.470250	-4.951013	-4.137491
H	-1.874514	-4.224092	-4.339769
H	-0.140268	-2.697119	1.671778
H	1.453295	-1.036177	1.143254
H	-1.201936	3.117147	-2.195971
O	0.170713	-2.113511	2.490859
O	-0.327405	-2.776401	3.593040
H	-1.167400	-2.296110	3.739565

$^{\bullet}$OCH$_3$

ν_{im}= -1989.3601 cm^{-1}

C	0.216192	-0.144210	-0.166648
C	0.430259	1.358995	-0.404196
C	-0.918581	1.923006	-0.881970
C	-1.621983	0.716461	-1.553690
C	-0.818887	-0.547053	-1.233376
C	-0.175906	-0.942435	-2.586982
C	-0.635646	-2.366928	-2.830322
C	-2.076409	-2.373278	-2.296594
C	-1.920989	-1.620837	-0.941983

C	-1.730069	-2.456129	0.328783
C	-2.182452	-1.482262	1.440356
C	-3.110635	-0.725567	-0.547764
C	-1.579811	0.840173	-3.080016
C	-1.541018	2.399448	0.435447
C	-0.309972	2.730799	1.272381
C	-2.519046	3.557991	0.336575
C	-2.810574	-3.737654	-2.383908
C	-4.206363	-3.658220	-1.746177
C	-2.009347	-4.906200	-1.787853
C	-3.026128	-4.045924	-3.877120
O	1.393065	-0.896507	-0.312203
O	-0.658955	2.981945	-1.768343
O	-0.541884	-3.106469	0.521604
O	-1.903137	-1.512234	2.598912
O	-3.019090	-0.578663	0.887638
O	-2.973963	0.510257	-1.157837
O	-0.740824	-0.073373	-3.590671
O	-2.201843	1.626651	-3.736481
O	-0.243572	3.440785	2.230269
O	0.774085	2.057745	0.784520
H	-0.190905	-0.260192	0.847641
H	1.219311	1.521963	-1.144482
H	0.905904	-0.812658	-2.608217
H	-0.544857	-2.647661	-3.881924
H	-0.006312	-3.027437	-2.222000
H	-2.659200	-1.691247	-2.938449
H	-2.524244	-3.219789	0.367041
H	-4.108291	-1.105343	-0.771667
H	-2.028792	1.551362	0.934753
H	-3.415039	3.253152	-0.211518
H	-2.055736	4.407305	-0.170507
H	-2.808642	3.876583	1.340041
H	-4.187758	-3.502599	-0.662341
H	-4.748060	-4.593291	-1.922329
H	-4.794395	-2.848524	-2.194780
H	-1.744899	-4.780088	-0.735326
H	-1.077186	-5.061423	-2.340229
H	-2.596602	-5.826838	-1.874548
H	-3.594915	-3.249476	-4.369527
H	-3.587336	-4.979620	-3.988437
H	-2.077609	-4.166661	-4.408203
H	0.300321	-2.561537	1.166034
H	1.758117	-1.059660	0.571512
H	-1.470913	3.188403	-2.261594
O	0.839672	-2.079175	2.046241
C	0.788710	-2.898634	3.179892
H	-0.167599	-2.790718	3.698239
H	0.992144	-3.946304	2.935221
H	1.583812	-2.529699	3.842820

•OOCH$_3$

ν_{im}= -1295.5958 cm^{-1}

C	-0.055925	-0.250285	0.188671
C	0.280722	1.235043	-0.025748
C	-0.927205	1.863634	-0.741608
C	-1.576481	0.682533	-1.506053
C	-0.921429	-0.617325	-1.031718
C	-0.084582	-1.087349	-2.249309
C	-0.580198	-2.490827	-2.537580
C	-2.088733	-2.401478	-2.261755
C	-2.124198	-1.615942	-0.916769
C	-2.197198	-2.426001	0.381200
C	-2.809177	-1.396927	1.387790
C	-3.304806	-0.642513	-0.756485
C	-1.267950	0.762188	-3.004642
C	-1.738803	2.412083	0.437353
C	-0.655566	2.689067	1.474646
C	-2.606309	3.625890	0.149343
C	-2.875536	-3.726771	-2.443312
C	-4.350582	-3.553863	-2.049301
C	-2.251413	-4.918453	-1.699187
C	-2.858182	-4.066570	-3.945052
O	1.081290	-1.070834	0.243425
O	-0.450709	2.880003	-1.586436
O	-1.100246	-3.084421	0.811370
O	-2.767844	-1.429987	2.576739
O	-3.458734	-0.460923	0.672534
O	-2.987696	0.566197	-1.348942
O	-0.421198	-0.218421	-3.350774
O	-1.715577	1.569399	-3.769020
O	-0.714469	3.416912	2.419222
O	0.452250	1.939702	1.195726
H	-0.634999	-0.318300	1.122316
H	1.194902	1.336175	-0.618392
H	0.990933	-1.017689	-2.089370
H	-0.327383	-2.809830	-3.551081
H	-0.103910	-3.164824	-1.815472
H	-2.511713	-1.709338	-3.009112
H	-3.030143	-3.150508	0.316535
H	-4.270835	-0.969173	-1.143351
H	-2.361156	1.609391	0.855355
H	-3.409855	3.363126	-0.544483
H	-2.006166	4.431154	-0.280121
H	-3.046334	3.988092	1.080735
H	-4.502860	-3.364423	-0.981278
H	-4.908667	-4.464138	-2.291476
H	-4.809147	-2.729259	-2.608198
H	-2.155657	-4.776445	-0.620050
H	-1.252314	-5.140194	-2.087177
H	-2.868839	-5.808449	-1.861814
H	-3.291736	-3.256014	-4.541125
H	-3.445130	-4.972341	-4.129729
H	-1.843302	-4.252370	-4.308075
H	-0.271925	-2.513877	1.745581
H	1.340614	-1.180031	1.172743
H	-1.149093	3.120391	-2.218692
O	0.137551	-2.023439	2.582654
O	0.316878	-3.036175	3.501209
C	-0.776968	-3.005467	4.404782
H	-0.876799	-2.011005	4.846166
H	-1.699687	-3.265002	3.876098
H	-0.533558	-3.751148	5.163320

•OOCH=CH$_2$

ν_{im}= -1117.3466 cm^{-1}

C	-0.074014	-0.334279	0.191409
C	0.272212	1.154427	0.022979
C	-0.931341	1.810154	-0.675841
C	-1.581175	0.656153	-1.480487
C	-0.932004	-0.660169	-1.045706
C	-0.091423	-1.094542	-2.273443
C	-0.589768	-2.487031	-2.607185
C	-2.099319	-2.403126	-2.334331
C	-2.138495	-1.658047	-0.967618
C	-2.198712	-2.498843	0.310380
C	-2.799977	-1.491405	1.341609
C	-3.316323	-0.687836	-0.779651
C	-1.265773	0.782242	-2.974432
C	-1.745598	2.320968	0.518415
C	-0.666702	2.556957	1.570140
C	-2.607171	3.547508	0.268855
C	-2.886586	-3.722370	-2.551919
C	-4.359322	-3.560817	-2.145423
C	-2.258989	-4.933841	-1.843627
C	-2.875621	-4.018821	-4.062642
O	1.055106	-1.166604	0.233753
O	-0.449983	2.853016	-1.484725
O	-1.095117	-3.153891	0.711916
O	-2.723844	-1.534760	2.529568
O	-3.473027	-0.550680	0.655372
O	-2.993350	0.539233	-1.332019
O	-0.420797	-0.189504	-3.348106
O	-1.706874	1.615578	-3.714393
O	-0.729679	3.246714	2.542876
O	0.442120	1.818304	1.267930
H	-0.660271	-0.425422	1.117634
H	1.188902	1.271148	-0.562884
H	0.983742	-1.033250	-2.107195
H	-0.333790	-2.775470	-3.629111
H	-0.118275	-3.183435	-1.903625
H	-2.519623	-1.690334	-3.063642

H	-3.034379	-3.223600	0.242779
H	-4.282995	-0.997404	-1.178151
H	-2.373415	1.507054	0.905480
H	-3.409699	3.311123	-0.435520
H	-2.002465	4.363844	-0.132302
H	-3.048498	3.880311	1.210558
H	-4.504471	-3.401680	-1.071401
H	-4.919019	-4.463891	-2.409855
H	-4.821045	-2.720657	-2.677960
H	-2.149451	-4.816282	-0.762914
H	-1.265298	-5.149879	-2.248423
H	-2.881718	-5.817554	-2.019736
H	-3.318127	-3.194512	-4.632723
H	-3.457157	-4.923133	-4.270323
H	-1.861342	-4.186474	-4.436196
H	-0.318617	-2.690971	1.790315
H	1.346194	-1.253355	1.155470
H	-1.143052	3.110553	-2.116140
O	0.174147	-2.185636	2.559010
O	0.093980	-3.056154	3.628883
C	-0.780900	-2.585654	4.564051
H	-1.144529	-1.584039	4.372654
C	-1.090450	-3.355122	5.605555
H	-1.753697	-2.975860	6.371177
H	-0.683857	-4.354402	5.708787

Table S14 Coordinates and imaginary frequencies of the transition state of the HAT from C10 site of **1B**$^-$ to each ROS. Level of theory: M06-2X/6-31G(d).

•OH

ν_{im}= -1173.4858 cm^{-1}

C	-0.041078	-0.215087	-1.742643
C	-1.352293	0.478826	-1.362449
C	-1.914540	-0.417337	-0.217917
C	-0.641633	-0.969924	0.492625
C	0.577194	-0.602615	-0.375946
C	1.369681	-1.927445	-0.380467
C	2.449656	-1.753941	0.689850
C	2.841559	-0.265223	0.648844
C	1.432307	0.406746	0.466468
C	1.442150	1.902378	0.123966
C	0.387225	2.569665	0.987622
C	0.634980	0.442390	1.823533
C	-0.625113	-2.502243	0.610072
C	-2.900147	0.327811	0.686937
C	-3.899931	0.936208	-0.334563
C	-3.553571	-0.604967	1.700859
C	3.998096	0.025224	-0.372495
C	4.425352	1.503275	-0.416959
C	3.672217	-0.406974	-1.807331
C	5.239777	-0.753058	0.104865
O	-0.299661	-1.378824	-2.502194
O	-2.547616	-1.524942	-0.854878
O	1.475181	2.327041	-1.179194
O	0.043491	3.717752	0.929802
O	0.026259	1.727577	1.965774
O	-0.361722	-0.517279	1.814546
O	0.471779	-2.982191	-0.025414
O	-1.380322	-3.200291	1.217076
O	-3.671038	2.120437	-0.704222
O	-1.102283	1.799628	-0.940320
H	0.611710	0.425422	-2.337631
H	-2.056411	0.470090	-2.203947
H	1.746006	-2.175756	-1.370293
H	1.984854	-1.983443	1.657419
H	3.280132	-2.449911	0.562992
H	3.235261	0.057695	1.622421
H	2.393584	2.311510	0.699786
H	1.253608	0.292227	2.712551
H	-2.359830	1.130936	1.194329
H	-4.213169	-0.031629	2.359660
H	-2.804150	-1.118171	2.310201
H	-4.157553	-1.355037	1.187226
H	3.703069	2.131452	-0.942341
H	5.369267	1.572914	-0.970650
H	4.587929	1.912879	0.583481
H	3.583070	-1.493132	-1.903416
H	4.482174	-0.089347	-2.474115
H	2.749648	0.061197	-2.165273
H	5.511854	-0.466188	1.127517
H	6.089895	-0.518397	-0.545027
H	5.103196	-1.837213	0.078930
H	0.532861	2.507602	-1.406407
H	-1.149650	-1.744740	-2.186931
H	-3.478921	-1.220209	-1.009483
H	-2.027105	2.187357	-0.773878
O	-4.743863	0.136111	-0.799529
O	2.920390	2.874646	1.935049
H	2.370128	3.684322	1.899063

•OOH

ν_{im}= -1997.6109 cm^{-1}

C	0.018043	-0.411261	-1.675501
C	-1.308242	0.290843	-1.369318
C	-1.873627	-0.521834	-0.164812
C	-0.604142	-1.002404	0.602177
C	0.622478	-0.688989	-0.275512
C	1.431363	-2.000715	-0.165014
C	2.492354	-1.724185	0.901532
C	2.871006	-0.242466	0.731070
C	1.454335	0.396564	0.501988
C	1.416257	1.849790	0.050976
C	0.363196	2.563245	0.843499
C	0.641573	0.515575	1.845807
C	-0.571920	-2.520706	0.840086
C	-2.881485	0.276245	0.666951
C	-3.878880	0.784631	-0.410771
C	-3.533319	-0.582919	1.745013
C	4.006868	-0.005943	-0.326283
C	5.217729	-0.867190	0.081758
C	4.502015	1.450221	-0.331891
C	3.617920	-0.365011	-1.765552
O	-0.212522	-1.633417	-2.347478
O	-2.482121	-1.684449	-0.722361
O	1.508938	2.193314	-1.264214
O	-0.001748	3.701465	0.692583
O	0.024754	1.805909	1.895452
O	-0.349253	-0.447180	1.890295
O	0.541646	-3.034896	0.262583
O	-1.328290	-3.178152	1.489080
O	-3.668856	1.941151	-0.867102
O	-1.086449	1.646838	-1.058111
H	0.667863	0.193449	-2.309116
H	-1.999841	0.198919	-2.216359
H	1.824809	-2.325280	-1.125533
H	2.009733	-1.864336	1.877780
H	3.330424	-2.420461	0.860603
H	3.272314	0.157486	1.673097
H	2.391145	2.471416	0.705993
H	1.249119	0.431292	2.751525
H	-2.360410	1.126864	1.113772
H	-4.115648	-1.383782	1.286013
H	-4.213080	0.029499	2.345542
H	-2.784570	-1.030543	2.404732
H	5.485369	-0.703866	1.132763
H	6.083705	-0.593286	-0.530229
H	5.044657	-1.936567	-0.065849
H	3.784397	2.138417	-0.784110
H	5.415658	1.507897	-0.934924
H	4.739336	1.802246	0.675871
H	3.410176	-1.430645	-1.893257
H	4.453170	-0.119665	-2.432250
H	2.752913	0.214923	-2.097391
H	0.587154	2.412141	-1.535021
H	-1.063046	-1.985653	-2.018227

H	-3.416310	-1.406901	-0.910353
H	-2.018131	2.028315	-0.930995
O	-4.700516	-0.066738	-0.822335
O	2.274179	3.696807	2.398259
H	1.633080	4.319955	2.001220
O	3.053987	3.320382	1.328063

$^{\bullet}OCH_3$

ν_{im}= -1537.6790 cm^{-1}

C	-0.031584	-0.191482	-1.726457
C	-1.353850	0.486009	-1.356341
C	-1.922884	-0.426678	-0.228388
C	-0.656401	-0.978613	0.493319
C	0.570360	-0.602310	-0.358327
C	1.361700	-1.928160	-0.376599
C	2.443517	-1.767116	0.693825
C	2.835428	-0.278075	0.665747
C	1.421865	0.392825	0.505434
C	1.405848	1.894046	0.204570
C	0.355674	2.532961	1.082786
C	0.632226	0.390950	1.870823
C	-0.638364	-2.512048	0.598916
C	-2.923749	0.303002	0.671794
C	-3.919060	0.909903	-0.355342
C	-3.579937	-0.641546	1.672896
C	3.984849	0.019798	-0.361293
C	4.402543	1.499271	-0.404528
C	3.655911	-0.410505	-1.796019
C	5.233002	-0.753148	0.107631
O	-0.269336	-1.341497	-2.513461
O	-2.538000	-1.532362	-0.885613
O	1.457092	2.372832	-1.076047
O	-0.068891	3.650454	1.003705
O	0.070889	1.684192	2.087794
O	-0.397683	-0.534949	1.821517
O	0.463701	-2.986052	-0.033143
O	-1.395334	-3.216277	1.196307
O	-3.703362	2.100613	-0.708453
O	-1.119938	1.803447	-0.919886
H	0.624140	0.468269	-2.297270
H	-2.046588	0.477175	-2.207390
H	1.737021	-2.165578	-1.369162
H	1.980696	-2.007242	1.659902
H	3.273990	-2.461529	0.557913
H	3.238533	0.032949	1.639944
H	2.359200	2.328863	0.840238
H	1.250632	0.170947	2.745949
H	-2.394135	1.107143	1.189757
H	-4.255710	-0.078729	2.324335
H	-2.833320	-1.148153	2.291008
H	-4.167319	-1.396663	1.147454
H	3.659331	2.125320	-0.903530
H	5.330533	1.579337	-0.983244
H	4.586079	1.898017	0.596707
H	3.573374	-1.496766	-1.895339
H	4.461656	-0.085893	-2.464438
H	2.729144	0.052066	-2.150010
H	5.510287	-0.466019	1.128791
H	6.077977	-0.513952	-0.547372
H	5.100977	-1.837912	0.081644
H	0.517700	2.570264	-1.304870
H	-1.119100	-1.721266	-2.214212
H	-3.470887	-1.233594	-1.046142
H	-2.045341	2.183829	-0.755834
O	-4.745505	0.101767	-0.839405
O	2.989060	3.119218	1.739257
C	3.083947	4.338265	1.067935
H	2.119774	4.627190	0.627105
H	3.334874	5.093069	1.827462
H	3.865945	4.336596	0.294698

$^{\bullet}OOCH_3$

ν_{im}= -2011.6274 cm^{-1}

C	0.075360	-0.418015	-1.664509
C	-1.266001	0.267715	-1.392861
C	-1.855880	-0.553434	-0.205965
C	-0.603862	-1.027647	0.593249
C	0.642050	-0.698972	-0.250064
C	1.459108	-2.006875	-0.123753
C	2.491249	-1.721171	0.965992
C	2.864121	-0.240003	0.789593
C	1.443391	0.388874	0.556330
C	1.397554	1.856949	0.143159
C	0.310463	2.537295	0.926582
C	0.613668	0.475762	1.893769
C	-0.567273	-2.547237	0.823131
C	-2.890965	0.234036	0.601814
C	-3.864950	0.737934	-0.499251
C	-3.563279	-0.634062	1.660010
C	3.991763	0.006283	-0.277413
C	5.164828	-0.940391	0.039190
C	4.551579	1.435863	-0.184871
C	3.571051	-0.216147	-1.736248
O	-0.120995	-1.637445	-2.352767
O	-2.439389	-1.718422	-0.785089
O	1.522185	2.252234	-1.154363
O	-0.137315	3.634765	0.737875
O	0.003473	1.764100	1.981011
O	-0.387468	-0.481702	1.891593
O	0.568272	-3.049644	0.278665
O	-1.337178	-3.214992	1.445222
O	-3.656432	1.898724	-0.943709
O	-1.060995	1.622874	-1.072506
H	0.735745	0.200433	-2.273716
H	-1.934110	0.171667	-2.258233
H	1.880658	-2.328811	-1.073296
H	1.984390	-1.852164	1.931276
H	3.333194	-2.413640	0.955730
H	3.265371	0.163601	1.728856
H	2.361825	2.384289	0.889574
H	1.210448	0.353960	2.802151
H	-2.387782	1.087139	1.064965
H	-4.263502	-0.029388	2.244705
H	-2.827992	-1.077505	2.337391
H	-4.126580	-1.438149	1.183151
H	5.437352	-0.899079	1.100887
H	6.042778	-0.643658	-0.544594
H	4.942255	-1.980022	-0.217821
H	3.842225	2.187722	-0.537129
H	5.437781	1.511334	-0.825941
H	4.851259	1.687695	0.836991
H	3.236252	-1.236514	-1.937824
H	4.436338	-0.030101	-2.384228
H	2.785740	0.484304	-2.027703
H	0.599152	2.442614	-1.446224
H	-0.976355	-2.001061	-2.049478
H	-3.370647	-1.446872	-0.996074
H	-1.994465	2.000121	-0.961532
O	-4.666040	-0.120877	-0.936875
O	2.240249	3.677669	2.513932
O	3.069840	2.844970	1.807847
C	2.285874	4.964342	1.912430
H	1.953356	4.902613	0.872803
H	1.584196	5.573069	2.485061
H	3.000378	5.370502	1.975542

$^{\bullet}OOCH=CH_2$

ν_{im}= -2007.5302 cm^{-1}

C	0.003177	-0.473573	-1.732354
C	-1.301230	0.306686	-1.548260
C	-1.882029	-0.272939	-0.222529
C	-0.622525	-0.653483	0.613620
C	0.607099	-0.530454	-0.305873
C	1.376295	-1.830833	0.023818
C	2.454143	-1.409274	1.022122
C	2.881017	0.003650	0.588320
C	1.479178	0.643319	0.279230
C	1.467430	2.010032	-0.400829
C	0.429318	2.868771	0.271137
C	0.691033	0.999108	1.597491
C	-0.635266	-2.110155	1.105021
C	-2.859880	0.685442	0.462847

C	-3.850801	1.031214	-0.683129
C	-3.527920	0.044018	1.674225
C	3.993281	0.010031	-0.521888
C	4.582220	1.414636	-0.734330
C	3.541117	-0.501819	-1.895540
C	5.155869	-0.874782	-0.032634
O	-0.264216	-1.784017	-2.190445
O	-2.527189	-1.494733	-0.573765
O	1.561071	2.140393	-1.750474
O	0.038112	3.945612	-0.077245
O	0.134757	2.309240	1.461466
O	-0.344914	0.101746	1.789299
O	0.459143	-2.748774	0.623419
O	-1.407432	-2.624647	1.856441
O	-3.613101	2.086824	-1.329725
O	-1.037879	1.687672	-1.470103
H	0.665776	-0.000888	-2.458151
H	-2.000102	0.092910	-2.366874
H	1.752007	-2.327855	-0.867331
H	1.974857	-1.354406	2.008540
H	3.267482	-2.130248	1.106746
H	3.320875	0.542448	1.438583
H	2.450592	2.654625	0.174594
H	1.306990	1.020121	2.501338
H	-2.310426	1.584234	0.755518
H	-4.136533	-0.806071	1.360920
H	-4.185343	0.770390	2.162094
H	-2.787279	-0.305464	2.399125
H	3.886785	2.088258	-1.239701
H	5.466582	1.330865	-1.376857
H	4.886858	1.875224	0.209140
H	3.222067	-1.546789	-1.879334
H	4.385578	-0.436915	-2.592260
H	2.735404	0.115179	-2.299687
H	5.455514	-0.611024	0.988956
H	6.024158	-0.730716	-0.684412
H	4.909984	-1.940117	-0.054198
H	0.647062	2.361721	-2.048016
H	-1.122136	-2.051897	-1.805713
H	-3.454506	-1.225449	-0.803879
H	-1.955279	2.114772	-1.405631
O	-4.698190	0.145077	-0.941534
O	3.003840	2.993905	2.177202
O	3.148240	3.450999	0.885596
C	2.149012	3.807951	2.886229
H	1.583683	4.493812	2.263425
C	2.064890	3.683841	4.203696
H	2.670702	2.963801	4.742123
H	1.364225	4.295792	4.755475

Table S15 Coordinates of the structures of the five ROSs, their anion and protonated forms. Level of theory: M06-2X/6-31G(d).

$^{\bullet}OH$

O	-0.006151	0.000000	-1.510008
H	-0.006151	0.000000	-2.488867

OH^-

O	-0.550614	0.488866	-2.277544
H	-0.550614	0.488866	-1.295288

HOH

O	0.019792	-0.005769	-1.526814
H	0.002994	-0.002034	-2.492171
H	-0.889189	0.196357	-1.271714

$^{\bullet}OOH$

O	0.055503	0.709875	0.000000
O	0.055503	-0.601901	0.000000
H	-0.888054	-0.863793	0.000000

OOH^-

O	2.522055	0.157483	-1.973079
O	2.525399	0.629765	-0.561396
H	3.346251	0.215549	-0.260456

HOOH

O	0.000000	0.713705	-0.055572
O	0.000000	-0.713705	-0.055572
H	-0.812252	-0.891397	0.444578
H	0.812252	0.891397	0.444578

$^{\bullet}OCH_3$

C	-0.009738	-0.577810	0.000000
H	1.056999	-0.867510	0.000000
H	-0.460335	-1.006509	0.905992
H	-0.460335	-1.006509	-0.905992
O	-0.009738	0.793424	0.000000

OCH_3^-

O	2.523901	0.154594	-1.978199
C	2.524112	-0.263964	-3.222639
H	3.546534	-0.440273	-3.721296
H	2.004624	-1.267193	-3.443408
H	2.020340	0.411634	-4.007467

$HOCH_3$

C	-0.046785	0.658091	0.000000
H	-1.091646	0.973590	0.000000
H	0.437523	1.077763	0.891937
H	0.437523	1.077763	-0.891937
O	-0.046785	-0.753213	0.000000
H	0.871595	-1.051960	0.000000

$^{\bullet}OOCH_3$

O	1.169320	-0.278307	0.000017
O	0.157975	0.542855	-0.000016
C	-1.081506	-0.181434	-0.000021
H	-1.130743	-0.801366	-0.896590
H	-1.131372	-0.800256	0.897305
H	-1.867213	0.573839	-0.000590

$OOCH_3^-$

O	2.494591	-0.199201	-1.843874
C	2.559683	-0.338526	-3.204296
H	3.586722	-0.576807	-3.550937
H	1.882866	-1.157251	-3.525383
H	2.263238	0.590156	-3.734480
O	3.418155	0.893252	-1.544405

$HOOCH_3$

O	-1.147069	0.270796	0.102545
O	-0.025279	-0.605400	0.019773
H	-1.569086	0.104132	-0.756207
C	1.115476	0.222340	-0.021549
H	1.963984	-0.466065	-0.031970
H	1.125724	0.842163	-0.925757
H	1.165302	0.862565	0.864682

$^{\bullet}OOCH=CH_2$

C	-1.389731	-0.500063	0.000186
C	-0.628608	0.583193	-0.000384
H	-0.954065	-1.490586	0.001155
H	-2.466422	-0.390841	-0.000307
H	-0.972696	1.610046	-0.001325
O	0.762674	0.580046	0.000699
O	1.300227	-0.608472	-0.000491

$OOCH=CH_2^-$

O	2.533068	0.394032	-2.323683
O	3.558455	-0.554886	-1.974332
C	1.497424	-0.171810	-2.903664
H	0.738295	0.580351	-3.155545
C	1.295730	-1.471999	-3.192864
H	2.059177	-2.190542	-2.934028
H	0.375610	-1.781727	-3.678703

$HOOCH=CH_2$

C	1.406517	-0.554260	0.001289
C	0.696641	0.565615	0.013829
H	0.940238	-1.527673	-0.060358
H	2.485961	-0.488479	0.033521
H	1.124964	1.562825	0.054755
O	-0.663060	0.702224	-0.016204
O	-1.287489	-0.566962	-0.101344
H	-1.565717	-0.696899	0.821765
